# Relevance of Probabilistic Reversal Learning for Adolescent Drinking Trajectories

**DOI:** 10.1111/adb.70026

**Published:** 2025-03-06

**Authors:** Juliane H. Fröhner, Maria Waltmann, Andrea M. F. Reiter, Anja Kräplin, Michael N. Smolka

**Affiliations:** ^1^ Department of Psychiatry and Psychotherapy Technische Universität Dresden Dresden Germany; ^2^ Department of Child and Adolescent Psychiatry, Psychosomatics and Psychotherapy University Hospital Würzburg Würzburg Germany; ^3^ Department of Neurology Max‐Planck‐Institute for Human Cognitive and Brain Sciences Leipzig Germany; ^4^ Department of Psychology Julius‐Maximilians‐University of Würzburg Würzburg Germany; ^5^ Department of Psychology Technische Universität Dresden Dresden Germany

**Keywords:** adolescence, alcohol, reversal learning

## Abstract

One of the many human capabilities acquired during adolescence is the adaptivity in changing environments. In this longitudinal study, we investigated this adaptivity, as measured by probabilistic reversal learning (PReL) tasks, in *N* = 143 adolescents at ages 14, 16 and 18. Computational modelling and functional magnetic resonance imaging were applied to identify the neurocognitive processes underlying reversal learning and its development. Previous studies have demonstrated a correlation between heavy alcohol use and impaired reversal learning. Our hypothesis was that PReL is negatively associated with current and future alcohol use and that alcohol use impairs PReL by altering neurocognitive processes. Behaviourally, PReL performance improved, which was associated with a lower probability of switching choices and was considered an adaptive process. Computationally, this was accounted for by higher learning rates, enhanced sensitivity to wins and reduced sensitivity to losses in older adolescents. Alcohol consumption increased but remained at a low level for most participants. More risky drinking was associated with less medial frontal activity elicited by reward prediction errors. These findings suggest that reversal learning may be more relevant for the maintenance or escalation of risky than for low‐level drinking. Challenges and potential solutions for longitudinal studies such as reliability are discussed.

## Graphical abstract

We investigated the development of probabilistic reversal learning (PReL) in 143 adolescents (ages 14, 16, and 18) using computational modeling and fMRI. PReL performance improved with age, driven by enhanced reward sensitivity, and reduced sensitivity to losses and probability to switch. Risky drinking was associated with lower medial frontal activity during feedback processing.

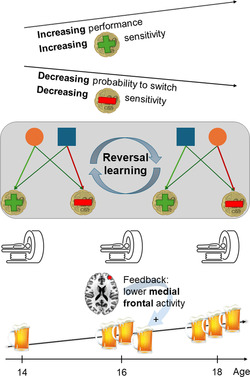

## 1 Introduction

The capacity to adapt flexibly to environmental changes is a fundamental aspect of human behaviour that matures during adolescence—a period marked by impulsive and risky behaviours such as heavy alcohol use [1, 2]. We longitudinally investigated cognitive and behavioural adaptivity in adolescents aged 14 to 18 using a probabilistic reversal learning (PReL) task. Participants learned which of two stimuli were more likely to yield rewards, with these associations eventually reversing. Combining behavioural analyses with computational modelling and functional magnetic resonance imaging (fMRI), we aimed to understand the learning processes and their neural correlates. We hypothesized that less adaptive learning would be associated with increased drinking during adolescence.

Alcohol use typically begins in adolescence, and neurobiological changes during this period can predispose individuals to alcohol‐related disorders, which are highly prevalent among adolescents [3, 4]. Addiction research has identified numerous risk factors for alcohol use disorder (AUD; [5]). This study focuses on alterations in learning mechanisms, with computational modelling—particularly reinforcement learning (RL) models—serving as a framework to describe the cognitive processes underlying behaviour. Prior studies in adults have linked AUD and addiction to disrupted RL processes and impaired behavioural flexibility (e.g., [6, 7]). Key concepts in RL include prediction error (PE)—the difference between actual and expected outcomes—and learning rate, which reflects how quickly expectations are adjusted based on the PE [8, 9, 10]. In classical models, choice stochasticity measures the extent to which an individual's decisions depend on the prediction error when selecting an action [8]. In alternative models, reinforcement sensitivity quantifies the impact of feedback by directly scaling the perceived value of the received feedback [11]. Recent approaches also differentiate between the single‐update (SU) strategy, which updates only the chosen option's expected value according to the PE, and the double‐update (DU) strategy, which updates both the chosen and unchosen options' expected values [7, 12, 13]. This means, the more participants engage in DU, the more they consider the counterfactual nature of the task, i.e., the feedback for one stimulus also contains information about the other stimulus. AUD patients did less DU, especially after punishment, and recruited less activity in the medial prefrontal cortex (PFC) related to the DU‐PE, which correlated with their drinking habits [7]. In a similar vein, other studies associated alterations of neural learning signals in alcohol‐dependent patients with their alcohol intake (alcohol dependence according to DSM‐IV; [6, 14]). In addition to these patient studies, it has been shown that binge drinkers showed reduced reversal learning performance [15]. Thus, there is converging evidence about impaired reversal learning in high‐risk drinkers that is associated with altered neurocognitive processes, but cross‐sectional studies cannot distinguish between the consequences of chronic alcohol abuse and pre‐existing risk factors. Our longitudinal study aimed to investigate whether altered reinforcement learning processes could act as a pre‐existing risk factor for problematic alcohol use, even in a sample of social‐drinking adolescents.

To evaluate this potential association with alcohol use, it is important first to understand the development of RL processes during adolescence. Developmental PReL studies are rare and findings on performance differences between adolescents and adults remain mixed. There were no performance differences in probabilistic as well as deterministic reversal learning ([16, 17]). However, a large cross‐sectional study showed increasing performance from childhood to adulthood with a peak in adolescence [18]. Computational studies have also revealed developmental differences in learning processes. Learning rates after punishment decrease from childhood to adulthood [16], while learning from positive feedback increases in general (not reversal specific; [19]). Positive feedback had less impact on adolescents, which might explain more choice switches [11]. Our previous study found adolescents exhibited more explorative behaviour, shown by a lower beta parameter of the sigmoid function [20]. Developmental neurocognitive studies present a mixed picture. The insula showed higher activity after negative feedback during adolescence [16], but other studies found no difference in PE‐signal between adolescents and adults [19, 21]. A recent cross‐sequential study showed reduced medial frontopolar cortex activity in adolescents, indicating less confidence in upcoming choices [11]. Feedback processing in cortical and subcortical regions changes during adolescence, but the directionality remains debated, and longitudinal evidence on (neuro)cognitive RL processes is lacking (see [22] for a review).

This is the first longitudinal study to link PReL and alcohol consumption in adolescence, examining whether impaired reversal learning, as observed in AUD, is a risk factor preceding high‐risk alcohol use or a consequence of chronic alcohol abuse (e.g., [7]). We hypothesize that an imbalance between less developed frontal regulation and greater subcortical approach behaviour underlies both adolescent alcohol use and PReL performance [23, 24]. This approach behaviour may hinder feedback learning in a PReL task and promote alcohol consumption, potentially affecting neural signalling in the vulnerable adolescent brain [25]. This first longitudinal fMRI study of PReL and alcohol use began before the publication of many of the cited works and the applied methods. While the basic assumption was that better PReL performance would be associated with less overall drinking and less escalation over time, the specific operationalizations of variables and the detailed analytical approaches were not fully established at the study's outset.

## 2 Materials and Methods

### 2.1 Participants

Participants were recruited at local schools at the age of 14 as part of a larger longitudinal study “The adolescent brain”. Details on recruitment, exclusion criteria, and the full protocol are available elsewhere [26]. This study focuses on the PReL task, associated fMRI data, and self‐reported alcohol use. After fMRI data quality control, the final sample included 143 subjects (73 male) who completed the PReL task at ages 14, 16, and 18 (see Table 1 for sample description).

**TABLE 1:** Sample description.

Acquisition wave (target age in years)	1 (14)			2 (16)			3 (18)		
	Mean	SD	Range	Mean	SD	Range	Mean	SD	Range
Exact age in years	14.6	0.33	13.7–15.1	16.6	0.36	15.7–17.4	18.6	0.45	17.8–20.5
SES^a^	10	3.2	4–20	—			—		
Gram alcohol drunk^b^	5.2	38.7	0–461.5	24.9	41.9	0–318.9	52.3	61.7	0–345.9
AUDIT total score	0.9	1.48	0–10	3.2	2.87	0–15	4.2	2.97	0–13
Hazardous drinking^c^	1.4%			5%			7%		
No. of daily smokers (%)	1 (1%)			6 (4%)			20 (14%)		
Cigarettes smoked^b^	1	8.9	0–101	3	13	0–102	10	22.4	0–105
No. of cannabis users (%)	1 (1%)			6 (4%)			20 (14%)		
Days cannabis used^b^	0.01	0.06	0–0.75	0.07	0.64	0–7.25	0.21	0.92	0–6.75

^a^ Socioeconomic status: A sum score of several factors acquired within the online questionnaires of the IMAGEN study in the first acquisition wave, referring to parental education, family stress due to financial reasons and neighbourhood. The values can range from 1 to 23, whereby a lower value represents a higher socioeconomic status.

^b^ Per week; acquired via the TimeLineFollowBack interview AUDIT: Alcohol Use Disorder Identification Test.

^c^ Percentage of participants with AUDIT score higher than 8.

### 2.2 Alcohol Use

To assess drinking behaviour, we used the Alcohol Use Disorder Identification Test (AUDIT; [27]), which measures the quantity and the frequency, as well as the hazardousness of a person's alcohol use (AUDIT total score). We used the AUDIT total score as the outcome measure to align with previous research that links reinforcement learning deficits to AUD risk rather than focusing solely on drinking quantity. While the AUDIT‐C assesses frequency and quantity of alcohol consumption, its strong correlation with the AUDIT total score in our sample (*r* = 0.92–0.96 across ages) indicates that drinking behaviour in this cohort was neither risky nor problematic. This is further supported by an average AUDIT‐P score below 1, reflecting an almost complete absence of alcohol‐related problems (age 14: 0.1; age 16: 0.5; age 18: 0.7). Moreover, using the AUDIT total score ensures consistency with our previous analyses [28].

Additionally, we derived the quantity of alcohol use in gram per week from the Timeline Follow Back interview (TLFB; [29]). During the interview, participants were asked how much and what they drank in the past 30 days. Using the grams per week measure, we estimated cumulative alcohol consumption via area under the curve (linear interpolation). Higher cumulative alcohol consumption indicates greater alcohol exposure during the study period.

### 2.3 Paradigm

**FIGURE 1:**
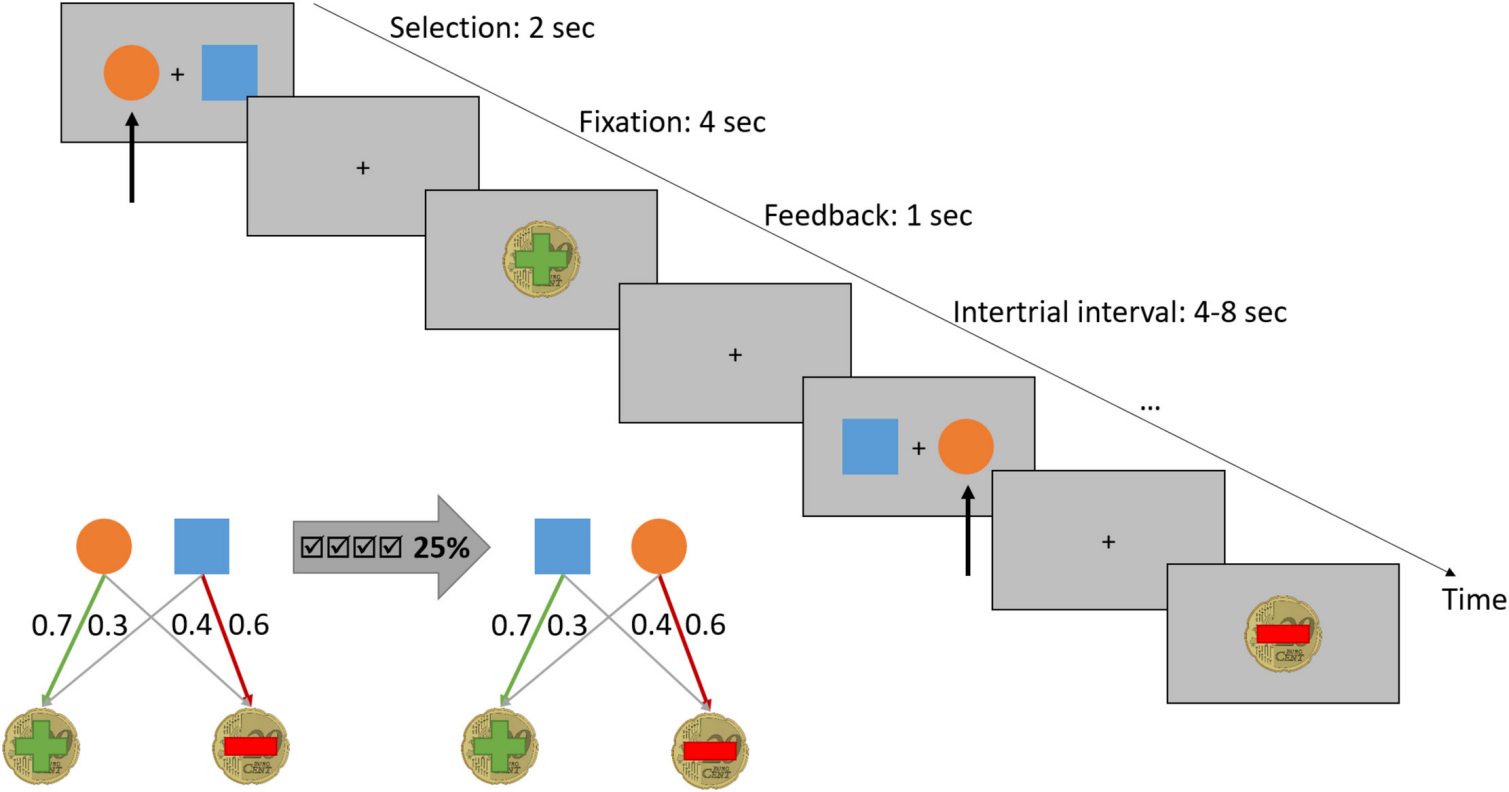
Overview of the used probabilistic reversal learning task. After four consecutive correct trials, a contingency change occurred in 25% of the cases.

We used a PReL task similar to the one introduced by Hampton and O'Doherty [30]. In each trial, participants choose between two stimuli (Figure 1). Each stimulus was associated with a certain probability of winning or losing 20 cents (70%–30% and 40%–60%, respectively). At the end of each trial, participants receive feedback about their choice (win or loss) as well as about their current total score. Participants were instructed to maximize their gain, which in turn increases their participation fee. When participants consecutively choose the more beneficial stimulus four times, the reward contingencies reverse with a 25% probability. In total, participants completed 120 trials. The task duration was approximately 26 min. Prior to the scanning session, participants received thorough training for the task (please refer to [20] for a more detailed task description). Given the adaptive nature and the high difficulty of the task, we prioritized the inclusion of participants who completed all three sessions and did not apply other exclusion criteria.

#### 2.3.1 Behavioural Parameters

To analyse task performance, we extracted three performance metrics: obtained reward, accuracy and the number of contingency changes. The obtained reward is the feedback guiding participants' behaviour. Accuracy measures the proportion of trials where the more beneficial stimulus was chosen, regardless of feedback, and is moderately correlated with the obtained reward (see Table S2). The number of contingency changes, occurring after four consecutive correct choices, indicates task performance and difficulty, as participants must frequently reverse their learned associations. Due to the adaptive and probabilistic task design, the correlation with accuracy and reward is lower (see Table S2). Given that stay‐switch behaviour is less impacted by the adaptive design and has been previously reported, we also examined stay probabilities, i.e., the probability of repeating the previous trial's choice following a win, or a loss [7, 11, 21].

Following Waltmann et al. [31], we used mixed‐effects models to enhance parameter reliability mainly by reducing error variance (see Figures S1 and S2). Mixed‐effects models use trial‐by‐trial data to account for clustering within the data, variance heterogeneity between subjects and variability within subjects. We estimated accuracy and stay probabilities (after win and loss separately) using mixed‐effects logistic regression with a joint model for correlated random intercepts and slopes: dependent variable ~ 1 + Session + (1 + Session|Subject). We computed mixed‐effects logistic regressions in R (version 4.3.1), using the lme4 package (version 1.1‐34).

#### 2.3.2 Computational Modelling

To uncover individual differences in the underlying learning processes in PReL, we performed computational modelling and closely followed previous analyses of PReL [7, 11, 31]. We used MATLAB 2020b and the emfit toolbox to estimate parameters by maximum a posteriori estimation with empirical priors [32] and compared models according to their integrated Bayesian information criterion [33]. The DU‐model with two reinforcement sensitivities *ρ*_*win*_ and *ρ*_*loss*_ and one learning rate *α* fit the best in the joint as well as the separate estimation (DU‐2𝜌α‐model; see Figure S4), which indicates that the principal cognitive processes underlying PReL do not change during adolescence. The reinforcement sensitivity *ρ* is part of the perceptual model and provides a lower bound on choice stochasticity by determining the maximum difference between expected values (see equation (2) in the supporting information). The learning rate *α*, which signifies the weighting of recent feedback in comparison to integrated feedback from previous trials, determines how much recent trials contribute to the learning process (i.e., 1 means only considering the most recent trial). The behavioural and parameter recovery of the winning model was robust, as evidenced by moderate to high correlations between the simulated and original data, and very high correlations between refitted parameters from simulated behaviour and the originally derived parameters. For a detailed description of the computational approach, model comparison, simulation work and details on the recovery procedure, please refer to Chapter 2 in the supporting information.

To assess developmental effects on alcohol use, behavioural and computational PReL parameters, we used the mixed‐effects models. PReL variables were dependent, and time was a within‐subject factor. For stay probabilities, feedback valence was an additional within‐subject factor.

### 2.4 Associations of PREL and Drinking

#### 2.4.1 Cross‐Sectional

**FIGURE 2:**
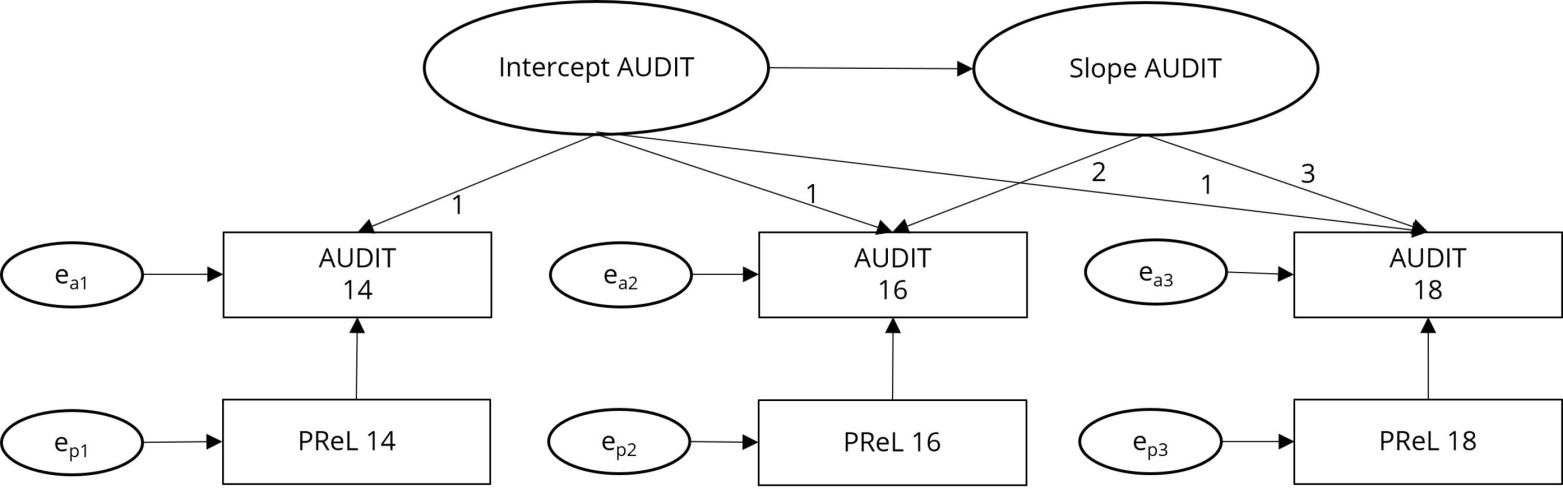
Schematic representation of the latent growth curve model (LGCM) for alcohol use as measured by the Alcohol Use Disorder Identification Test (AUDIT; [27]). Rectangles represent observed variables, while ellipses indicate latent variables estimated via the LGCM. The observed AUDIT scores serve as indicators for the latent intercept (starting point) and latent slope (change over time). Intercept regressions were fixed at 1 for all sessions, and the slopes were fixed at 2 and 3 for the second and third sessions, respectively. Behavioural and computational parameters from the probabilistic reversal learning (PReL) task were modelled as time‐varying covariates.

In line with our previous research on delay discounting and drinking [28], we aimed to compute bivariate latent growth curve models (LGCMs) to associate the development in PReL with the development in drinking. However, the bivariate models did either not converge or resulted in spurious negative variances [34], indicating poor model fit [35]. Given these issues and that the univariate AUDIT model converged, we concluded that the PReL parameters from our adaptive task were not suitable for estimating a bivariate LGCM. Therefore, we decided to compute a LGCM with intercept and slope for alcohol use and introduced PReL parameters as time‐varying predictor (see Figure 2). The AUDIT total score was employed as the drinking measure, because the combination of a quantity‐frequency measure and an indication of risky or problematic drinking aligns most closely with previous findings regarding PReL and AUD. Separate models were computed for the behavioural parameters of accuracy, stay probability after win, or loss, as well as the computational parameters learning rate and reinforcement sensitivity for wins and losses. LGCMs were estimated via the *lavaan* package implemented in R [36]. The models were fit via full information maximum likelihood estimator and fits were moderate to good (see Chapter 3 in the supporting information). The predictors and outcomes were standardized before the analyses to yield estimates that have the same range as correlation coefficients. The choice of outcome variables was of rather explorative nature; thus we considered a *p*‐value below 0.05 as significant. Please note that adding gender as a time‐invariant covariate did not significantly change the results.

#### 2.4.2 Longitudinal

To examine the long‐term influence of alcohol exposure over the course of the study, we correlated the cumulative alcohol consumption with difference in behavioural and computational PReL parameters between the ages 18 and 14.

### 2.5 Functional Magnetic Resonance Imaging

We processed the fMRI data using SPM12 (https://www.fil.ion.ucl.ac.uk/spm/software/spm12/) and MATLAB R2020b. Please refer to Chapter 4.1 in the supporting information for details of fMRI acquisition and preprocessing. In a general linear model for the first‐level statistics, we defined stimulus onset, feedback onset, missing onset and the six movement parameters. To model stimulus onset, we used the trial‐by‐trial choice probability estimated in the winning DU‐2𝜌α‐model—a function of the relative expected values of both stimuli. Thus, the higher the choice probability, the larger the difference in expected values. Therefore, choice probability can be interpreted as confidence in the following choice. Additionally, we computed a parametric regressor that represented trials that could be predicted by the model with only below‐chance accuracy (< 50%) to remove variance associated to poor model fit [11]. To model feedback onset, we computed individual SU‐ and DU‐PE such that we can differentiate between the neural correlates of actual and inferred feedback [13]. The DU‐PE regressor was built by the DU‐PE subtracted by the SU‐PE to investigate brain activation that is unique for inferred feedback. We used the winning DU‐2𝜌α‐model and the respective SU‐model to compute PEs. In both cases, we fixed reinforcement sensitivities to −1 and 1 in order to separate effects of the learning rate and reinforcement sensitivities, and to avoid problems with the estimation of the correlation between the BOLD signal and PEs [37]. At second level, we computed a full‐factorial design with acquisition wave as within‐subject factor and the first‐level contrast images as dependent variables to investigate the main effects of choice probability, SU‐ and DU‐PE.

To investigate the neural correlates of PReL, we focused on theoretically relevant regions of interest (ROIs) identified from the main effects. Signal extraction was performed using SPM's eigenvariate feature, with an 8‐mm sphere defined around the peak coordinates from the main effect analysis. Mixed‐effects regression models were employed to examine developmental trends in the ROI signals. Mirroring the structure of the behavioural analysis, we first applied latent growth curve models (LGCMs) with ROI signals as time‐varying predictors to explore cross‐sectional associations between alcohol use and neural processing during PReL. Longitudinal associations were assessed by correlating cumulative alcohol consumption with changes in ROI signals between ages 14 and 18.

In previous studies, we highlighted the issue of fMRI reliability in longitudinal research, demonstrating that prominent group‐level effects might not always stem from reliable individual‐level signals [38, 39]. Evaluating reliability is crucial, especially when investigating interindividual differences, such as parameters associated with alcohol use. Therefore, we computed intraclass coefficients (ICC) for the extracted ROI signals as a common measure of fMRI reliability [40]. To ensure that reliability values were not solely driven by developmental trajectories (e.g., low reliability due to significant changes), we also computed split‐half reliability.

## 3 Results

### 3.1 Behavioural Results

Our primary research objective was to explore the relationship between PReL and drinking behaviour during adolescence. To gain a comprehensive understanding of this association, we first examined the developmental trajectories of these variables.

#### 3.1.1 Development of Alcohol Use

**FIGURE 3:**
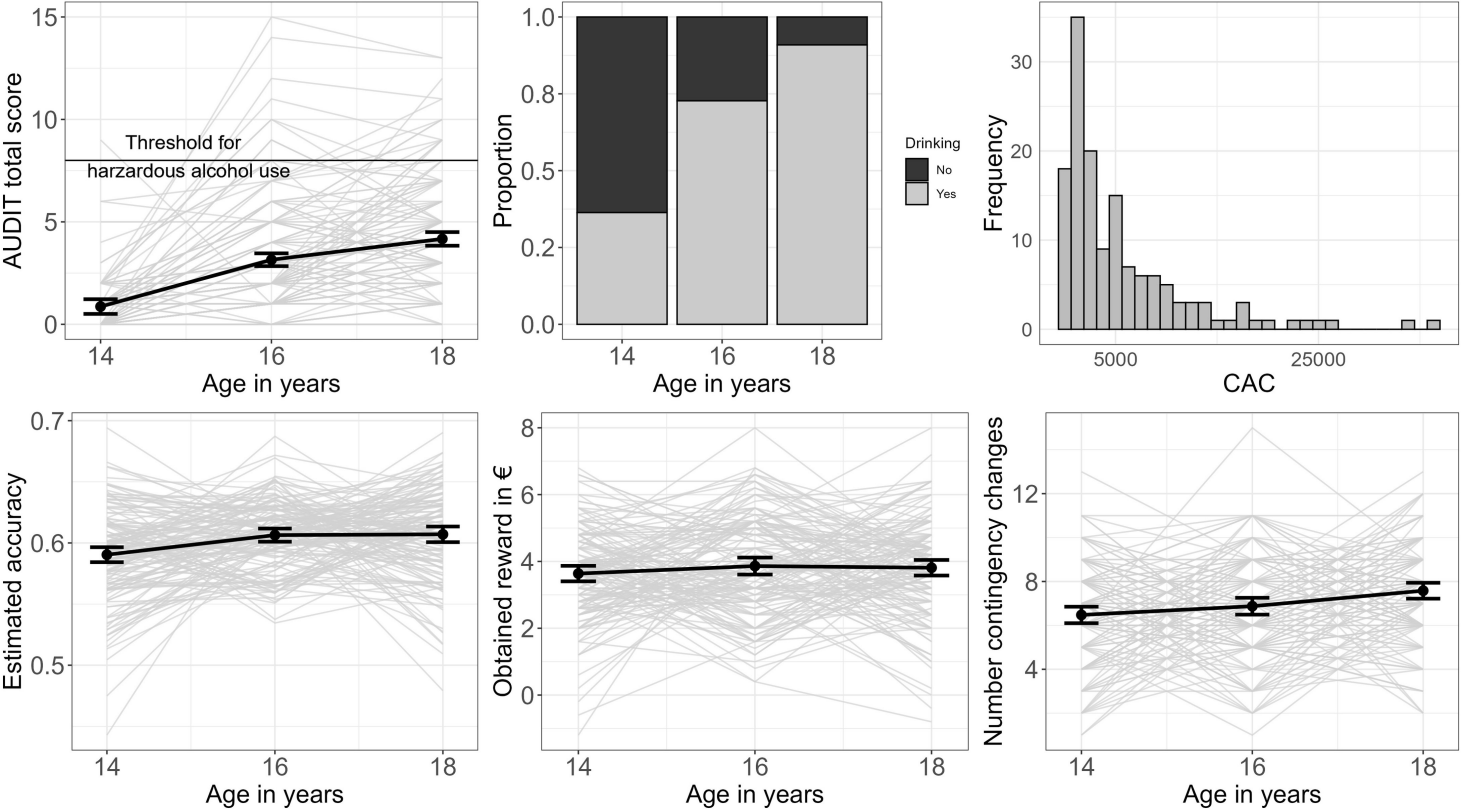
Development of alcohol use and performance measures. Upper row: development of alcohol use over time measured by Alcohol Use Disorder Identification Test (AUDIT; [27]), the proportion of participants drinking at all (light grey), and the cumulative alcohol consumption (CAC) in gram alcohol over the four study years. Lower row: development of task performance measured by estimated accuracy, obtained reward, and the number of contingency changes. Grey: individual development. Black line and error bars: average development with standard deviation.

Our previous studies demonstrated that participants' alcohol consumption increased from adolescence to young adulthood (e.g., [28]). At the beginning of the study, one half of the 14‐year‐old participants had not yet been drinking. The average total AUDIT score (*β* = 1.66, *z* = 12.21, *p* < 0.001) as well as grams alcohol drunk per week (*β* = 23.52, *z* = 7.58, *p* < 0.001; see Table 1 and Figure 3) increased significantly. Although 95% of the participants drank at age 18, only 7% of them reported a total AUDIT score of 8 or higher. Because a total AUDIT score higher than 8 represents hazardous alcohol use, we consider our sample to be low‐level drinkers [27].

#### 3.1.2 Development of Behaviour in the PREL Task

For the evaluation of PReL performance, we looked at obtained reward, number of contingency changes, and accuracy, and how these parameters changed over time (see Figure 3 and Table S1 for descriptives). On average, participants gained 3.77€ and the gained reward did not increase over time (*β* = 0.09, *z* = 1.02, *p* = 0.310). Participants completed the task with an estimated accuracy (proportion of optimal choices regardless of actual feedback) around 0.6, with 0.5 being chance level. Accuracy did not increase over time (OR = 1.02, *z* = 1.40, *p* = 0.160). The number of contingency changes increased significantly from 6.5 to 7.6 over the course of the study (*β* = 0.13, *z* = 4.13, *p* < 0.001). Thus, participants completed a more challenging version of the task in subsequent sessions with consistent accuracy, indicating an upward trend in performance. However, caution is warranted due to the absence of correlation across sessions for each of these performance measures (see Table S2).

**FIGURE 4:**
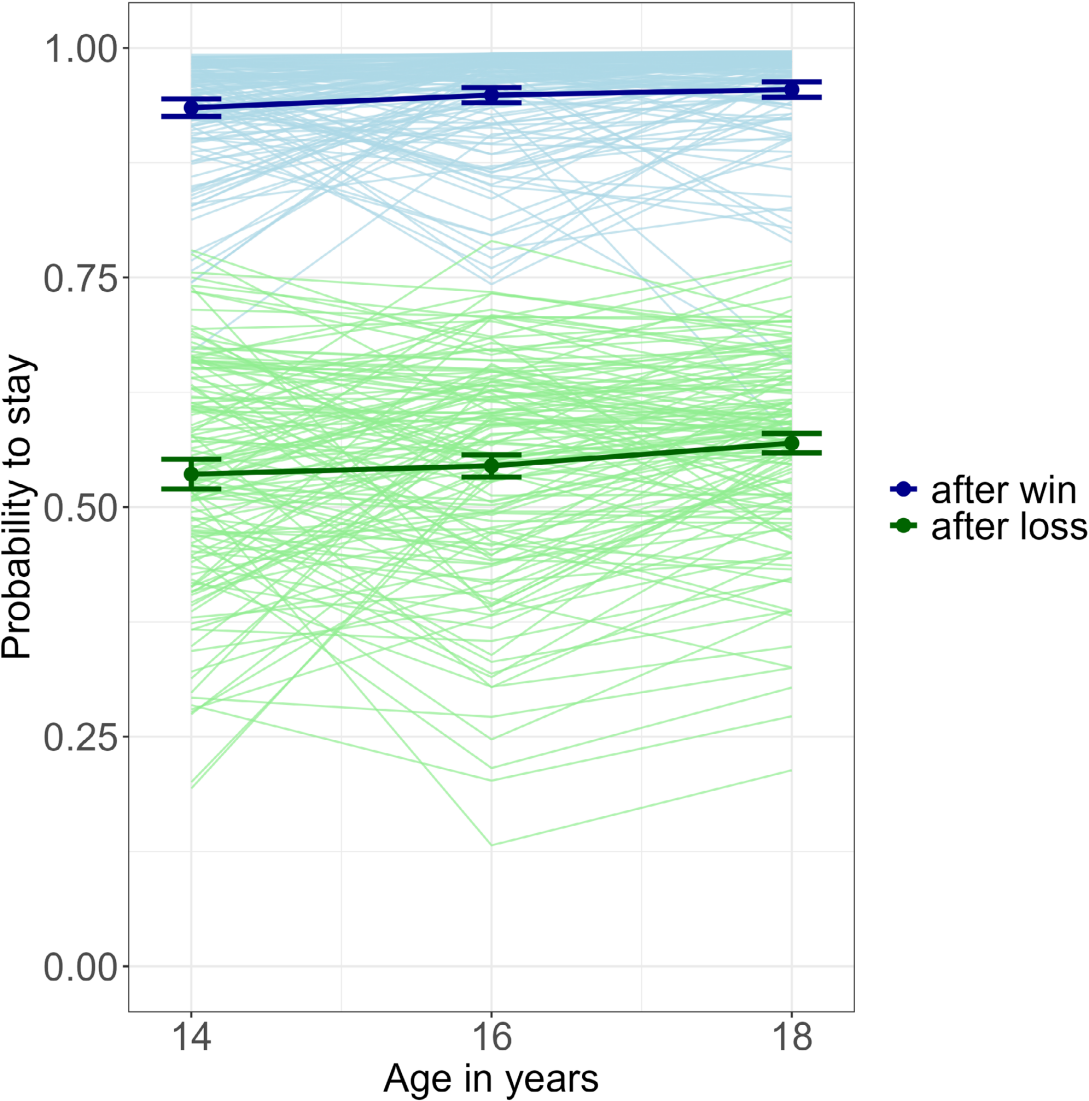
Development of the probability to stay separated by previous feedback. All values were estimated by mixed‐effects models. Thin: individual development. Thick line and error bars: average development with standard deviation.

In addition, we examined the stay‐switch behaviour of participants in terms of the probability of repeating the choice made in the previous trial (stay probability) following a win and a loss (see Figure 4). The probability to stay increased over time (OR = 1.25, *z* = 4.83, *p* < 0.001). The probability to stay after a win (around 0.94) was higher than the probability to stay after a loss (around 0.55; OR = 3.63, *z* = 17.15, *p* < 0.001). The interaction between time and feedback valence was significant (OR = 1.16, *z* = 3.94, *p* < 0.001). That is, the increase of stay probabilities after losses was more pronounced than after wins. This can be interpreted as an adaptive behaviour, since higher stay probabilities were associated with a higher number of contingency changes cross‐sectionally (*r* = 0.318–0.542, *p* < 0.001).

#### 3.1.3 Development of Computational Parameters

**FIGURE 5:**
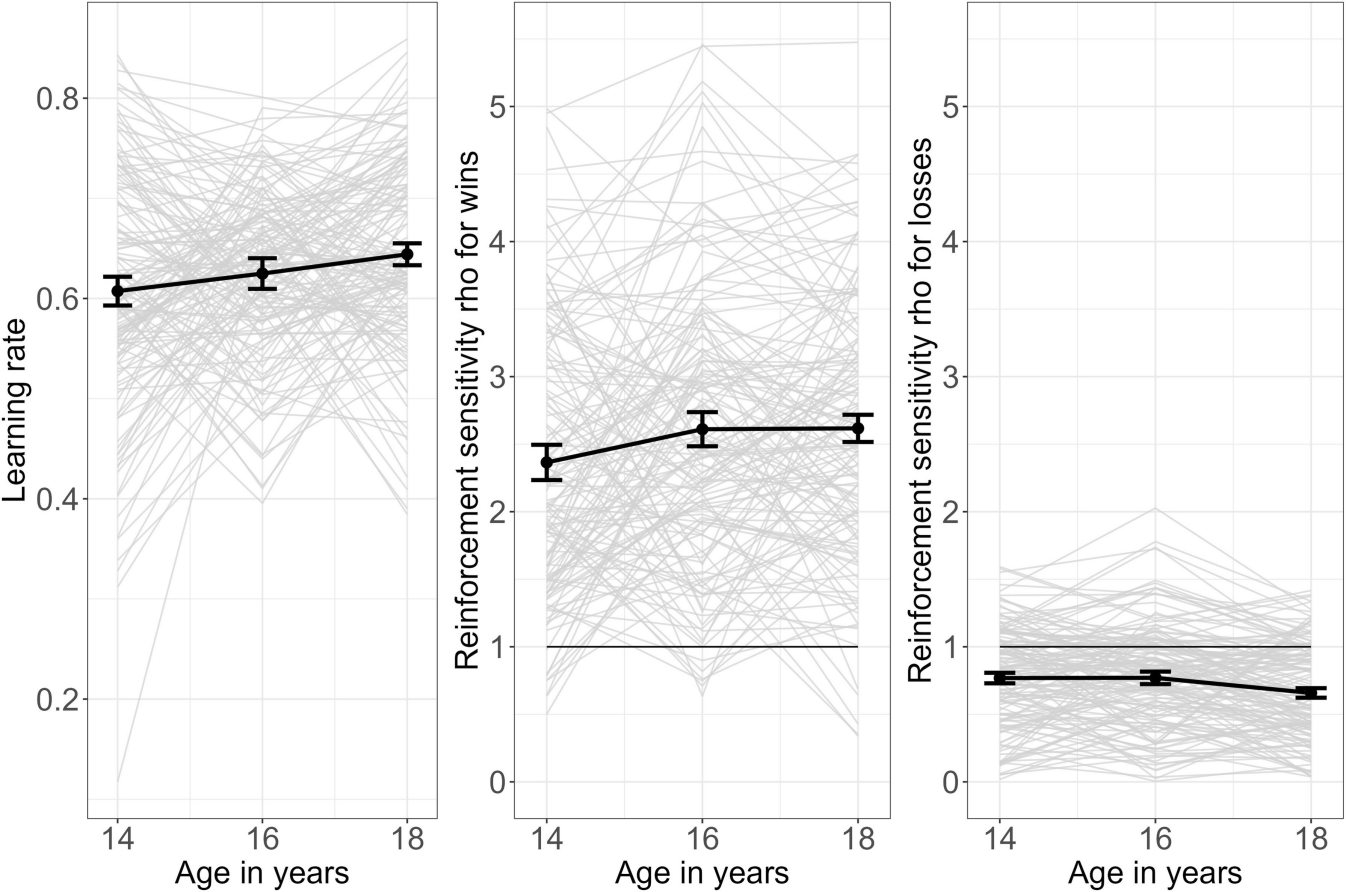
Development of computational parameters. Learning rate *α* on the left, absolute values of reinforcement sensitivities *ρ*_*win*_ in the middle and *ρ*_*loss*_ in the right panel. Grey: individual development. Thick black line and error bars: average development with standard deviation. Thin black line: reference line for actual reward value of 1.

Model comparison revealed that the reinforcement‐sensitivity DU‐model with one learning rate *α* and two reinforcement sensitivities *ρ*_*win*_ and *ρ*_*loss*_ fit the data best (see Table S6 for descriptives and correlations of computational parameters). The higher the learning rate *α*, the faster participants learn. Learning rate increased significantly from 0.61 to 0.64 (*β* = 0.02, *z* = 3.73, *p* < 0.001, see Figure 5). Thus, participants learned faster over the course of the study. Higher learning rates were mainly related to a lower probability to stay after a loss (see Table S7 for correlation of computational parameters and behaviour).

Reinforcement sensitivity reflects how a participant subjectively perceives the magnitude of a reward or loss. An increase in absolute reinforcement sensitivity amplifies the perceived value of feedback. The value 1 represents the actual reward value, i.e., the reward value is perceived as it is. Please note that in the computational model, estimated reinforcement sensitivities include the feedback value, thus *ρ*_*win*_ is mostly positive and *ρ*_*loss*_ is mostly negative (see Table S6). To ease interpretation, we used absolute reinforcement sensitivities for the following analyses. Overall, absolute reinforcement sensitivities are higher for wins (2.36–2.62) than for losses (0.66–0.77), thus our participants are more sensitive towards wins. Reinforcement sensitivity for wins increased over time (*β* = 0.13, *z* = 2.93, *p* = 0.004), i.e., participants became more sensitive for wins. Reinforcement sensitivity for losses decreased over time (*β* = 0.06, *z* = 3.78, *p* < 0.001), which means values approached zero. Thus, participants became less sensitive for losses over time, which reflects the increasing probability to stay after a loss.

#### 3.1.4 Associations of PReL Behaviour and Drinking

**FIGURE 6:**
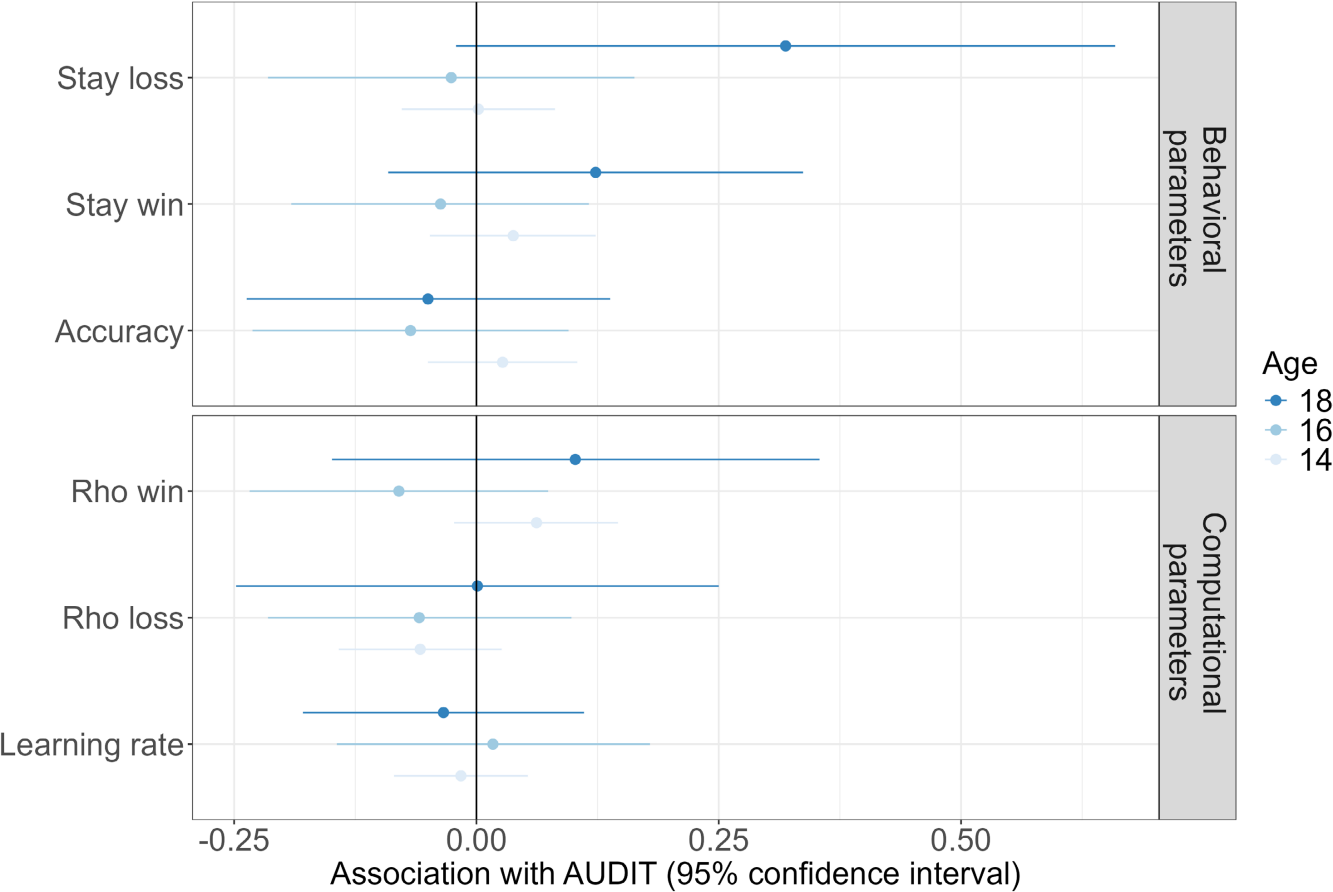
95% confidence intervals of the LGCM estimates for probabilistic reversal learning parameters (y‐axis) as time‐varying predictors of AUDIT total score (Alcohol Use Disorder Identification Test) separated by age. The behavioural parameters are estimated values from mixed‐effects models. Learning rate and the reinforcement sensitivities *ρ*_*win*_ and *ρ*_*loss*_ are extracted parameters from the computational model.

Our study aimed to examine whether poorer PReL is associated with increased alcohol use during adolescence. In a cross‐sectional analysis, we utilized LGCMs with PReL parameters included as time‐varying covariates. In total, we computed seven LGCMs for the estimated accuracy and stay probabilities as well as the computational parameters learning rate and reinforcement sensitivities (see Figure 6). To test longitudinal effects of alcohol use, we computed the correlation between changes in behavioural and computational PReL parameters and cumulative alcohol consumption over the study period.

The results of our analyses indicated that there was no significant cross‐sectional association between PReL and AUDIT scores (see Chapter 3.2 in the supporting information for detailed results of each computed LGCM). Furthermore, no significant correlations were observed between alcohol exposure and changes in PReL measures, suggesting the absence of a longitudinal association (see Table S8). These findings suggest that low‐level drinking did not influence PReL behaviour, nor was PReL behaviour associated with alcohol use.

### 3.2 fMRI Results

#### 3.2.1 Main Effects and Developmental Effects on Choice Probability and PEs

**FIGURE 7:**
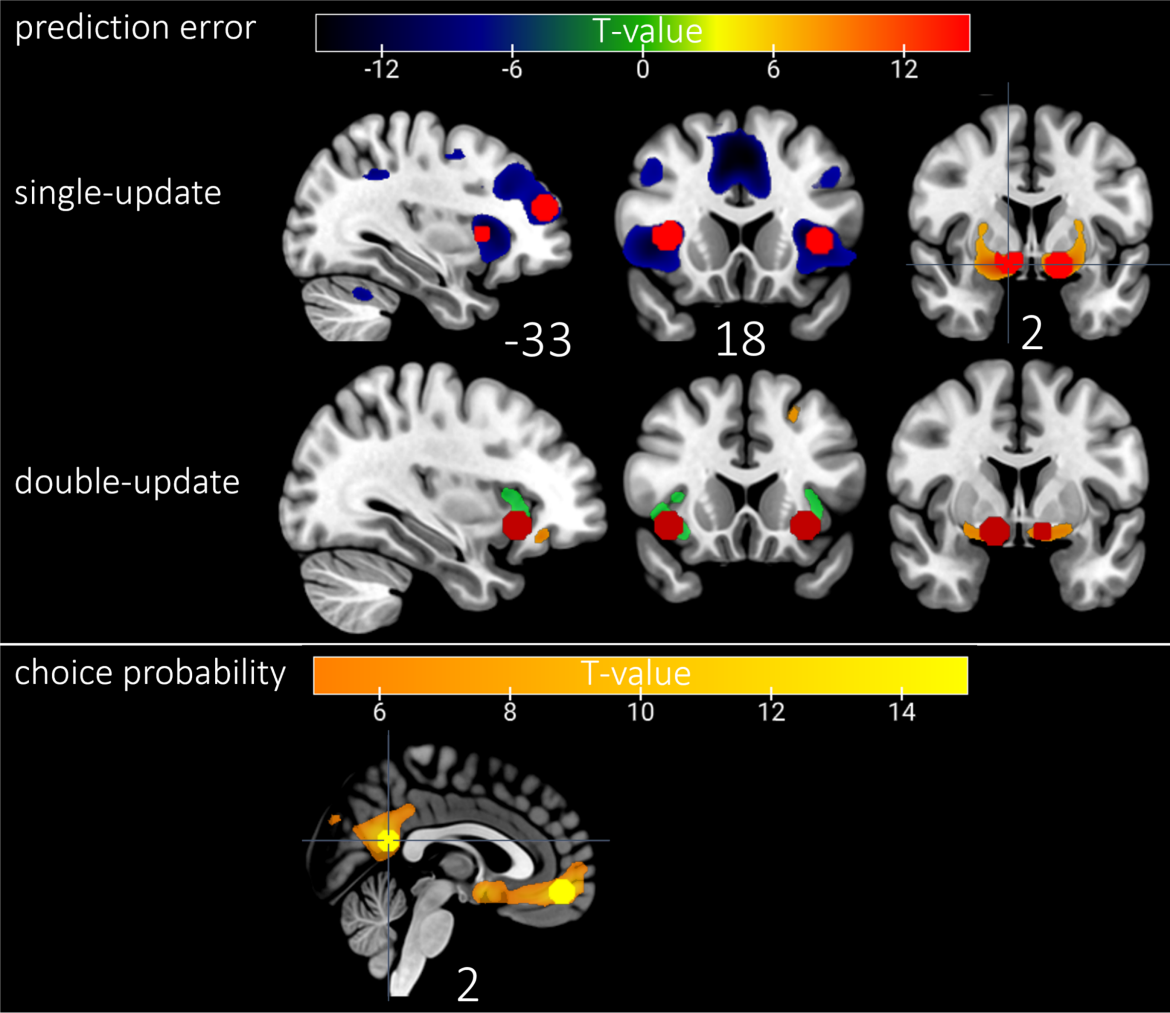
Main effects for prediction error (PE) and choice probability. Depicted are *T*‐values with *p*_*FWE‐corrected*_ < 0.05 at the peak, without cluster threshold. For each contrast, 8‐mm spheres at the peak are marked in light and dark red for PE and yellow for choice probability.

As before, we first analysed the main and developmental effects in the fMRI data to establish a clearer basis for the subsequent investigation of the associations between drinking behaviour and neural signals. Main effects were consistent with the existing literature. In the following, we summarize the main effects, please refer to Chapter 4.2 in the supporting information for the complete results table (*T*‐contrast over all sessions, *p*_*FWE‐corrected*_ < 0.05, cluster size *k* ≥ 50). Choice probability was computed within the computational model as the ratio of expected values and represented how certain subjects are about their upcoming choice. As depicted in Figure 7, higher choice probability elicited higher activity among others in ventromedial prefrontal cortex (vmPFC, *T* = 13.26, *p*_*FWE‐corrected*_ < 0.001) and posterior cingulate cortex (PCC, *T* = 14.53, *p*_*FWE‐corrected*_ < 0.001). The SU‐ and DU‐PEs as parametric modulator of feedback onset were positively associated with striatal activity (SU:*T* = 14.88, *p*_*FWE‐corrected*_ < 0.001, DU:*T* = 6.18, *p*_*FWE‐corrected*_ < 0.001) and negatively associated with the insula (SU:*T* = 13.84, *p*_*FWE‐corrected*_ < 0.001, DU:*T* = 6.87, *p*_*FWE‐corrected*_ < 0.001) and medial frontal gyrus (SU:*T* = 9.90, *p*_*FWE‐corrected*_ < 0.001); whereby double‐update effects are smaller (see Figure 7).

To investigate the developmental trends of the main effects, a mixed‐effects regression was computed on 11 regions of interest (ROI): the vmPFC and PCC for the choice‐probability signal, the left and right striatum and insula for the SU‐ and DU‐PE signal, and the MFG for the SU‐PE signal (see Figure 7 and Table S17 for a complete list of main effect results). The SU‐PE signal in the left insula was significantly increasing over time (*β* = 0.12*, z* = 2.05, *p* = 0.041) and the SU‐PE‐signal in the left striatum was significantly decreasing over time (*β* = −0.35*, z* = −4.97, *p* < 0.001).

#### 3.2.2 Association of Drinking and Neural Correlates of Choice Probability and PEs

**FIGURE 8:**
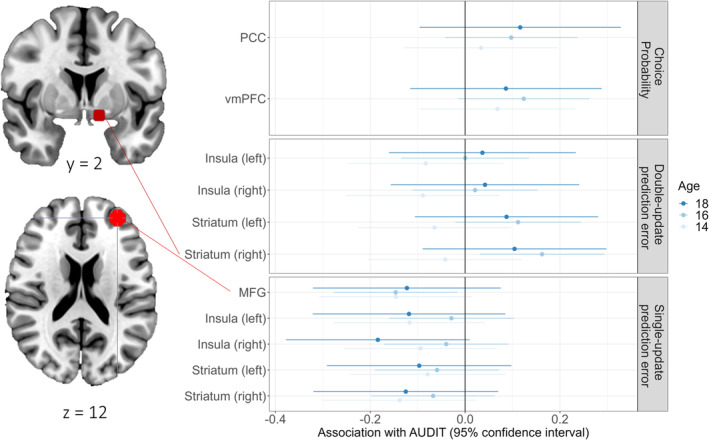
95% confidence intervals of the LGCM estimates for neural correlates of probabilistic reversal learning (y‐axis) as time‐varying predictors of AUDIT total score (Alcohol Use Disorder Identification Test) separated by age and the respective contrast of interest. The single‐update prediction error in the MFG region of interest (ROI, red) was negatively associated with AUDIT scores. The double‐update prediction error in the right striatum ROI (dark red) was positively associated with AUDIT scores. MFG: medial frontal gyrus; PCC: posterior cingulate cortex; vmPFC: ventromedial prefrontal cortex.

After establishing the main and developmental effects, we investigated whether neural processing during the PReL task could provide insights into the association between PReL and alcohol use. To this end, we computed latent growth curve models (LGCMs) with intercept and slope for the AUDIT scores, using the signal in the above defined ROIs as a time‐varying predictor to examine cross‐sectional associations. We found two significant associations between brain signals and AUDIT at age 16: SU‐PE signal in MFG (*β* = −0.179, *p* = 0.026, CI = [−0.336, −0.022]) and DU‐PE signal in the right striatum (*β* = 0.148, *p* = 0.017, CI = [0.026, 0.270], see Figure 8). That is, participants who drink more at age 16 recruit less frontal activity during feedback processing for the chosen option, whereas their striatal activity is higher during feedback processing for the unchosen option. Please refer to Chapter 4.6 in the supporting information for the complete LGCM results.

Additionally, we computed correlation between the cumulative alcohol consumption and difference scores of ROI signals to estimate the longitudinal effect of alcohol on PReL processing. Interestingly, we see positive correlations of cumulative alcohol consumption with differences in PE‐SU‐signal (right striatum: *r* = 0.177, *p* = 0.035; right insula: *r* = 0.186, *p* = 0.026) but negative correlations with differences in PE‐DU‐signal (right striatum: *r* = −0.253, *p* = 0.002; striatum left: *r* = −0.193, *p* = 0.021; left insula: *r* = −0.229, *p* = 0.006; see Table S31 for complete correlation results). It can be concluded that alcohol consumption might slow down the decrease in striatal activation and promote an increase in insular activation during the updating of the chosen option. Conversely, cumulative alcohol consumption could promote the decrease of striatal and insular activation during the update of the unchosen option.

#### 3.2.3 fMRI Reliability

In previous studies, we have shown that robust fMRI main effects on the group level are not necessarily a result of reliable signal on the individual level [38]. Hence, we computed ICCs for the extracted ROI signals used in the LGCM analyses (see Table S33). The MFG ROI from the SU‐PE contrast was the only ROI with an ICC higher than 0.2, longitudinally and averaged across split‐half reliability of each wave. Especially the subcortical ROIs (insula and striatum) showed very low ICCs, which is in line with previous literature (e.g., [39]). Notably, the split‐half ICCs varied a lot between waves, which itself is an indicator for the low reliability of the data.

## 4 Discussion

This longitudinal fMRI study investigated the association between PReL and alcohol use in adolescents aged 14 to 18. During the study, alcohol use among participants increased, but remained predominantly low‐level. We observed reduced medial frontal activity associated with updating the chosen option and increased striatal activity associated with updating the unchosen option in those who drink more. In contrast to our expectations, no associations were found between drinking and performance measures, probabilities for repeating choices, or computational parameters.

### 4.1 PReL and Drinking

By means of fMRI, we showed that lower medial frontal activity evoked by the SU‐PE was associated with more drinking at age 16. The results support the notion that increased (or addicted) alcohol use is associated with diminished frontal activity or disrupted frontal‐striatal coupling [6, 7, 14]. In a similar vein, the DU‐PE‐signal in right striatum was positively associated with drinking at age 16. However, PE‐signal in the ventral striatum did not differ between patients with alcohol dependence/AUD and controls [6, 7, 14]. The analysis of cumulative alcohol consumption revealed that alcohol use might have inverse effects on the development of neural correlates of SU and DU. Most prominently, we see that the found decrease in striatal activation during the update of the chosen option might be slowed down in those who drink more. In sum, associations between neuronal correlates of PReL and drinking were only found cross‐sectionally at the age of 16, while we found indications of a longitudinal effect of alcohol exposure on the development of feedback processing. These associations were small and might become more relevant also for the actual behaviour with higher alcohol use. However, neither behavioural nor computational PReL parameters were associated with drinking in our sample. Assuming that low‐level alcohol consumption does not lead to many negative consequences, the processing of such and resulting actions might be rather relevant for higher levels of alcohol use, as shown in AUD patients (e.g., [7]). The rarity of high‐level drinking in our sample may explain that we did not find any behavioural association. However, caution is warranted because the reliability of accuracy is very low which limits its potential to identify associations with other variables (see Table S3; [41]). The reliability of computational parameters was higher compared to the behavioural outcomes, which makes them more suitable for research of inter‐ and intraindividual differences. Nevertheless, we could not find evidence for an association of computational parameters and drinking, which is in line with one patient study [14]. Other studies, however, found differences in learning rates or DU‐weighted learning especially from negative feedback between patients and controls [7, 42]. Our data did not permit us to reproduce these specific findings regarding punishment learning (rate), as our computational model does not differentiate the learning rate for wins and losses.

### 4.2 Development of PReL During Adolescence

Measured by an adaptive PReL task, participants achieved more contingency changes over time by increasing their stay probability, i.e., more consistent behaviour. Computational modelling revealed that participants learned faster and became more sensitive to wins and less sensitive to losses over time. According to simulation results, increased learning rate and decreased sensitivity to losses can be seen as an adaptive progress since they lead to higher accuracy (see Figure S6). Our results were mostly in line with a cross‐sequential study of PReL in 12‐ to 45‐year‐old participants that reported better PReL in older participants attributed to a relatively higher sensitivity to wins than to losses, resulting in fewer switches after losses [11]. This evidence, taken together with the finding that adolescents learn faster from negative feedback than adults [16], suggests that one aspect of RL development is the shift of the feedback focus. Specifically, the older the participants, the less they seem to focus on negative feedback. Using fMRI, significant change could only be identified in the SU‐PE signal, which contrasts with studies finding no age effect on PE signals [11, 19]. Signal in the left striatum and insula developed in an inverse manner, with striatal signal decreasing significantly and the insular signal increasing significantly. However, caution is warranted while interpreting the developmental trajectories of the subcortical signals given their low reliability discussed below.

### 4.3 Limitations

First, the low reliability of subcortical signals in our data warrants caution in interpreting developmental trajectories and associations with drinking. Although we would not anticipate high reliability given the expected change over time, we still believe that a near‐zero reliability hinders the interpretation of developmental trajectories. The community started to collect evidence about approaches that may increase reliability, including increasing between‐subject variability, multilevel models, considerations for fMRI designs such as low reliability of difference and parametric contrasts, and potential focus on cortical compared to subcortical ROIs [31, 43, 44, 45].

Second, our implementation of the PReL task is notably challenging, as indicated by accuracies hovering around 60% (with 50% as chance level), and it becomes more difficult as participants perform better. Recent studies have adopted nonadaptive task versions that are easier for participants, allowing for better exploration of inter‐ and intraindividual differences [7, 11, 31].

Third, we cannot clearly separate aging and training effects here. In fact, simulations have shown that a higher learning rate leads to higher accuracy. Thus, the developmental increase in the learning rate could reflect the optimization of task behaviour. However, it is questionable whether participants could adapt their behaviour based on experiences from 2 years ago, as this is the interval between acquisition waves.

Fourth, as noted above, our participants engaged in low‐level drinking during the course of the study. However, the alcohol consumption of our participants is consistent with a representative sample of German adolescents and young adults [46]. Additionally, our retrospective assessments capture patterns over multiple weeks, but they may not fully reflect short‐term fluctuations in alcohol use, such as binge drinking episodes. This limitation has been acknowledged in similar studies and represents a general challenge in longitudinal alcohol research [28]. Future studies might benefit from using ecological momentary assessment to target these short‐term fluctuations as has been done in recent studies, e.g., linking alcohol consumption and lockdown measures due to COVID‐19 [47].

Finally, it is necessary to consider the issue of multiple comparisons when evaluating the effect sizes, given the number of computed tests, especially correlations and LGCMs.

### 4.4 Conclusions

This study examined the association between PReL behaviour and low‐level drinking in adolescents. The results suggest that adolescents become more adaptive learners over time and, not surprisingly, increase alcohol drinking. However, these trajectories appear to progress independently. Importantly, we found that less recruitment of frontal networks during feedback processing, which might reflect less cognitive control, was associated with more drinking. Considering the small effect sizes, we conclude that PReL, specifically the processing and reaction to negative feedback, may be more relevant for higher levels of drinking or even the escalation of alcohol use towards heavy drinking or AUD. To test this hypothesis, future studies need larger samples and the consideration of drop‐out of potentially more relevant, i.e., more drinking, participants. Finally, efforts should be invested in developing and using suitable paradigms and methods for longitudinal (fMRI) analyses, e.g., by increasing between‐subject variance in the data, using multilevel approaches, and careful definition of contrasts and regions of interest.

Author Contributions

Juliane H. Fröhner (J.H.F.) conducted the investigation, curated the data, prepared the original draft and visualization, performed formal analysis, and managed the project. Maria Waltmann (M.W.) contributed to methodology, provided resources, and participated in writing review and editing. Andrea M. F. Reiter (A.M.F.R.) contributed to resources and assisted with writing review and editing. Anja Kräplin (A.K.) assisted formal analysis, contributed to visualization, and participated in writing review and editing. Michael N. Smolka (M.N.S.) was responsible for conceptualization, contributed to methodology, supervised the project, secured funding, and participated in writing review and editing. All authors read and approved the final manuscript.

## Author Contributions

**Juliane H. Fröhner (J.H.F.)** conducted the investigation, curated the data, prepared the original draft and visualization, performed formal analysis, and managed the project.**Maria Waltmann (M.W.)** contributed to methodology, provided resources, and participated in writing review and editing.**Andrea M. F. Reiter (A.M.F.R.)** contributed to resources and assisted with writing review and editing.**Anja Kräplin (A.K.)** assisted formal analysis, contributed to visualization, and participated in writing review and editing.**Michael N. Smolka (M.N.S.)** was responsible for conceptualization, contributed to methodology, supervised the project, secured funding, and participated in writing review and editing. All authors read and approved the final manuscript.

## Ethics Statement

Subjects participated in the study after giving written informed consent and for those who were under 18 years old, at least one parent had to additionally agree to their participation by signing the consent form. The study was approved by the local research ethics committee (Ethics Committee of the Technische Universität Dresden) and conducted in accordance with the Declaration of Helsinki.

## Conflicts of Interest

The authors declare no conflicts of interest.

## Supporting information


**Table S1:** Mean and standard deviation (in brackets) for behavioural estimates
**Table S2:** Pearson’s correlation (r) between performance indices
**Table S3:** Mean and variance for the computed empirical Gaussian priors.
**Table S4:** Bayesian information criterion (BIC) for joint model estimation and separate estimation for each age group. The winning model over all estimations is highlighted in orange.
**Table S5:** Correlation of refitted parameters from simulated behaviour.
**Table S6:** Pearson’s correlation and mean (M) and standard deviation (SD) for computational parameters.
**Table S7:** Pearson’s correlation r between computational parameters and behavioural indices
**Table S8:** Pearson’s correlation r between cumulative alcohol consumption and difference scores (age 18 – age 14) of computational parameters and behavioural indices
**Table S9:** Model fit for each latent growth curve model including fMRI data
**Table S10:** Model fits for behavioural and computational parameters for models with and without (w/o) covariates
**Table S11:** Latent growth curve model estimates for estimated accuracy as time‐varying covariate of drinking measured by AUDIT
**Table S12:** Latent growth curve model estimates for estimated probability to stay after a win as time‐varying covariate of drinking measured by AUDIT
**Table S13:** Latent growth curve model estimates for estimated probability to stay after a loss as time‐varying covariate of drinking measured by AUDIT
**Table S14:** Latent growth curve model estimates for learning rate as time‐varying covariate of drinking measured by AUDIT
**Table S15:** Latent growth curve model estimates for reinforcement sensitivity (rho) for wins as time‐varying covariate of drinking measured by AUDIT
**Table S16:** Latent growth curve model estimates for reinforcement sensitivity (rho) for losses as time‐varying covariate of drinking measured by AUDIT
**Table S17:** Main effects over all sessions (p_FWE‐corrected_ < 0.05, cluster size > 50) separated by contrast. Regions of interest (ROI) highlighted in blue. Light blue:Cluster in the left hippocampus included the left striatal ROI (MNI coordinates [−12,5,‐11], t = 7.12, p_FWE‐corrected_ < 0.001). The right striatal ROI was slightly smaller than 50 voxels (MNI coordinates [12,5,‐11], t = 6.18, p_FWE‐corrected_ < 0.001).
**Table S18:** Correlation of signal in regions of interest with estimated behaviour
**Table S19:** Mixed effects regions for regions of interest in main effects of choice probability and prediction errors
**Table S20:** Main effect of AUDIT over all sessions (p_FWE‐corrected_ < 0.05).
**Table S21:** Latent growth curve model estimates for choice probability signal in ventromedial prefrontal cortex as time‐varying covariate of drinking
**Table S22:** Latent growth curve model estimates for choice probability signal in posterior cingulate cortex as time‐varying covariate of drinking
**Table S23:** Latent growth curve model estimates for single‐update prediction error in right striatum as time‐varying covariate of drinking
**Table S24:** Latent growth curve model estimates for single‐update prediction error in left striatum as time‐varying covariate of drinking
**Table S25:** Latent growth curve model estimates for single‐update prediction error in right insula as time‐varying covariate of drinking
**Table S26:** Latent growth curve model estimates for single‐update prediction error in left insula as time‐varying covariate of drinking
**Table S27:** Latent growth curve model estimates for single‐update prediction error in MFG as time‐varying covariate of drinking
**Table S28:** Latent growth curve model estimates for double‐update prediction error in right striatum as time‐varying covariate of drinking
**Table S29:** Latent growth curve model estimates for double‐update prediction error in left striatum as time‐varying covariate of drinking
**Table S30:** Latent growth curve model estimates for double‐update prediction error in right insula as time‐varying covariate of drinking
**Table S31:** Latent growth curve model estimates for double‐update prediction error in left insula as time‐varying covariate of drinking
**Table S32:** Pearson’s correlation r between cumulative alcohol consumption and difference scores (age 18 – age 14) of ROI signals
**Table S33:** Intraclass coefficient (ICC) for signals extracted from regions of interest for the main effects of respective contrasts
**Figure S1:** Variance components of accuracy as example. Mixed‐effects models increase reliability by reducing error variance and increasing within‐subject variance.
**Figure S2:** Reliability of parameters estimated by mixed‐effects models from separate modelling (grey) and joint modelling (colours).
**Figure S3:** Normal distribution with mean and variance of the computed priors per age group and computational parameters.
**Figure S4:** Model comparison via the integrated Bayesian information criterion (iBIC; 4) for the joint estimation of all sessions. On the left: Absolute iBIC. On the right: Difference between iBIC of model and iBIC of best fitting model. Choice sensitivity models are depicted in blue, reinforcement sensitivity models are depicted in yellow. The star marks the winning model.
**Figure S5:** Moderate to high correlation between original and simulated data (100 simulations per 143 parameter combinations).
**Figure S6:** Simulated accuracy with computational parameters within the parameter range of the data and separated by number of system changes that occurred.
**Figure S7:** Simulated accuracy dependent on probability to stay after a loss, separated by number of contingency changes that occurred. The blue line represents the overall effect.
**Figure S8:** Regions of interest defined by theory‐driven selection of main‐effect peaks. Yellow: Choice probability signal in ventromedial prefrontal cortex (vmPFC) and posterior cingulate cortex (PCC). Blue: Single‐update prediction error in striatum, insula and medial frontal gyrus (MFG). Turquoise: Double‐update prediction error in striatum and insula.
**Figure S9:** Development of double‐update (DU) prediction error signal. Two top panels: negative association of prediction error and insula signal. Two bottom panels: positive association of prediction error and striatal signal.
**Figure S10:** Development of single‐update (SU) prediction error signal. Three top panels: negative association of prediction error and insula and medial frontal gyrus (MFG) signal. Two bottom panels: positive association of prediction error and striatal signal. Striatal signal (left) is significantly decreasing and insular signal (left) is significantly increasing (see Table S18).
**Figure S11:** Development of choice‐probability signal in ventromedial prefrontal cortex (vmPFC) and posterior cingulate cortex (PCC).
**Figure S12:** Development of single‐update prediction error signal that was negatively associated with drinking.

## Data Availability

The data that support the findings of this study are available from the corresponding author upon reasonable request.
