# Supplementary material for: Relevance of Probabilistic Reversal Learning for Adolescent Drinking Trajectories
Source: Addict Biol. 2025 Mar 6;30(3):e70026. doi: 10.1111/adb.70026 (PMC11884864; doi:10.1111/adb.70026)
Supplement: Supplementary file 1 — Table S1: Mean and standard deviation (in brackets) for behavioural estimates Table S2: Pearson’s correlation (r) between performance indices Table S3: Mean and variance for the computed empirical Gaussian priors. Table S4: Bayesian information criterion (BIC) for joint model estimation and separate estimation for each age group. The winning model over all estimations is highlighted in orange. Table S5: Correlation of refitted parameters from simulated behaviour. Table S6: Pearson’s correlation and mean (M) and standard deviation (SD) for computational parameters. Table S7: Pearson’s correlation r between computational parameters and behavioural indices Table S8: Pearson’s correlation r between cumulative alcohol consumption and difference scores (age 18 – age 14) of computational parameters and behavioural indices Table S9: Model fit for each latent growth curve model including fMRI data Table S10: Model fits for behavioural and computational parameters for models with and without (w/o) covariates Table S11: Latent growth curve model estimates for estimated accuracy as time‐varying covariate of drinking measured by AUDIT Table S12: Latent growth curve model estimates for estimated probability to stay after a win as time‐varying covariate of drinking measured by AUDIT Table S13: Latent growth curve model estimates for estimated probability to stay after a loss as time‐varying covariate of drinking measured by AUDIT Table S14: Latent growth curve model estimates for learning rate as time‐varying covariate of drinking measured by AUDIT Table S15: Latent growth curve model estimates for reinforcement sensitivity (rho) for wins as time‐varying covariate of drinking measured by AUDIT Table S16: Latent growth curve model estimates for reinforcement sensitivity (rho) for losses as time‐varying covariate of drinking measured by AUDIT Table S17: Main effects over all sessions (pFWE‐corrected < 0.05, cluster size > 50) separated by contrast. Regions of interest (ROI) highligh [file ADB-30-e70026-s001.docx]

# Supplement for manuscript “Relevance of probabilistic reversal learning for adolescent drinking trajectories”

# Behavioral data

## Descriptive statistics of behavioral estimates

Table S1: Mean and standard deviation (in brackets) for behavioral estimates

| Age | 14 | 16 | 18 |
| --- | --- | --- | --- |
| Obtained reward | 3.64 (1.43) | 3.86 (1.53) | 3.81 (1.51) |
| Number of contingency changes | 6.48 (2.331) | 6.87 (2.334) | 7.58 (2.262) |
| Accuracy* | 0.59 (0.04) | 0.61 (0.03) | 0.61 (0.04) |
| Probability to stay after win* | 0.93 (0.600) | 0.95 (0.058) | 0.95 (0.065) |
| Probability to stay after loss* | 0.54 (0.124) | 0.55 (0.123) | 0.57 (0.100) |
| **Note:* *estimated from mixed-effects models | | | |
|  | | | |

## Correlation of behavioral performance indices

The PReL task under investigation was challenging and adaptive. Consequently, we chose to evaluate performance using three distinct measures that showed low to moderate correlations (see Table S2):

- Accuracy, which represents the percentage of correct responses irrespective of feedback.
- Number of contingency changes, which counts the switches in contingencies after four consecutive correct responses and serves as an indicator of both task performance and difficulty.
- Obtained reward, which refers to performance-related feedback that participants relied on to guide their behavior.

## Reliability of behavioral data

According to Waltmann, Schlagenhauf (1), the reliability of PReL data can be improved by predicting behavioral estimates using mixed-effects models. They argue that these models enhance reliability by reducing error variance and increasing within-subject variance. Figure S1 illustrates this effect on accuracy. Furthermore, Figure S2 shows that *joint* modeling of all completed sessions is particularly beneficial for improving reliability, exemplary for accuracy and stay probabilities after losses. However, the intraclass correlation coefficients (ICC) for absolute agreement remained very low for accuracy (ICC overall sessions= -0.011, CI [-0.098,0.089], *p* = 0.586).

Table S2: Pearson’s correlation (r) between performance indices

| Age |  | Variable | 1 | 2 | 3 | 4 | 5 | 6 | 7 | 8 | |
| --- | --- | --- | --- | --- | --- | --- | --- | --- | --- | --- | --- |
| 14 |  |  |  |  |  |  |  |  |  |  | |
|  | 1 | Accuracy |  |  |  |  |  |  |  |  | |
|  |  |  |  |  |  |  |  |  |  |  | |
|  | 2 | Cont | .30** |  |  |  |  |  |  |  | |
|  |  |  | [.15, .44] |  |  |  |  |  |  |  | |
|  | 3 | Reward | .67** | .22** |  |  |  |  |  |  | |
|  |  |  | [.57, .75] | [.06, .37] |  |  |  |  |  |  | |
| 16 | 4 | Accuracy | .06 | -.15 | .07 |  |  |  |  |  | |
|  |  |  | [-.11, .22] | [-.31, .01] | [-.09, .23] |  |  |  |  |  | |
|  | 5 | Cont | .01 | -.00 | .07 | .40** |  |  |  |  | |
|  |  |  | [-.15, .18] | [-.17, .16] | [-.10, .23] | [.25, .53] |  |  |  |  | |
|  | 6 | Reward | .05 | -.12 | -.01 | .60** | .30** |  |  |  | |
|  |  |  | [-.11, .22] | [-.28, .05] | [-.18, .15] | [.48, .69] | [.14, .44] |  |  |  | |
| 18 | 7 | Accuracy | -.05 | -.08 | .00 | -.11 | -.12 | .03 |  |  | |
|  |  |  | [-.22, .11] | [-.24, .09] | [-.16, .17] | [-.27, .05] | [-.28, .05] | [-.13, .20] |  |  | |
|  | 8 | Cont | .11 | .08 | .14 | -.11 | .04 | -.07 | .39** |  | |
|  |  |  | [-.05, .27] | [-.08, .24] | [-.03, .30] | [-.27, .06] | [-.12, .21] | [-.23, .09] | [.24, .52] |  | |
|  | 9 | Reward | .01 | -.05 | .15 | -.02 | -.02 | .03 | .57** | .32** | |
|  |  |  | [-.15, .18] | [-.22, .11] | [-.02, .30] | [-.18, .14] | [-.19, .14] | [-.14, .19] | [.45, .67] | [.17, .46] | |
| *Note:* Grey = within session. Cont = Number of contingency changes, Reward = obtained reward. | | | | | | | | | | |  |


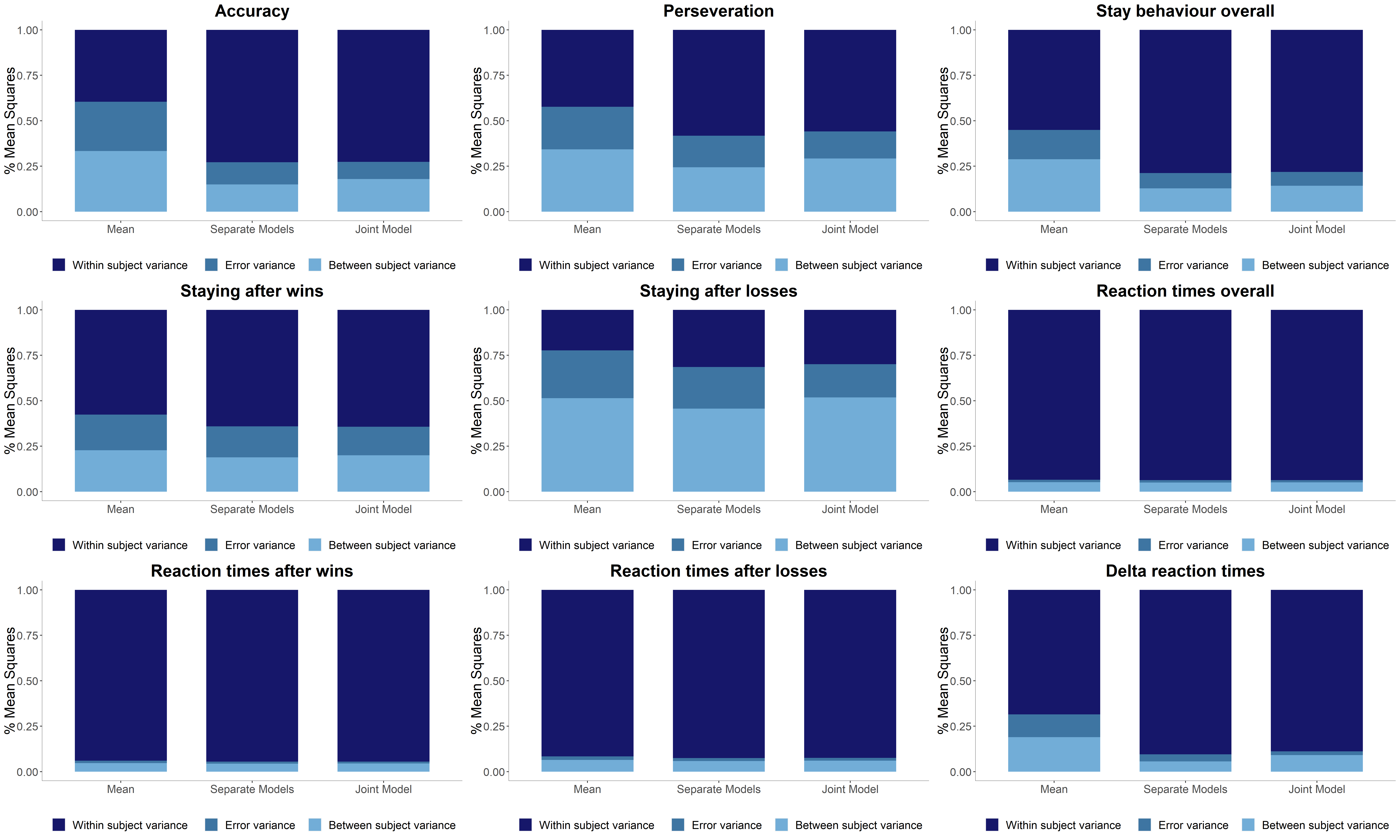


Figure S1: Variance components of accuracy as example. Mixed-effects models increase reliability by reducing error variance and increasing within-subject variance.


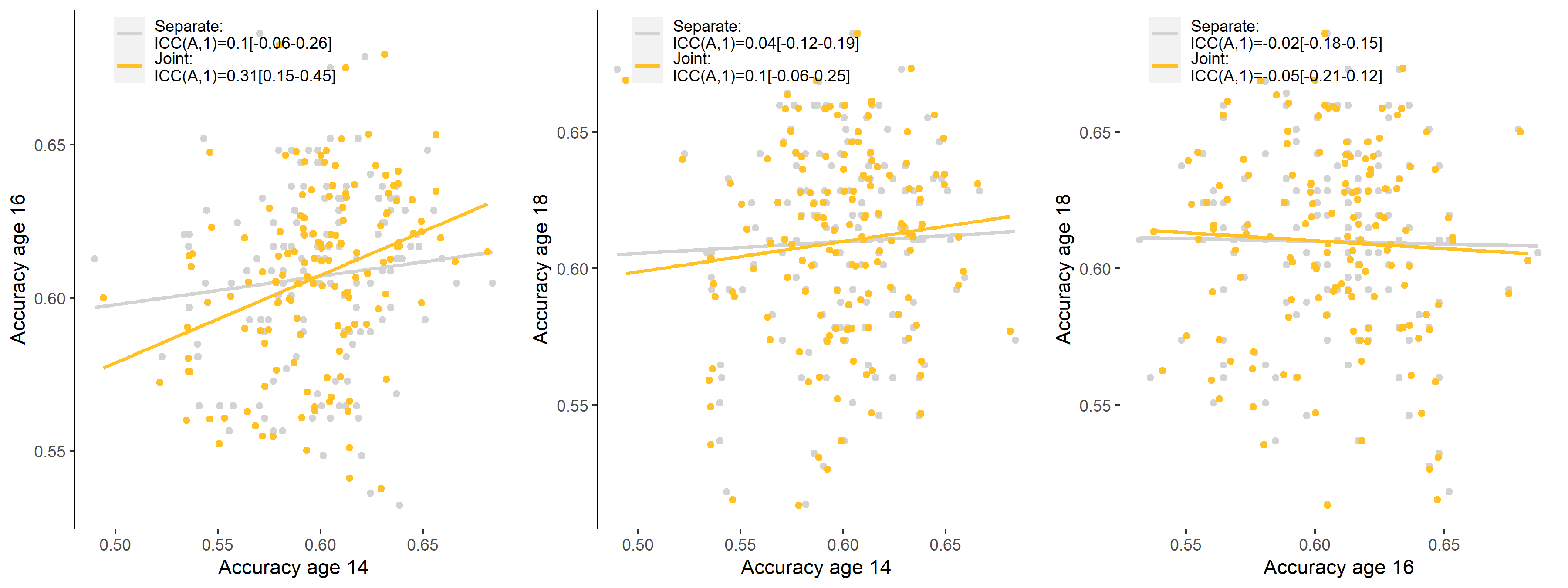

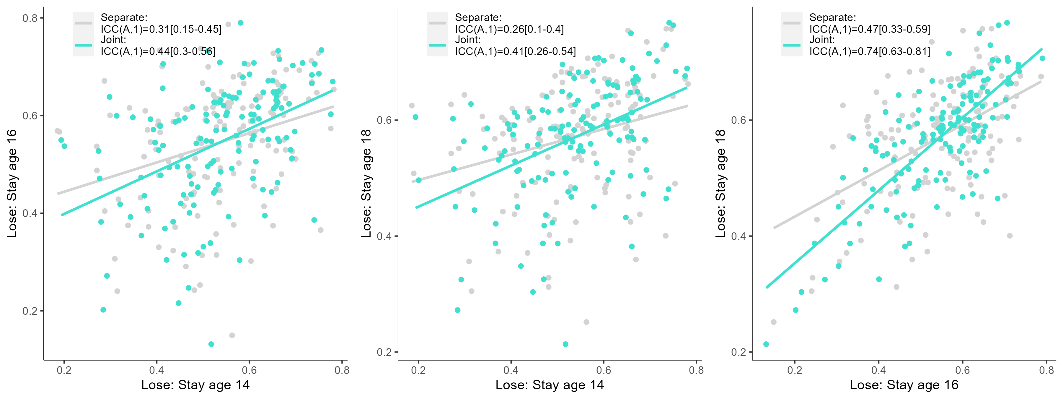


Figure S2: Reliability of parameters estimated by mixed-effects models from separate modelling (grey) and joint modelling (colors).

# Computational modelling

Following the approach of Waltmann, Schlagenhauf (1), we compared choice- and reinforcement sensitivity models. For the choice-sensitivity models, the observational model defined the choice probability (*p*) for action (a) i using the softmax function equation (1), where the parameter *β* regulates the steepness of the function, affecting choice stochasticity. Smaller *β* values result in more stochastic and explorative choices, indicating less reliance on value differences for action selection.

| $p\left( a_{i} \right)=\frac{exp(\beta Q_{a_{i}})}{\sum_{j=1}^{K} exp(\beta Q_{a_{i}})}$ | (1) |
| --- | --- |

For the reinforcement-sensitivity models, the parameter *β* is dropped for the observational model and the parameter *ρ* is added to the perceptual model (see (2)). Reinforcement sensitivity *ρ* influences choice stochasticity by determining the maximum difference between expected values, thus providing a lower bound on choice stochasticity. Here, $Q_{a,t}$ is the expected value of an action (a) in trial t. The expected value of the next trial $Q_{a,t+1}$ is an update of the previous expected value by a PE (in brackets), which represents the difference of the actual feedback *r* and the previous expected value $Q_{a,t}$. The parameter *ρ* determines the maximum difference between expected value, influencing the stochasticity of decision-making after receiving feedback (the probability of win – stay, loss – switch).

| $Q_{a,t+1}=Q_{a,t}+\alpha(\rho r-Q_{a,t}$*)* | (2) |
| --- | --- |

The learning rate *α*, which signifies the weighting of recent feedback in comparison to integrated feedback from previous trials, determines how much recent trials contribute to the learning process (i.e., 1 means only considering the most recent trial).

For both model families, we estimated the learning rate *α* and the choice sensitivity *β*, or the reinforcement sensitivity *ρ*, over all trials or separately for wins and losses. In addition, we differentiated between single-update (SU) and double-update (DU) models. Given the anti-correlated structure of the task, participants can learn not only about the chosen stimulus (SU), but also about the unchosen stimulus (DU). Finally, participants may vary in the extent to which they update the unchosen value, which is represented as parameter *κ* in the individual DU models. The parameter *κ* is implemented as a weight for the learning rate *α* in the update equation for the value of the unchosen option. In sum, we compared twelve models for each model family, so 24 models in total.

We used MATLAB 2020b and the emfit toolbox to estimate parameters by maximum a posteriori estimation with empirical priors (2). We used empirical Gaussian priors on our parameters (*p(θ| μ, σ)*), inferred from the multivariate distribution of the estimates across subjects in an expectation maximization procedure (see Table S4; 3, 4).

Table S3: Mean and variance for the computed empirical Gaussian priors.

| Parameter | Age | Mean | Variance |
| --- | --- | --- | --- |
| Rho_win | 14 | 2.365 | 1.203 |
| Rho_loss | 14 | -0.768 | 0.250 |
| Rho_win | 16 | 2.611 | 1.378 |
| Rho_loss | 16 | -0.770 | 0.250 |
| Rho_win | 18 | 2.617 | 1.205 |
| Rho_loss | 18 | -0.658 | 0.225 |
| Alpha | 14 | 0.461 | 0.439 |
| Alpha | 16 | 0.526 | 0.232 |
| Alpha | 18 | 0.620 | 0.295 |


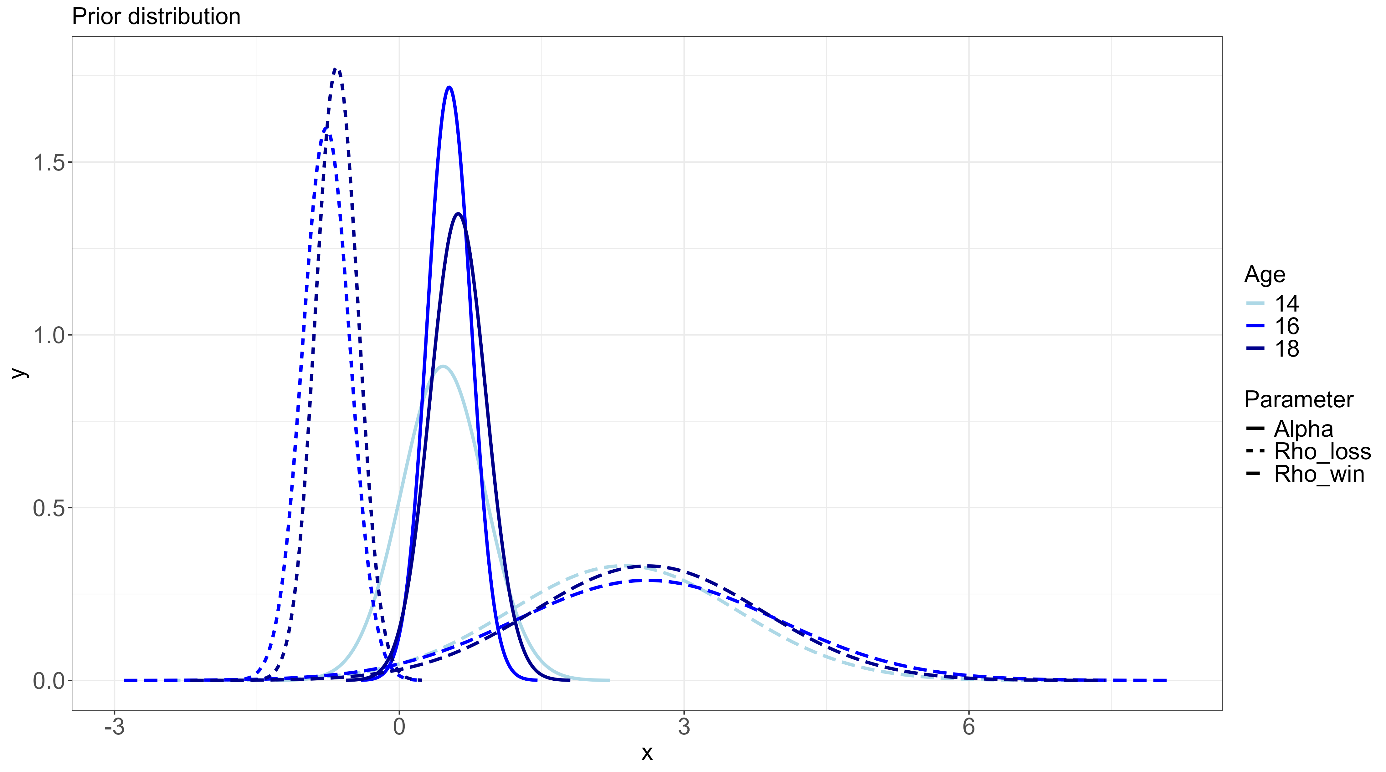


Figure S3: Normal distribution with mean and variance of the computed priors per age group and computational parameters.

## Model comparison

We compared models according to their integrated Bayesian information criterion (BIC; 4). The double-update model with two reinforcement sensitivities *ρ* and one learning rate *α* fit the best in the joint estimation (DU-2𝜌𝛼-model; see Figure S4 and Table S4). The winning model was the same when sessions were fit separately. Since it was shown that reliability of parameters benefits from joint modelling, we continued the analyses with these parameters (1).


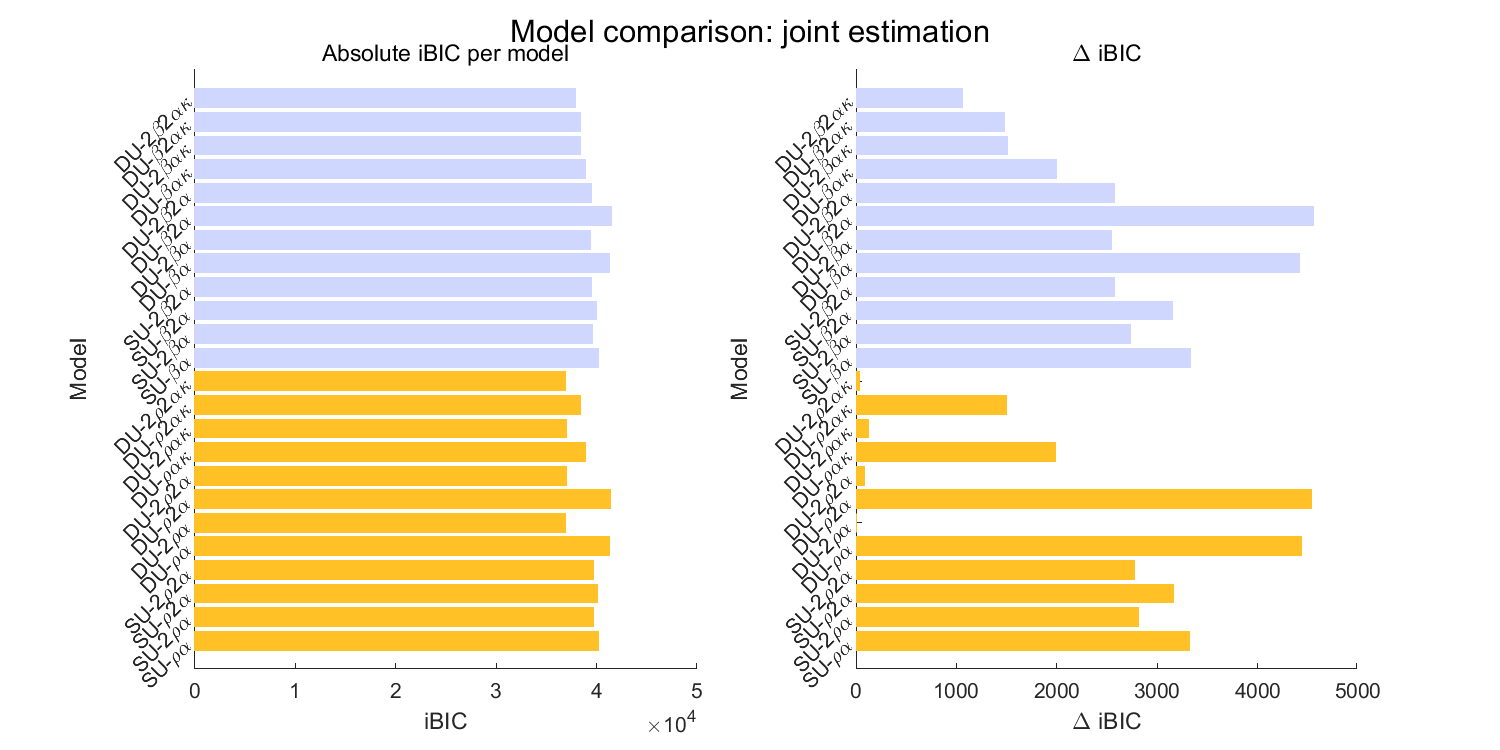


Figure S4: Model comparison via the integrated Bayesian information criterion (iBIC; 4) for the joint estimation of all sessions. On the left: Absolute iBIC. On the right: Difference between iBIC of model and iBIC of best fitting model. Choice sensitivity models are depicted in blue, reinforcement sensitivity models are depicted in yellow. The star marks the winning model.

Table S4: Bayesian information criterion (BIC) for joint model estimation and separate estimation for each age group. The winning model over all estimations is highlighted in orange.

| **Estimation** | Joint | | Age 14 | | Age 16 | | Age 18 | |  |
| --- | --- | --- | --- | --- | --- | --- | --- | --- | --- |
| **Model** | BIC | DeltaBIC | BIC | DeltaBIC | BIC | DeltaBIC | BIC | DeltaBIC |  |
| *Reinforcement sensitvity models* | | | | | | | | |  |
| SUρα | 40205.9 | 3325.3 | 16058.7 | 1678.3 | 17924.9 | 2718.4 | 19234.8 | 3666.1 |  |
| SU2ρα | 39697.6 | 2816.9 | 15889.0 | 1508.6 | 17603.5 | 2396.9 | 18961.6 | 3392.9 |  |
| SUρ2α | 40042.2 | 3161.5 | 15971.8 | 1591.5 | 17871.5 | 2665.0 | 19154.3 | 3585.6 |  |
| SU2ρ2α | 39658.0 | 2777.3 | 15856.2 | 1475.8 | 17543.2 | 2336.7 | 18874.4 | 3305.7 |  |
| DUρα | 41317.1 | 4436.5 | 16376.1 | 1995.7 | 18422.0 | 3215.5 | 19484.5 | 3915.9 |  |
| DU2ρα | 36880.6 | 0.0 | 14380.4 | 0.0 | 15206.5 | 0.0 | 15568.7 | 0.0 |  |
| DUρ2α | 41419.2 | 4538.6 | 16230.9 | 1850.6 | 18445.2 | 3238.7 | 19530.7 | 3962.1 |  |
| DU2ρ2α | 36962.5 | 81.8 | 14424.2 | 43.8 | 15223.2 | 16.6 | 15588.2 | 19.5 |  |
| DUρακ | 38863.0 | 1982.3 | 15705.8 | 1325.5 | 17664.3 | 2457.7 | 19000.6 | 3431.9 |  |
| DU2ρακ | 37006.8 | 126.2 | 14413.9 | 33.5 | 15231.5 | 25.0 | 15596.5 | 27.9 |  |
| DUρ2ακ | 38381.3 | 1500.7 | 15476.4 | 1096.0 | 17624.1 | 2417.5 | 18920.8 | 3352.1 |  |
| DU2ρ2ακ | 36913.4 | 32.8 | 14414.3 | 34.0 | 15232.4 | 25.9 | 15587.1 | 18.4 |  |
| *Choice sensitivity models* | | | | | | | | |  |
| SUβα | 40208.4 | 3327.8 | 16054.0 | 1673.6 | 17924.9 | 2718.4 | 19229.6 | 3660.9 |  |
| SU2βα | 39615.5 | 2734.9 | 15479.6 | 1099.3 | 17085.0 | 1878.4 | 18072.9 | 2504.2 |  |
| SUβ2α | 40031.8 | 3151.1 | 15968.1 | 1587.7 | 17867.1 | 2660.6 | 19154.7 | 3586.0 |  |
| SU2β2α | 39452.3 | 2571.7 | 15427.7 | 1047.4 | 17104.2 | 1897.7 | 18031.6 | 2462.9 |  |
| DUβα | 41300.6 | 4420.0 | 16383.0 | 2002.6 | 18422.1 | 3215.5 | 19482.5 | 3913.8 |  |
| DU2βα | 39420.5 | 2539.9 | 15168.9 | 788.5 | 16349.4 | 1142.8 | 16942.5 | 1373.8 |  |
| DUβ2α | 41434.6 | 4554.0 | 16224.5 | 1844.1 | 18456.4 | 3249.9 | 19535.7 | 3967.0 |  |
| DU2β2α | 39457.6 | 2576.9 | 15157.6 | 777.2 | 16367.2 | 1160.7 | 16979.0 | 1410.3 |  |
| DUβακ | 38878.4 | 1997.7 | 15703.2 | 1322.8 | 17664.2 | 2457.7 | 18994.4 | 3425.7 |  |
| DU2βακ | 38385.7 | 1505.0 | 15016.3 | 635.9 | 16201.8 | 995.3 | 16868.8 | 1300.1 |  |
| DUβ2ακ | 38360.9 | 1480.3 | 15482.5 | 1102.1 | 17623.4 | 2416.9 | 18933.1 | 3364.4 |  |
| DU2β2ακ | 37944.6 | 1064.0 | 14835.8 | 455.4 | 16200.1 | 993.6 | 16844.3 | 1275.6 |  |
| *Note*: BIC =Bayesian Information Criterion; DeltaBIC = Difference between BIC of model and lowest BIC | | | | | | | | |  |
|  |  |  |  |  |  |  |  |  |  |

## Parameter recoverability

To further check quality of the model fit for our data, we extracted the parameters for the 143 subjects and created 100 simulated data sets. We found moderate to high correlations between simulated and original behavior (0.61 ≤ *r* ≤ 0.98; see Figure S5). Afterwards, we refitted the double-update-2𝜌𝛼-model to the simulated data. The estimated parameters correlated highly with the underlying true values (0.90 ≤ *r* ≤ 0.96; see Table S5).


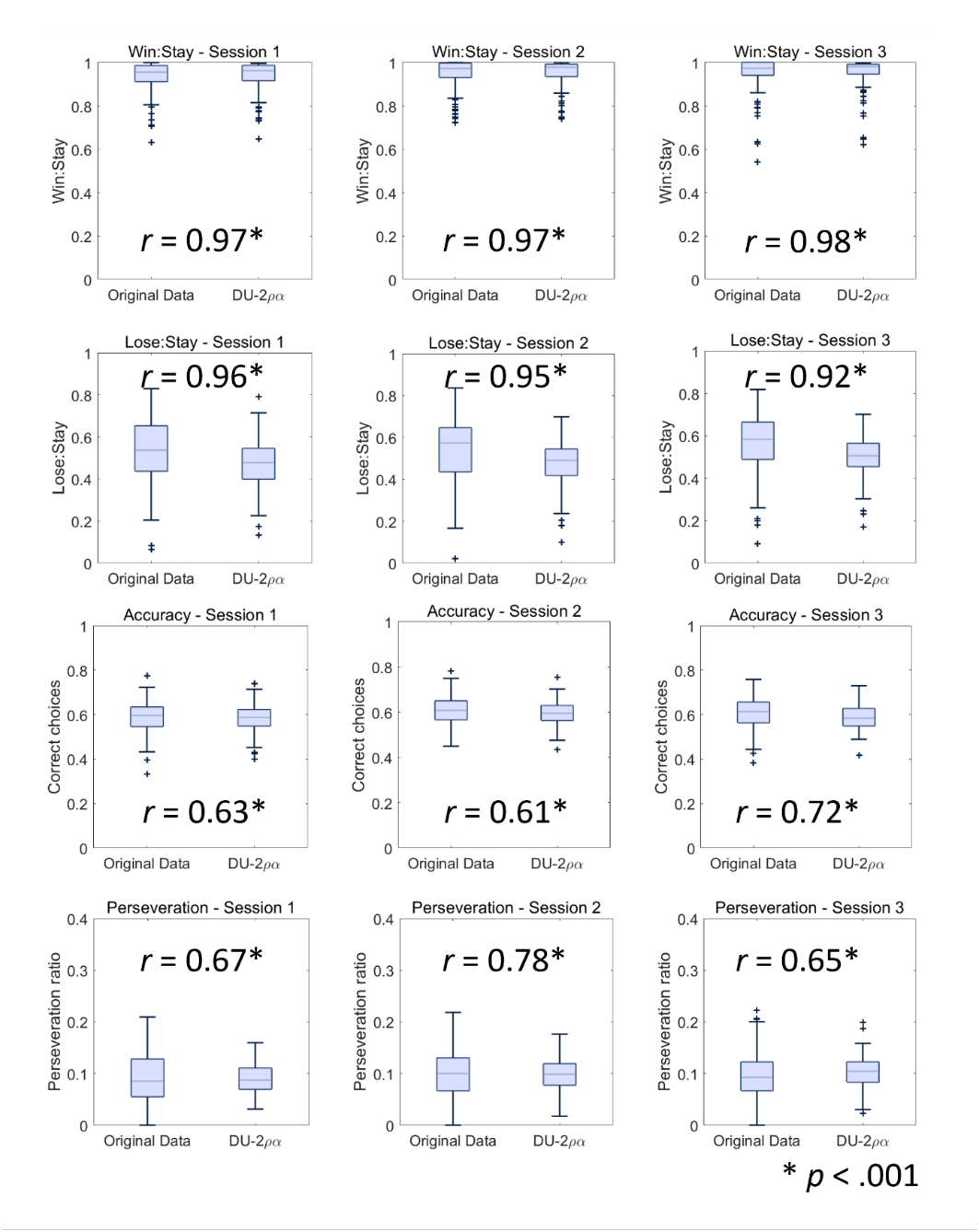


Figure S5: Moderate to high correlation between original and simulated data (100 simulations per 143 parameter combinations).

Table S5: Correlation of refitted parameters from simulated behavior.

| Parameter | Session 1 | Session 2 | Session 3 |
| --- | --- | --- | --- |
| 𝜌_win_ | .94 | .96 | .96 |
| 𝜌_loss_ | .94 | .96 | .96 |
| 𝛼 | .90 | .90 | .93 |

## Simulation

Given the probabilistic and adaptive design of the PRL task, it is not obvious which parameters are optimal for task performance. Therefore, we created 216 parameter combinations for *α* and *ρ_win/loss_* (6 per parameter) within the parameter space of estimation and computed 100 iterations based on data of the first acquisition wave for each subject. In a nutshell, optimal parameters are depending on the number of contingency changes that occur during the task, because it gets more difficult (see Supplement). The overall best accuracy (0.8) was achieved by a *α* of 0.27, *ρ_win_* of 4.45 and *ρ_loss_* of -1.04, based on a subject with two contingency changes. An increase of the learning rate seems to be beneficial for task performance, whereas the picture is not as clear for the reinforcement sensitivity parameters. An increase of *ρ_loss_* seems to be disadvantageous, meaning that the devaluation of punishments (close to zero), leads to a lower accuracy. For *ρ_win_* , up to a certain point (approximately 2) an increase, meaning the revaluation of rewards, leads to higher accuracy. The result of further increases depends on the number of contingency changes, i.e. for four or more contingency changes, a further increase seems to be disadvantageous.


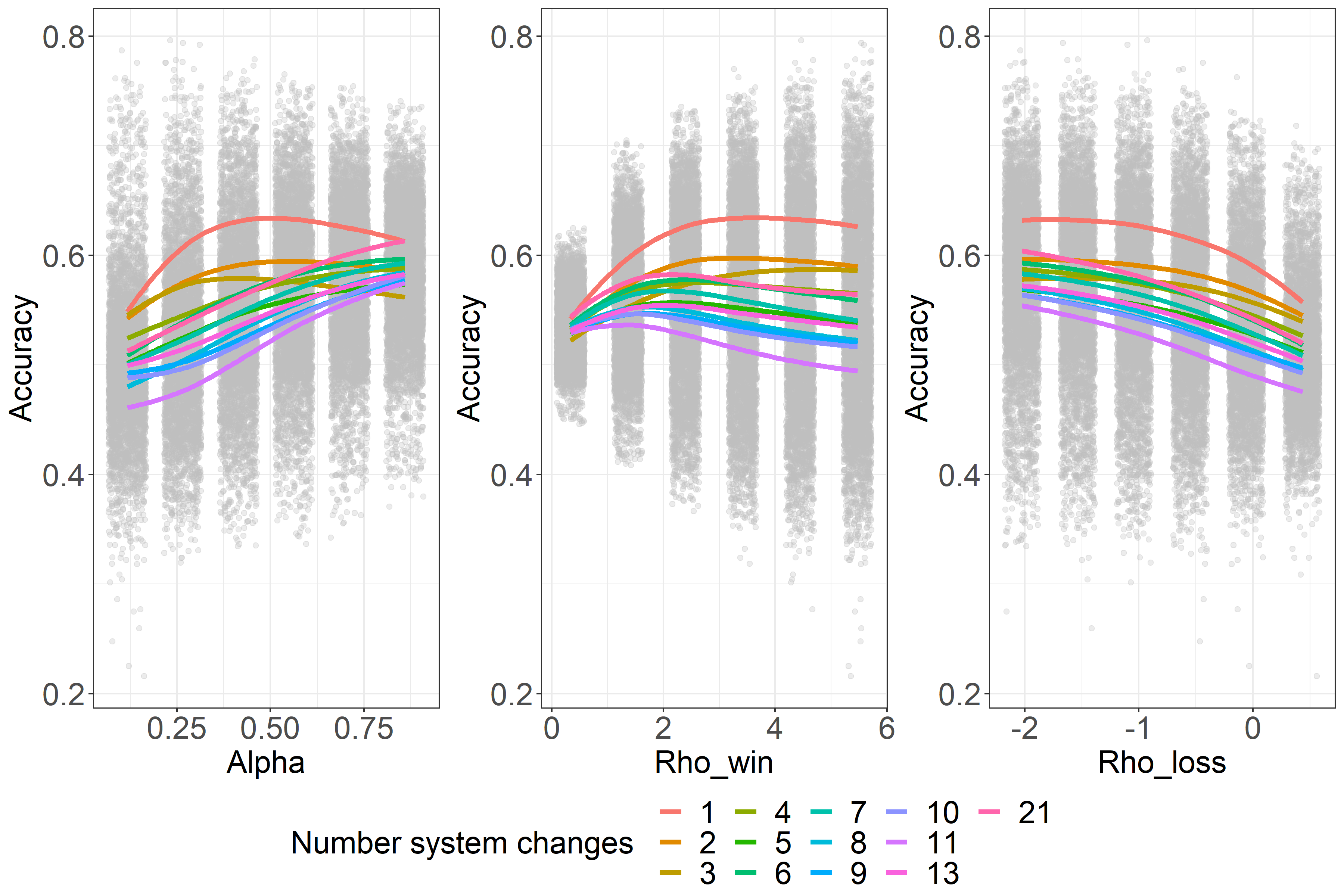


Figure S6: Simulated accuracy with computational parameters within the parameter range of the data and separated by number of system changes that occurred.

Similarly, the simulation data were employed to assess the correlation between the probability of remaining after a loss and accuracy. In other words, the objective was to ascertain whether remaining after a loss is an adaptive strategy in the context of the task at hand. Like the reinforcement learning parameters, the probability of staying after a loss is dependent on the number of contingency changes. For lower numbers of contingency changes, the likelihood of the participant staying after a loss has a negligible impact on accuracy. Conversely, as the number of contingency changes increases, the benefit of a lower probability of staying after a loss becomes more pronounced.


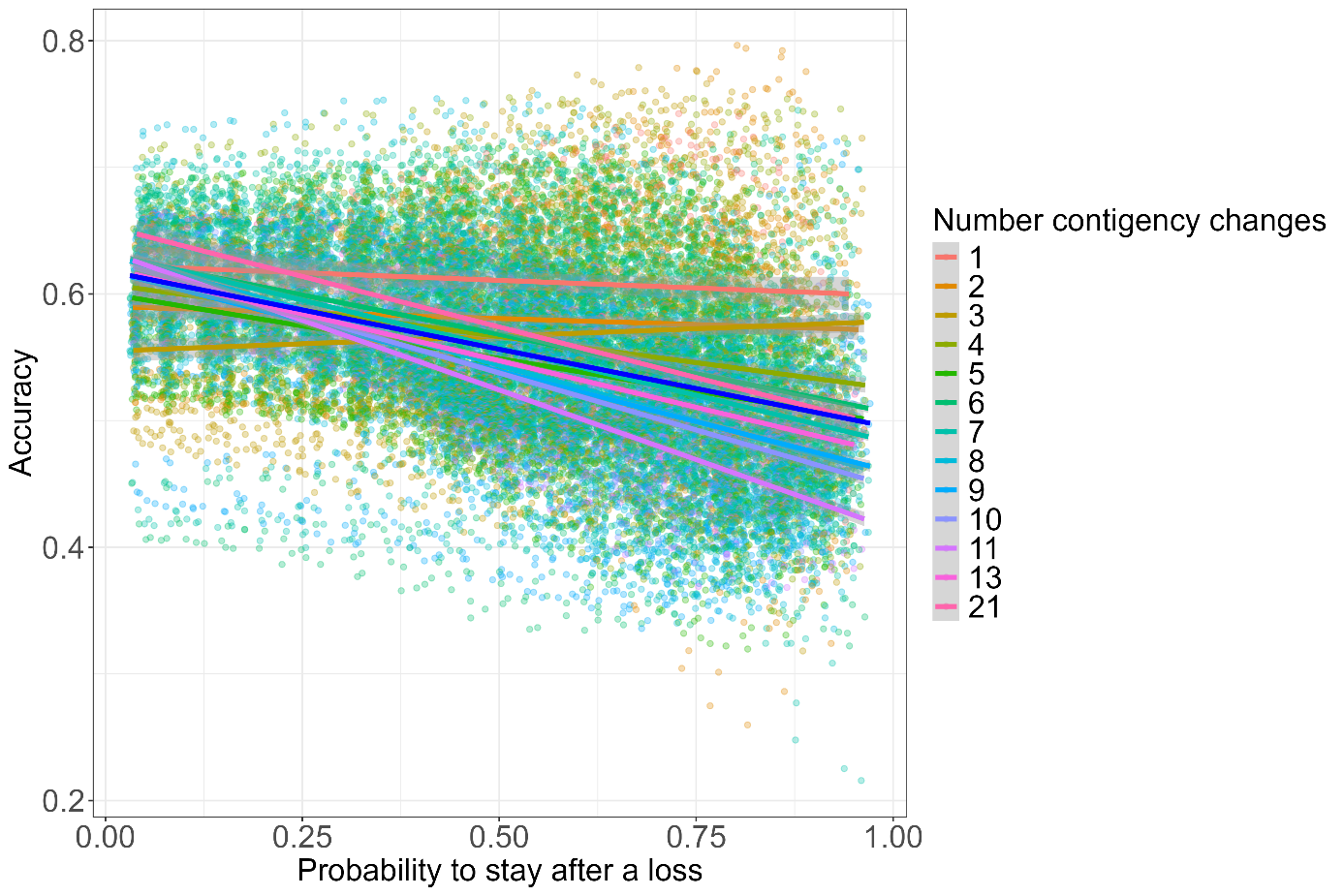


Figure S7: Simulated accuracy dependent on probability to stay after a loss, separated by number of contingency changes that occurred. The blue line represents the overall effect.

## Descriptives and correlations of computational parameters

Table S6: Pearson’s correlation and mean (M) and standard deviation (SD) for computational parameters.

| Variable |  | *Age* | *M* | *SD* | 1 | 2 | 3 | 4 | 5 | 6 | 7 | 8 |
| --- | --- | --- | --- | --- | --- | --- | --- | --- | --- | --- | --- | --- |
| Learning rate *α* | 1 | 14 | 0.61 | 0.12 |  |  |  |  |  |  |  |  |
|  |  |  |  |  |  |  |  |  |  |  |  |  |
|  | 2 | 16 | 0.62 | 0.08 | .15 |  |  |  |  |  |  |  |
|  |  |  |  |  | [-.01, .31] |  |  |  |  |  |  |  |
|  | 3 | 18 | 0.64 | 0.10 | .59** | .25** |  |  |  |  |  |  |
|  |  |  |  |  | [.47, .68] | [.09, .40] |  |  |  |  |  |  |
| Reinforcement sensitivity *ρ_win_* | 4 | 14 | 2.36 | 1.01 | -.58** | .08 | -.53** |  |  |  |  |  |
|  |  |  |  |  | [-.68, -.46] | [-.08, .24] | [-.64, -.40] |  |  |  |  |  |
|  | 5 | 16 | 2.61 | 1.06 | -.11 | -.29** | -.21* | .36** |  |  |  |  |
|  |  |  |  |  | [-.27, .06] | [-.44, -.14] | [-.36, -.05] | [.21, .50] |  |  |  |  |
|  | 6 | 18 | 2.62 | 1.00 | -.30** | .03 | -.59** | .54** | .59** |  |  |  |
|  |  |  |  |  | [-.44, -.14] | [-.14, .19] | [-.69, -.48] | [.41, .64] | [.48, .69] |  |  |  |
| Reinforcement sensitivity *ρ_loss_* | 7 | 14 | -0.77 | 0.40 | .59** | -.00 | .42** | -.55** | -.33** | -.61** |  |  |
|  |  |  |  |  | [.47, .68] | [-.17, .16] | [.28, .55] | [-.66, -.43] | [-.47, -.18] | [-.70, -.49] |  |  |
|  | 8 | 16 | -0.77 | 0.42 | .20* | .26** | .03 | .01 | -.53** | -.29** | .56** |  |
|  |  |  |  |  | [.04, .35] | [.10, .40] | [-.13, .19] | [-.16, .17] | [-.64, -.41] | [-.44, -.13] | [.43, .66] |  |
|  | 9 | 18 | -0.66 | 0.39 | .48** | -.01 | .63** | -.46** | -.28** | -.70** | .74** | .61** |
|  |  |  |  |  | [.34, .59] | [-.18, .15] | [.51, .72] | [-.58, -.32] | [-.43, -.12] | [-.78, -.61] | [.65, .80] | [.49, .70] |

## Correlation of computational parameters with behavior

Table S7: Pearson’s correlation r between computational parameters and behavioral indices

| Variable | Alpha 14 | Alpha 16 | Alpha 18 | Rho win 14 | Rho win 16 | Rho win 18 | Rho loss 14 | Rho loss 16 | Rho loss 18 |
| --- | --- | --- | --- | --- | --- | --- | --- | --- | --- |
| Accuracy 14 | 0.14 | 0.05 | 0.01 | -0.01 | -0.02 | 0.12 | .21* | 0 | 0.06 |
|  | [-.02, .30] | [-.11, .21] | [-.15, .17] | [-.18, .15] | [-.18, .14] | [-.04, .28] | [.04, .36] | [-.16, .17] | [-.11, .22] |
| Accuracy 16 | 0.03 | .22** | 0.12 | -0.05 | 0.07 | -0.01 | 0.04 | 0.16 | 0.06 |
|  | [-.13, .19] | [.06, .37] | [-.05, .28] | [-.21, .11] | [-.09, .23] | [-.18, .15] | [-.12, .20] | [-.00, .32] | [-.10, .22] |
| Accuracy 18 | 0.02 | -0.04 | 0.11 | -0.09 | -0.16 | 0.01 | 0.04 | 0.13 | 0.11 |
|  | [-.14, .19] | [-.20, .13] | [-.06, .27] | [-.25, .08] | [-.31, .01] | [-.15, .17] | [-.12, .20] | [-.04, .29] | [-.06, .27] |
| Probability to stay after win 14 | -0.1 | .17* | -.26** | .73** | .20* | .32** | .31** | -0.15 | .23** |
|  | [-.26, .07] | [.00, .32] | [-.40, -.10] | [.64, .80] | [.04, .35] | [.17, .46] | [.15, .45] | [-.31, .01] | [.07, .38] |
| Probability to stay after win 16 | 0.02 | 0.12 | 0.05 | .17* | .74** | .38** | .20* | .42** | 0.15 |
|  | [-.15, .18] | [-.04, .28] | [-.12, .21] | [.00, .32] | [.65, .81] | [.23, .51] | [.04, .35] | [.27, .55] | [-.01, .31] |
| Probability to stay after win 18 | -0.07 | 0.14 | -0.16 | .21* | .32** | .71** | .40** | .19* | .42** |
|  | [-.23, .10] | [-.02, .30] | [-.31, .01] | [.05, .36] | [.16, .46] | [.62, .78] | [.25, .53] | [.03, .35] | [.27, .54] |
| Probability to stay after loss 14 | -.51** | 0.01 | -.36** | .59** | 0.08 | 0.09 | -0.13 | -.36** | 0 |
|  | [-.62, -.37] | [-.16, .17] | [-.50, -.21] | [.47, .68] | [-.08, .24] | [-.07, .25] | [-.28, .04] | [-.49, -.20] | [-.16, .17] |
| Probability to stay after loss 16 | 0.06 | -.51** | -.19* | .18* | .43** | 0.15 | -.24** | -.32** | -.35** |
|  | [-.10, .22] | [-.62, -.38] | [-.34, -.03] | [.02, .34] | [.29, .56] | [-.01, .31] | [-.39, -.07] | [-.46, -.17] | [-.49, -.20] |
| Probability to stay after loss 18 | -0.05 | -0.06 | -.42** | .22** | .31** | .45** | 0.01 | -.39** | -0.09 |
|  | [-.22, .11] | [-.22, .11] | [-.54, -.27] | [.06, .37] | [.15, .45] | [.31, .57] | [-.15, .18] | [-.52, -.24] | [-.25, .07] |

*Note.* Values in square brackets indicate the 95% confidence interval for each correlation. * *p* < .05. ** *p* < .01. Black: Cross-sectional correlations. Grey: Longitudinal correlations.

## 2.6 Correlation of cumulative alcohol consumption and PReL behavioral and computational variables

Table S8: Pearson’s correlation r between cumulative alcohol consumption and difference scores (age 18 – age 14) of computational parameters and behavioral indices

| Variable | M | SD | 1 | 2 | 3 | 4 | 5 | 6 |
| --- | --- | --- | --- | --- | --- | --- | --- | --- |
| 1. Cumulative alcohol consumption | 5580.2 | 6582.06 |  |  |  |  |  |  |
|  |  |  |  |  |  |  |  |  |
| 2. Difference accuracy | 0.02 | 0.06 | -0.1 |  |  |  |  |  |
|  |  |  | [-.26, .06] |  |  |  |  |  |
| 3. Difference stay probability after win | 0.02 | 0.08 | -0.1 | 0.16 |  |  |  |  |
|  |  |  | [-.26, .06] | [-.01, .31] |  |  |  |  |
| 4. Difference stay probability after loss | 0.03 | 0.12 | -0.09 | -.25** | .40** |  |  |  |
|  |  |  | [-.25, .07] | [-.40, -.09] | [.26, .53] |  |  |  |
| 5. Difference alpha | 0.04 | 0.1 | 0.03 | 0.16 | 0.01 | -.45** |  |  |
|  |  |  | [-.14, .19] | [-.00, .32] | [-.16, .17] | [-.57, -.31] |  |  |
| 6. Difference rho loss | 0.11 | 0.29 | -0.06 | -.29** | -0.12 | .30** | .45** |  |
|  |  |  | [-.23, .10] | [-.43, -.13] | [-.28, .04] | [.15, .45] | [.31, .57] |  |
| 7. Difference rho win | 0.25 | 0.97 | -0.05 | -0.03 | .72** | .71** | -.42** | -.26** |
|  |  |  | [-.22, .11] | [-.19, .14] | [.63, .79] | [.61, .78] | [-.54, -.27] | [-.41, -.10] |

Note. Values in square brackets indicate the 95% confidence interval for each correlation. * p < .05. ** p < .01.

Black: Association with cumulative alcohol consumption.

# Latent growth curve models (LGCM)

## LGCM - Model fit

Table S9: Model fit for each latent growth curve model including fMRI data

| **PReL variable** | **Χ^2^ (df)** | ***p*-value** | **CFI** | **RMSEA** | **90% CI** | ***p*-value** | **SRMR** |
| --- | --- | --- | --- | --- | --- | --- | --- |
| *Behavioral and computational parameters* | | | | | | | |
| Accuracy | 2.138 (4) | 0.710 | 1.000 | 0.000 | [0.000,0.094] | 0.823 | 0.018 |
| Probability to stay after win | 1.895 (4) | 0.755 | 1.000 | 0.000 | [0.000,0.087] | 0.853 | 0.020 |
| Probability to stay after loss | 2.604 (4) | 0.626 | 1.000 | 0.000 | [0.000,0.104] | 0.761 | 0.020 |
| Learning rate *α* | 4.248 (4) | 0.374 | 0.998 | 0.021 | [0.000,0.130] | 0.546 | 0.030 |
| Reinforcement sensitivity for wins *ρ_win_* | 1.852 (4) | 0.763 | 1.000 | 0.000 | [0.000,0.086] | 0.859 | 0.019 |
| Reinforcement sensitivity for losses *ρ_loss_* | 1.134 (4) | 0.889 | 1.000 | 0.000 | [0.000,0.058] | 0.938 | 0.014 |
| *Neural correlates* | | | | | | | |
| vmPFC (choice probability) | 16.130 (4) | 0.003 | 0.87 | 0.146 | [0.076,0.223] | 0.015 | 0.068 |
| PCC (choice probability) | 10.060 (4) | 0.039 | 0.931 | 0.103 | [0.021,0.184] | 0.112 | 0.057 |
| Striatum right (SU) | 2.606 (4) | 0.626 | 1 | 0 | [0.000,0.104] | 0.761 | 0.021 |
| Striatum left (SU) | 6.966 (4) | 0.138 | 0.957 | 0.072 | [0.000,0.159] | 0.275 | 0.034 |
| Insula right (SU) | 0.469 (4) | 0.976 | 1 | 0 | [0.000, 0.000] | 0.988 | 0.011 |
| Insula left (SU) | 8.579 (4) | 0.073 | 0.940 | 0.089 | [0.000,0.173] | 0.174 | 0.047 |
| Medial frontal gyrus (SU) | 5.749 (4) | 0.219 | 0.984 | 0.055 | [0.000,0.147] | 0.38 | 0.033 |
| Striatum right (DU) | 2.363 (4) | 0.669 | 1 | 0 | [0.000,0.099] | 0.793 | 0.020 |
| Striatum left (DU) | 2.305 (4) | 0.680 | 1 | 0 | [0.000,0.097] | 0.801 | 0.025 |
| Insula right (DU) | 1.879 (4) | 0.758 | 1 | 0 | [0.000,0.087] | 0.855 | 0.020 |
| Insula left (DU) | 5.469 (4) | 0.242 | 0.98 | 0.051 | [0.000,0.144] | 0.408 | 0.033 |
| *Note*: Neural correlates were extracted from an 8mm sphere around the peak of the region for the main effect (in brackets). vmPFC = ventromedial prefrontal cortex, PCC = posterior cingulate cortex, SU = single-update prediction error, DU = double-update prediction error | | | | | | | |

Table S 10: Model fits for behavioral and computational parameters for models with and without (w/o) covariates

| **PReL variable** | **Covariates** | **Χ^2^ (df)** | ***p*-value** | **CFI** | **RMSEA** | **90% CI** | ***p*-value** | **SRMR** |
| --- | --- | --- | --- | --- | --- | --- | --- | --- |
| Accuracy | w/o | 2.138 (4) | 0.710 | 1.000 | 0.000 | [0.000,0.094] | 0.823 | 0.018 |
|  | gender | 10.113 (11) | 0.520 | 1.000 | 0.000 | [0.000,0.082] | 0.772 | 0.048 |
|  | age | 467.221 (22) | <.001 | 0.135 | 0.376 | [0.347,0.406] | <.001 | 0.207 |
|  | gender & age | 477.817 (29) | <.001 | 0.159 | 0.329 | [0.303,0.355] | <.001 | 0.192 |
| Probability to stay after win | w/o | 1.895 (4) | 0.755 | 1.000 | 0.000 | [0.000,0.087] | 0.853 | 0.020 |
|  | gender | 13.691 (11) | 0.251 | 0.976 | 0.041 | [0.000,0.102] | 0.529 | 0.059 |
|  | age | 470.608 (22) | <.001 | 0.176 | 0.378 | [0.348,0.408] | <.001 | 0.206 |
|  | gender & age | 482.621 (29) | <.001 | 0.196 | 0.331 | [0.305,0.358] | <.001 | 0.192 |
| Probability to stay after loss | w/o | 2.604 (4) | 0.626 | 1.000 | 0.000 | [0.000,0.104] | 0.761 | 0.020 |
|  | gender | 7.160 (11) | 0.786 | 1.000 | 0.000 | [0.000,0.059] | 0.924 | 0.036 |
|  | age | 469.141 (22) | <.001 | 0.352 | 0.377 | [0.377,0.348] | <.001 | 0.208 |
|  | gender & age | 475.477 (29) | <.001 | 0.366 | 0.328 | [0.303,0.355] | <.001 | 0.192 |
| Learning rate *α* | w/o | 4.248 (4) | 0.374 | 0.998 | 0.021 | [0.000,0.130] | 0.546 | 0.030 |
|  | gender | 5.551 (11) | 0.902 | 1.000 | 0.000 | [0.000,0.037] | 0.972 | 0.031 |
|  | age | 464.317 (22) | <.001 | 0.241 | 0.375 | [0.346,0.405] | <.001 | 0.207 |
|  | gender & age | 469.956 (29) | <.001 | 0.261 | 0.326 | [0.300,0.352] | <.001 | 0.191 |
| Reinforcement sensitivity for wins *ρ_win_* | w/o | 1.852 (4) | 0.763 | 1.000 | 0.000 | [0.000,0.086] | 0.859 | 0.019 |
|  | gender | 5.550 (11) | 0.902 | 1.000 | 0.000 | [0.000.0.038] | 0.971 | 0.038 |
|  | age | 464.169 (22) | <.001 | 0.293 | 0.375 | [0.346,0.405] | <.001 | 0.205 |
|  | gender & age | 472.388 (29) | <.001 | 0.309 | 0.327 | [0.301,0.353] | <.001 | 0.189 |
| Reinforcement sensitivity for losses *ρ_loss_* | w/o | 1.134 (4) | 0.889 | 1.000 | 0.000 | [0.000,0.058] | 0.938 | 0.014 |
|  | gender | 4.741 (11) | 0.943 | 1.000 | 0.000 | [0.000.0.016] | 0.985 | 0.038 |
|  | age | 468.377 (22) | <.001 | 0.363 | 0.352 | [0.325,0.380] | <.001 | 0.204 |
|  | gender & age | 474.212 (29) | <.001 | 0.374 | 0.328 | [0.302.0.354] | <.001 | 0.289 |

## LGCMs with time-varying covariate – Results tables

Table S11: Latent growth curve model estimates for estimated accuracy as time-varying covariate of drinking measured by AUDIT

| **Model** |  | **Estimate** | **SE** | **Z** | **p-value** | **95 % CI lower** | **95% CI upper** | **Standardized Estimate** |
| --- | --- | --- | --- | --- | --- | --- | --- | --- |
| Accuracy | *Covariances* | | | | | | | |
|  | ICPT & Slope AUDIT | -0.032 | 0.044 | -0.725 | 0.469 | -0.118 | 0.054 | -0.238 |
|  | Accuracy 1 & 2 | 0.048 | 0.071 | 0.679 | 0.497 | -0.091 | 0.188 | 0.048 |
|  | Accuracy 2 & 3 | -0.092 | 0.069 | -1.339 | 0.181 | -0.227 | 0.043 | -0.092 |
|  | Accuracy 1 & 3 | -0.059 | 0.094 | -0.636 | 0.525 | -0.243 | 0.124 | -0.059 |
|  | Accuracy 1 & Slope AUDIT | 0.044 | 0.031 | 1.412 | 0.158 | -0.017 | 0.105 | 0.141 |
|  | Accuracy 2 & Slope AUDIT | -0.002 | 0.023 | -0.071 | 0.943 | -0.046 | 0.043 | -0.005 |
|  | Accuracy 3 & Slope AUDIT | 0.016 | 0.042 | 0.377 | 0.706 | -0.066 | 0.098 | 0.051 |
|  | *Intercepts* | | | | | | | |
|  | ICPT AUDIT | -0.635 | 0.044 | -14.327 | < 0.001 | -0.722 | -0.548 | -1.484 |
|  | Slope AUDIT | 0.386 | 0.030 | 12.697 | < 0.001 | 0.326 | 0.445 | 1.233 |
|  | *Regressions* | | | | | | | |
|  | Accuracy 1 → AUDIT 1 | 0.027 | 0.040 | 0.683 | 0.495 | -0.050 | 0.104 | 0.027 |
|  | Accuracy 2 → AUDIT 2 | -0.068 | 0.083 | -0.821 | 0.412 | -0.231 | 0.095 | -0.068 |
|  | Accuracy 3 → AUDIT 3 | -0.050 | 0.096 | -0.519 | 0.604 | -0.237 | 0.138 | -0.050 |
|  | *Residual variances* | | | | | | | |
|  | ICPT AUDIT | 0.183 | 0.121 | 1.514 | 0.130 | -0.054 | 0.420 | 1.000 |
|  | Slope AUDIT | 0.098 | 0.023 | 4.201 | < 0.001 | 0.052 | 0.144 | 1.000 |
|  | AUDIT 1 | 0.078 | 0.119 | 0.652 | 0.514 | -0.156 | 0.312 | 0.078 |
|  | AUDIT 2 | 0.526 | 0.080 | 6.606 | 3.95E-11 | 0.370 | 0.682 | 0.526 |
|  | AUDIT 3 | 0.188 | 0.105 | 1.790 | 0.073 | -0.018 | 0.394 | 0.188 |
|  | Accuracy 1 | 1.159 | 0.137 | 8.456 | < 0.001 | 0.890 | 1.428 | 1.159 |
|  | Accuracy 2 | 0.623 | 0.074 | 8.456 | < 0.001 | 0.478 | 0.767 | 0.623 |
|  | Accuracy 3 | 1.076 | 0.127 | 8.456 | < 0.001 | 0.827 | 1.326 | 1.076 |
| *Note*: ICPT: Intercept (model-defined baseline); AUDIT: Alcohol Use Disorder Identification Test (5) | | | | | | | | |

Table S12: Latent growth curve model estimates for estimated probability to stay after a win as time-varying covariate of drinking measured by AUDIT

| **Model** |  | **Estimate** | **SE** | **Z** | **p-value** | **95 % CI lower** | **95% CI upper** | **Standardized Estimate** |
| --- | --- | --- | --- | --- | --- | --- | --- | --- |
| Probability to stay after a win | *Covariances* | | | | | | | |
|  | ICPT & Slope AUDIT | -0.035 | 0.044 | -0.796 | 0.426 | -0.120 | 0.051 | -0.244 |
|  | Stay win 1 & 2 | 0.113 | 0.077 | 1.470 | 0.142 | -0.038 | 0.263 | 0.113 |
|  | Stay win 2 & 3 | 0.380 | 0.089 | 4.280 | 1.87E-05 | 0.206 | 0.554 | 0.380 |
|  | Stay win 1 & 3 | 0.210 | 0.088 | 2.396 | 0.017 | 0.038 | 0.382 | 0.210 |
|  | Stay win 1 & Slope AUDIT | 0.018 | 0.029 | 0.611 | 0.541 | -0.040 | 0.075 | 0.055 |
|  | Stay win 2 & Slope AUDIT | -0.022 | 0.031 | -0.709 | 0.478 | -0.082 | 0.038 | -0.067 |
|  | Stay win 3 & Slope AUDIT | -0.099 | 0.049 | -2.025 | 0.043 | -0.194 | -0.003 | -0.305 |
|  | *Intercepts* | | | | | | | |
|  | ICPT AUDIT | -0.636 | 0.043 | -14.622 | < 0.001 | -0.721 | -0.551 | -1.449 |
|  | Slope AUDIT | 0.379 | 0.031 | 12.266 | < 0.001 | 0.318 | 0.439 | 1.168 |
|  | *Regressions* | | | | | | | |
|  | Stay win 1 → AUDIT 1 | 0.038 | 0.044 | 0.862 | 0.388 | -0.048 | 0.123 | 0.038 |
|  | Stay win 2 → AUDIT 2 | -0.037 | 0.078 | -0.476 | 0.634 | -0.191 | 0.116 | -0.037 |
|  | Stay win 3 → AUDIT 3 | 0.123 | 0.109 | 1.129 | 0.259 | -0.091 | 0.337 | 0.123 |
|  | *Residual variances* | | | | | | | |
|  | ICPT AUDIT | 0.192 | 0.120 | 1.607 | 0.108 | -0.042 | 0.427 | 1.000 |
|  | Slope AUDIT | 0.105 | 0.024 | 4.320 | < 0.001 | 0.057 | 0.153 | 1.000 |
|  | AUDIT 1 | 0.069 | 0.118 | 0.583 | 0.560 | -0.162 | 0.300 | 0.069 |
|  | AUDIT 2 | 0.522 | 0.079 | 6.638 | 3.17E-11 | 0.368 | 0.677 | 0.522 |
|  | AUDIT 3 | 0.180 | 0.102 | 1.765 | 0.078 | -0.020 | 0.379 | 0.180 |
|  | Stay win 1 | 0.941 | 0.111 | 8.456 | < 0.001 | 0.723 | 1.159 | 0.941 |
|  | Stay win 2 | 0.877 | 0.104 | 8.456 | < 0.001 | 0.674 | 1.080 | 0.877 |
|  | Stay win 3 | 1.120 | 0.132 | 8.456 | < 0.001 | 0.860 | 1.380 | 1.120 |

*Note*: ICPT: Intercept (model-defined baseline); AUDIT: Alcohol Use Disorder Identification Test (Saunders, Aasland, Babor, de la Fuente, & Grant, 1993)

Table S13: Latent growth curve model estimates for estimated probability to stay after a loss as time-varying covariate of drinking measured by AUDIT

| **Model** |  | **Estimate** | **SE** | **Z** | **p-value** | **95 % CI lower** | **95% CI upper** | **Standardized Estimate** |
| --- | --- | --- | --- | --- | --- | --- | --- | --- |
| Probability to stay after a loss | *Covariances* | | | | | | | |
|  | ICPT & Slope AUDIT | -0.023 | 0.043 | -0.547 | 0.584 | -0.107 | 0.060 | -0.181 |
|  | Stay loss 1 & 2 | 0.489 | 0.101 | 4.830 | 1.37E-06 | 0.291 | 0.688 | 0.489 |
|  | Stay loss 2 & 3 | 0.687 | 0.094 | 7.284 | 3.25E-13 | 0.502 | 0.872 | 0.687 |
|  | Stay loss 1 & 3 | 0.398 | 0.083 | 4.807 | 1.53E-06 | 0.236 | 0.560 | 0.398 |
|  | Stay loss 1 & Slope AUDIT | -0.013 | 0.038 | -0.331 | 0.741 | -0.088 | 0.063 | -0.040 |
|  | Stay loss 2 & Slope AUDIT | -0.055 | 0.050 | -1.080 | 0.280 | -0.153 | 0.044 | -0.170 |
|  | Stay loss 3 & Slope AUDIT | -0.064 | 0.048 | -1.337 | 0.181 | -0.158 | 0.030 | -0.200 |
|  | *Intercepts* | | | | | | | |
|  | ICPT AUDIT | -0.640 | 0.043 | -14.815 | < 0.001 | -0.725 | -0.556 | -1.590 |
|  | Slope AUDIT | 0.369 | 0.032 | 11.684 | < 0.001 | 0.307 | 0.431 | 1.154 |
|  | *Regressions* | | | | | | | |
|  | Stay loss 1 → AUDIT 1 | 0.002 | 0.040 | 0.047 | 0.963 | -0.077 | 0.081 | 0.002 |
|  | Stay loss 2 → AUDIT 2 | -0.026 | 0.096 | -0.271 | 0.787 | -0.215 | 0.163 | -0.026 |
|  | Stay loss 3 → AUDIT 3 | 0.319 | 0.174 | 1.838 | 0.066 | -0.021 | 0.659 | 0.319 |
|  | *Residual variances* | | | | | | | |
|  | ICPT AUDIT | 0.162 | 0.117 | 1.388 | 0.165 | -0.067 | 0.391 | 1.000 |
|  | Slope AUDIT | 0.102 | 0.024 | 4.205 | < 0.001 | 0.055 | 0.150 | 1.000 |
|  | AUDIT 1 | 0.100 | 0.116 | 0.863 | 0.388 | -0.127 | 0.327 | 0.100 |
|  | AUDIT 2 | 0.499 | 0.076 | 6.535 | 6.36E-11 | 0.349 | 0.649 | 0.499 |
|  | AUDIT 3 | 0.161 | 0.102 | 1.584 | 0.113 | -0.038 | 0.360 | 0.161 |
|  | Stay loss 1 | 1.122 | 0.133 | 8.456 | < 0.001 | 0.862 | 1.382 | 1.122 |
|  | Stay loss 2 | 1.095 | 0.129 | 8.456 | < 0.001 | 0.841 | 1.348 | 1.095 |
|  | Stay loss 3 | 0.731 | 0.086 | 8.456 | < 0.001 | 0.562 | 0.901 | 0.731 |

*Note*: ICPT: Intercept (model-defined baseline); AUDIT: Alcohol Use Disorder Identification Test (Saunders, Aasland, Babor, de la Fuente, & Grant, 1993)

Table S14: Latent growth curve model estimates for learning rate as time-varying covariate of drinking measured by AUDIT

| **Model** |  | **Estimate** | **SE** | **Z** | **p-value** | **95 % CI lower** | **95% CI upper** | **Standardized Estimate** |
| --- | --- | --- | --- | --- | --- | --- | --- | --- |
| Learning rate | *Covariances* | | | | | | | |
|  | ICPT & Slope AUDIT | -0.059 | 0.085 | -0.695 | 0.487 | -0.224 | 0.107 | -0.326 |
|  | Learning rate 1 & 2 | 0.149 | 0.082 | 1.830 | 0.067 | -0.011 | 0.309 | 0.149 |
|  | Learning rate 2 & 3 | 0.190 | 0.066 | 2.903 | 0.004 | 0.062 | 0.319 | 0.190 |
|  | Learning rate 1 & 3 | 0.645 | 0.107 | 6.050 | 1.45E-09 | 0.436 | 0.854 | 0.645 |
|  | Learning rate 1 & Slope AUDIT | 0.015 | 0.034 | 0.433 | 0.665 | -0.052 | 0.081 | 0.049 |
|  | Learning rate 2 & Slope AUDIT | -0.007 | 0.029 | -0.247 | 0.805 | -0.064 | 0.050 | -0.024 |
|  | Learning rate 3 & Slope AUDIT | 0.023 | 0.042 | 0.537 | 0.591 | -0.060 | 0.106 | 0.075 |
|  | *Intercepts* | | | | | | | |
|  | ICPT AUDIT | -0.646 | 0.043 | -14.974 | < 0.001 | -0.731 | -0.562 | -1.539 |
|  | Slope AUDIT | 0.388 | 0.030 | 12.956 | < 0.001 | 0.330 | 0.447 | 1.241 |
|  | *Regressions* | | | | | | | |
|  | Learning rate 1 → AUDIT 1 | -0.016 | 0.035 | -0.455 | 0.649 | -0.085 | 0.053 | -0.016 |
|  | Learning rate 2 → AUDIT 2 | 0.017 | 0.083 | 0.211 | 0.833 | -0.144 | 0.179 | 0.017 |
|  | Learning rate 3 → AUDIT 3 | -0.034 | 0.074 | -0.461 | 0.645 | -0.179 | 0.111 | -0.034 |
|  | *Residual variances* | | | | | | | |
|  | ICPT AUDIT | 0.176 | 0.121 | 1.458 | 0.145 | -0.061 | 0.413 | 1.000 |
|  | Slope AUDIT | 0.098 | 0.023 | 4.169 | < 0.001 | 0.052 | 0.144 | 1.000 |
|  | AUDIT 1 | 0.085 | 0.120 | 0.715 | 0.475 | -0.149 | 0.320 | 0.085 |
|  | AUDIT 2 | 0.537 | 0.081 | 6.638 | 3.18E-11 | 0.378 | 0.695 | 0.537 |
|  | AUDIT 3 | 0.167 | 0.105 | 1.595 | 0.111 | -0.038 | 0.372 | 0.167 |
|  | Learning rate 1 | 1.393 | 0.165 | 8.456 | < 0.001 | 1.070 | 1.716 | 1.393 |
|  | Learning rate 2 | 0.667 | 0.079 | 8.456 | < 0.001 | 0.512 | 0.821 | 0.667 |
|  | Learning rate 3 | 0.869 | 0.103 | 8.456 | < 0.001 | 0.667 | 1.070 | 0.869 |

*Note*: ICPT: Intercept (model-defined baseline); AUDIT: Alcohol Use Disorder Identification Test (Saunders, Aasland, Babor, de la Fuente, & Grant, 1993)

Table S15: Latent growth curve model estimates for reinforcement sensitivity (rho) for wins as time-varying covariate of drinking measured by AUDIT

| **Model** |  | **Estimate** | **SE** | **Z** | **p-value** | **95 % CI lower** | **95% CI upper** | **Standardized Estimate** |
| --- | --- | --- | --- | --- | --- | --- | --- | --- |
| Rho win | *Covariances* | | | | | | | |
|  | ICPT & Slope AUDIT | -0.028 | 0.043 | -0.652 | 0.514 | -0.112 | 0.056 | -0.213 |
|  | Rho win 1 & 2 | 0.368 | 0.090 | 4.099 | 4.14E-05 | 0.192 | 0.544 | 0.368 |
|  | Rho win 2 & 3 | 0.591 | 0.097 | 6.112 | 9.83E-10 | 0.402 | 0.781 | 0.591 |
|  | Rho win 1 & 3 | 0.508 | 0.090 | 5.651 | 1.60E-08 | 0.331 | 0.684 | 0.508 |
|  | Rho win 1 & Slope AUDIT | -0.014 | 0.035 | -0.415 | 0.678 | -0.083 | 0.054 | -0.046 |
|  | Rho win 2 & Slope AUDIT | -0.019 | 0.039 | -0.496 | 0.620 | -0.095 | 0.057 | -0.061 |
|  | Rho win 3 & Slope AUDIT | -0.040 | 0.046 | -0.853 | 0.394 | -0.130 | 0.051 | -0.126 |
|  | *Intercepts* | | | | | | | |
|  | ICPT AUDIT | -0.633 | 0.043 | -14.686 | < 0.001 | -0.717 | -0.548 | -1.517 |
|  | Slope AUDIT | 0.380 | 0.030 | 12.619 | < 0.001 | 0.321 | 0.439 | 1.208 |
|  | *Regressions* | | | | | | | |
|  | Rho win 1 → AUDIT 1 | 0.062 | 0.043 | 1.429 | 0.153 | -0.023 | 0.146 | 0.062 |
|  | Rho win 2 → AUDIT 2 | -0.080 | 0.079 | -1.015 | 0.310 | -0.234 | 0.074 | -0.080 |
|  | Rho win 3 → AUDIT 3 | 0.102 | 0.128 | 0.797 | 0.425 | -0.149 | 0.354 | 0.102 |
|  | *Residual variances* | | | | | | | |
|  | ICPT AUDIT | 0.174 | 0.118 | 1.478 | 0.139 | -0.057 | 0.404 | 1.000 |
|  | Slope AUDIT | 0.099 | 0.023 | 4.242 | < 0.001 | 0.053 | 0.145 | 1.000 |
|  | AUDIT 1 | 0.085 | 0.116 | 0.730 | 0.466 | -0.143 | 0.313 | 0.085 |
|  | AUDIT 2 | 0.517 | 0.078 | 6.591 | 4.37E-11 | 0.363 | 0.671 | 0.517 |
|  | AUDIT 3 | 0.173 | 0.103 | 1.674 | 0.094 | -0.030 | 0.375 | 0.173 |
|  | Rho win 1 | 0.961 | 0.114 | 8.456 | < 0.001 | 0.738 | 1.183 | 0.961 |
|  | Rho win 2 | 1.060 | 0.125 | 8.456 | < 0.001 | 0.815 | 1.306 | 1.060 |
|  | Rho win 3 | 0.933 | 0.110 | 8.456 | < 0.001 | 0.716 | 1.149 | 0.933 |

*Note*: ICPT: Intercept (model-defined baseline); AUDIT: Alcohol Use Disorder Identification Test (Saunders, Aasland, Babor, de la Fuente, & Grant, 1993)

Table S16: Latent growth curve model estimates for reinforcement sensitivity (rho) for losses as time-varying covariate of drinking measured by AUDIT

| **Model** |  | **Estimate** | **SE** | **Z** | **p-value** | **95 % CI lower** | **95% CI upper** | **Standardized Estimate** |
| --- | --- | --- | --- | --- | --- | --- | --- | --- |
| Rho loss | *Covariances* | | | | | | | |
|  | ICPT & Slope AUDIT | -0.028 | 0.043 | -0.656 | 0.512 | -0.113 | 0.057 | -0.214 |
|  | Rho loss 1 & 2 | 0.560 | 0.096 | 5.805 | 6.44E-09 | 0.371 | 0.749 | 0.560 |
|  | Rho loss 2 & 3 | 0.599 | 0.096 | 6.213 | 5.21E-10 | 0.410 | 0.788 | 0.599 |
|  | Rho loss 1 & 3 | 0.696 | 0.098 | 7.085 | 1.40E-12 | 0.504 | 0.889 | 0.696 |
|  | Rho loss 1 & Slope AUDIT | 0.023 | 0.040 | 0.581 | 0.561 | -0.055 | 0.101 | 0.074 |
|  | Rho loss 2 & Slope AUDIT | 0.002 | 0.039 | 0.052 | 0.958 | -0.074 | 0.078 | 0.006 |
|  | Rho loss 3 & Slope AUDIT | 0.004 | 0.046 | 0.086 | 0.931 | -0.086 | 0.094 | 0.013 |
|  | *Intercepts* | | | | | | | |
|  | ICPT AUDIT | -0.649 | 0.043 | -15.194 | < 0.001 | -0.732 | -0.565 | -1.515 |
|  | Slope AUDIT | 0.387 | 0.030 | 12.830 | < 0.001 | 0.328 | 0.446 | 1.244 |
|  | *Regressions* | | | | | | | |
|  | Rho loss 1 → AUDIT 1 | -0.058 | 0.043 | -1.353 | 0.176 | -0.142 | 0.026 | -0.058 |
|  | Rho loss 2 → AUDIT 2 | -0.059 | 0.080 | -0.736 | 0.462 | -0.215 | 0.098 | -0.059 |
|  | Rho loss 3 → AUDIT 3 | 0.001 | 0.127 | 0.005 | 0.996 | -0.248 | 0.250 | 0.001 |
|  | *Residual variances* | | | | | | | |
|  | ICPT AUDIT | 0.183 | 0.119 | 1.536 | 0.125 | -0.051 | 0.418 | 1.000 |
|  | Slope AUDIT | 0.097 | 0.023 | 4.168 | < 0.001 | 0.051 | 0.142 | 1.000 |
|  | AUDIT 1 | 0.076 | 0.118 | 0.642 | 0.521 | -0.155 | 0.307 | 0.076 |
|  | AUDIT 2 | 0.533 | 0.080 | 6.628 | 3.40E-11 | 0.376 | 0.691 | 0.533 |
|  | AUDIT 3 | 0.173 | 0.104 | 1.659 | 0.097 | -0.031 | 0.378 | 0.173 |
|  | Rho loss 1 | 0.969 | 0.115 | 8.456 | < 0.001 | 0.745 | 1.194 | 0.969 |
|  | Rho loss 2 | 1.049 | 0.124 | 8.456 | < 0.001 | 0.806 | 1.292 | 1.049 |
|  | Rho loss 3 | 0.925 | 0.109 | 8.456 | < 0.001 | 0.711 | 1.140 | 0.925 |
| *Note:* ICPT: Intercept (model-defined baseline); AUDIT: Alcohol Use Disorder Identification Test (5) | | | | | | | | |

# fMRI analyses

## fMRI acquisition and preprocessing

The imaging data was acquired at the Neuroimaging Centre at the Technische Universität Dresden with a 3.0 T scanner (Magnetom Tim Trio, Siemens, Erlangen, Germany). The anatomical images for co-registration were acquired using a T1-weighted magnetisation-prepared, rapid acquisition gradient echo (MP-RAGE) sequence (field of view (FoV): 256x224 mm^2^, 176 slices, voxel size: 1x1x1 mm^3^, repetition time (TR): 1900 ms, echo time: 2.26 ms, flip angle (FA): 9°). Series of T2*-weighted echo-planar images (EPI) with 42 transverse slices tilted approximately 30° towards the coronal beyond the anterior to posterior commissure line, with a 3mm in-plane resolution was used field of view (FoV): 192x192 mm^2^, voxel size: 3x3x3 mm^3^, repetition time (TR): 2410 ms, echo time: 25 ms, flip angle (FA): 80°). The first three volumes were discarded to allow the magnetization to reach equilibrium.

During preprocessing, images were slice-time corrected and realigned based on voxel-displacement maps computed from field maps. The images were spatially normalized to Montreal Neurological Institute (MNI) space using normalization parameters generated during the segmentation of the individual T1-weighted structural image (6). All images were spatially smoothed with an isotropic Gaussian kernel (6mm full-width at half-maximum). We individually checked field-map correction, normalization, and head motion. We had to exclude five subjects due to normalization issues and excessive head motion (> 3mm or 3° per volume). Before computing first-level statistics, we used a high-pass filter (cut-off: 128s).

## Main effects

For our fMRI analyses, we defined three contrasts of interest: choice probability as parametric modulator of stimulus onset, and single- and double-update prediction error signal as parametric modulator of feedback onset. We decided to focus on theory-driven regions of interest for the following analyses and associations with alcohol use (see Table S18 for main effects and ROIs). Therefore, signal was extracted for an 8mm-sphere around the ROI peaks.

Table S17: Main effects over all sessions (p_FWE-corrected_ < .05, cluster size > 50) separated by contrast. Regions of interest (ROI) highlighted in blue. Light blue: Cluster in the left hippocampus included the left striatal ROI (MNI coordinates [-12,5,-11], t = 7.12, p_FWE-corrected_ < .001). The right striatal ROI was slightly smaller than 50 voxels (MNI coordinates [12,5,-11], t = 6.18, p_FWE-corrected_ < .001).

| **Region** | **peak MNI coordinates** | | | **cluster size** | ***t*** | **peak *p*-value (FWE-corrected)** |
| --- | --- | --- | --- | --- | --- | --- |
|  | **x** | **y** | **z** |  |  |  |
| *Choice probability signal* | | | | | | |
| Posterior cingulate cortex | -6 | -52 | 19 | 1926 | 14.53 | 3.32E-11 |
| Angular gyrus | -42 | -73 | 34 | 1793 | 14.48 | 3.32E-11 |
| Ventromedial prefrontal cortex | 0 | 50 | -11 | 1056 | 13.26 | 3.32E-11 |
| Hippocampus | 24 | -16 | -17 | 246 | 9.54 | 3.32E-11 |
| Superior temporal gyrus | 63 | -7 | 1 | 1261 | 9.19 | 3.32E-11 |
| Superior frontal gyrus | -21 | 29 | 46 | 122 | 7.82 | 1.11E-09 |
| *Single-update signal - positive* | | | | | | |
| Striatum (right) | 12 | 5 | -8 | 353 | 14.88 | 3.13E-11 |
| Striatum (left) | -15 | 2 | -11 | 370 | 13.62 | 3.13E-11 |
| Inferior- / orbitofrontal cortex | -39 | 38 | -11 | 146 | 6.98 | 2.90E-07 |
| *Single-update signal - negative* | | | | | | |
| Supplementary motor cortex | -3 | 17 | 46 | 1532 | 15.35 | 2.97E-11 |
| (Anterior) insula right | 36 | 20 | 7 | 351 | 13.84 | 2.97E-11 |
| (Anterior) insula left | -39 | 14 | 4 | 353 | 13.09 | 2.97E-11 |
| Medial frontal gyrus | -33 | 50 | 19 | 578 | 9.90 | 2.97E-11 |
| Precuneus | 12 | -67 | 37 | 167 | 8.97 | 2.97E-11 |
| Brainstem | 0 | -25 | -20 | 207 | 8.68 | 2.97E-11 |
| Cerebellum | 36 | -49 | -32 | 104 | 7.34 | 2.79E-08 |
| Supramarginal gyrus right | 51 | -43 | 31 | 191 | 6.88 | 5.62E-07 |
| *Double-update signal - positive* | | | | | | |
| Precuneus (left) | -6 | -55 | 13 | 243 | 8.51 | 5.83E-12 |
| Medial prefrontal cortex | -3 | 47 | -11 | 200 | 7.48 | 1.45E-05 |
| Hippocampus (left) | -21 | -19 | -14 | 149 | 7.33 | 2.77E-05 |
| Superior frontal gyrus (left) | -18 | 32 | 46 | 161 | 7.24 | 1.12E-08 |
| Angular gyrus (left) | -48 | -67 | 28 | 84 | 6.74 | 4.24E-06 |
| Middle temporal gyrus (left) | -60 | -52 | -11 | 86 | 6.68 | 4.66E-06 |
| Superior temporal gyrus (left) | -60 | -13 | -5 | 72 | 6.38 | 3.00E-08 |
| *Double-update signal - negative* | | | | | | |
| Thalamus | 6 | -25 | 1 | 66 | 6.99 | 2.89E-07 |
| Anterior Insula (left) | -30 | 20 | -8 | 92 | 6.87 | 5.83E-07 |
| Anterior Insula (right) | 39 | 20 | -8 | 146 | 6.83 | 7.53E-07 |

*Note*: Brain regions were assigned to coordinates according to the brain atlas of *Neuromorphometrics, Inc.*and double-checked by the neurosynth data base (7).


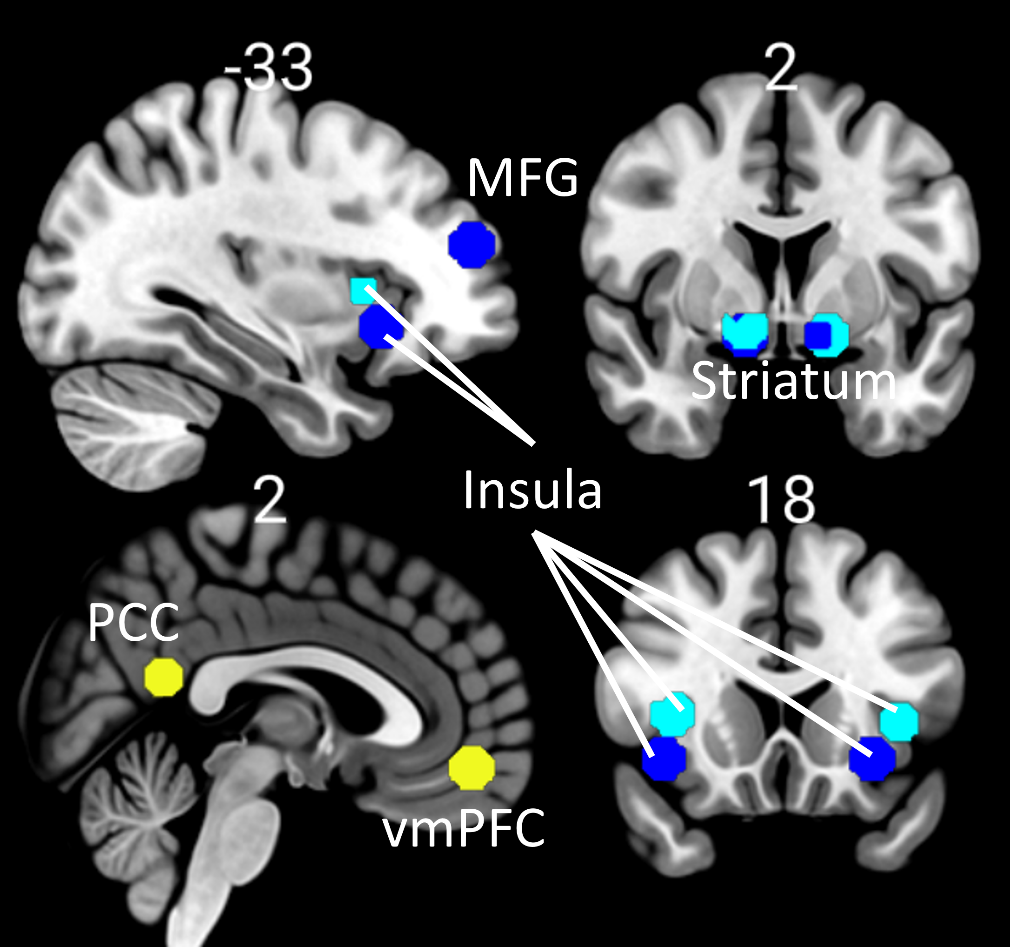


Figure S8: Regions of interest defined by theory-driven selection of main-effect peaks. Yellow: Choice probability signal in ventromedial prefrontal cortex (vmPFC) and posterior cingulate cortex (PCC). Blue: Single-update prediction error in striatum, insula and medial frontal gyrus (MFG). Turquoise: Double-update prediction error in striatum and insula.

## Correlation of ROI signal with behavior

Table S18: Correlation of signal in regions of interest with estimated behavior

|  | | Left Striatum (DU) | Right Striatum (DU) | Right Insula (DU) | Left Insula (DU) | vmPFC (CP) | PCC (CP) | Right Striatum (SU) | Left Striatum (SU) | Right Insula (SU) | Left Insula (SU) | MFG (SU) |
| --- | --- | --- | --- | --- | --- | --- | --- | --- | --- | --- | --- | --- |
| *1st wave* | | | | | | | | | | | | |
| Accuracy | Pearson's *r* | 0.080 | 0.067 | 0.104 | 0.064 | -0.037 | -0.022 | -0.040 | 0.024 | 0.051 | 0.046 | -0.072 |
|  | *p*-value | 0.345 | 0.426 | 0.219 | 0.447 | 0.658 | 0.793 | 0.636 | 0.779 | 0.544 | 0.587 | 0.392 |
| Probability to stay after win | Pearson's *r* | 0.001 | -0.069 | -.165* | -0.162 | -0.102 | -0.152 | -0.109 | -0.054 | -0.038 | -0.012 | -.214* |
|  | *p*-value | 0.994 | 0.414 | 0.049 | 0.053 | 0.225 | 0.069 | 0.196 | 0.523 | 0.654 | 0.888 | 0.010 |
| Probability to stay after loss | Pearson's *r* | 0.000 | -0.081 | -.238** | -0.133 | -0.069 | -0.138 | 0.000 | -0.011 | 0.028 | 0.040 | -0.011 |
|  | *p*-value | 0.998 | 0.339 | 0.004 | 0.114 | 0.414 | 0.101 | 0.997 | 0.893 | 0.739 | 0.635 | 0.897 |
| *2nd wave* | | | | | | | | | | | | |
| Accuracy | Pearson's *r* | -0.107 | -0.065 | -0.178* | -0.093 | -0.003 | -0.033 | -0.061 | 0.042 | -0.003 | -0.023 | 0.213* |
|  | *p*-value | 0.204 | 0.443 | 0.033 | 0.267 | 0.973 | 0.695 | 0.473 | 0.617 | 0.974 | 0.786 | 0.011 |
| Probability to stay after win | Pearson's *r* | -0.105 | -.167* | -.277** | -.255** | 0.010 | -0.041 | 0.026 | 0.119 | -0.068 | -0.092 | -0.052 |
|  | *p*-value | 0.212 | 0.046 | 0.001 | 0.002 | 0.907 | 0.626 | 0.760 | 0.156 | 0.420 | 0.273 | 0.534 |
| Probability to stay after loss | Pearson's *r* | 0.032 | 0.005 | 0.095 | 0.080 | -0.051 | 0.042 | -0.086 | -0.065 | -0.037 | -0.037 | -0.077 |
|  | *p*-value | 0.701 | 0.951 | 0.259 | 0.345 | 0.547 | 0.620 | 0.310 | 0.440 | 0.658 | 0.662 | 0.359 |
| *3rd wave* | | | | | | | | | | | | |
| Accuracy | Pearson's *r* | 0.062 | 0.038 | 0.095 | 0.028 | -0.077 | -0.094 | 0.146 | -0.008 | -0.009 | -0.107 | -0.166* |
|  | *p*-value | 0.459 | 0.656 | 0.258 | 0.737 | 0.358 | 0.264 | 0.082 | 0.927 | 0.920 | 0.203 | 0.048 |
| Probability to stay after win | Pearson's *r* | 0.037 | 0.044 | -0.058 | -0.030 | -0.045 | -0.048 | -0.054 | 0.057 | 0.059 | -0.052 | -0.142 |
|  | *p*-value | 0.659 | 0.598 | 0.493 | 0.722 | 0.597 | 0.573 | 0.524 | 0.498 | 0.485 | 0.536 | 0.091 |
| Probability to stay after loss | Pearson's *r* | 0.001 | 0.031 | 0.003 | 0.021 | 0.015 | 0.039 | -0.120 | -0.086 | -0.045 | -0.072 | -0.055 |
|  | *p*-value | 0.989 | 0.715 | 0.971 | 0.806 | 0.859 | 0.647 | 0.152 | 0.306 | 0.594 | 0.392 | 0.511 |

Note: DU = double-update prediction error, CP = choice probability, SU = single-update prediction error, vmPFC = ventromedial prefrontal cortex, PCC = posterior cingulate cortex, MFG = medial frontal gyrus. *: *p* < .05, **: *p* < .01

## Development of signal in ROIs


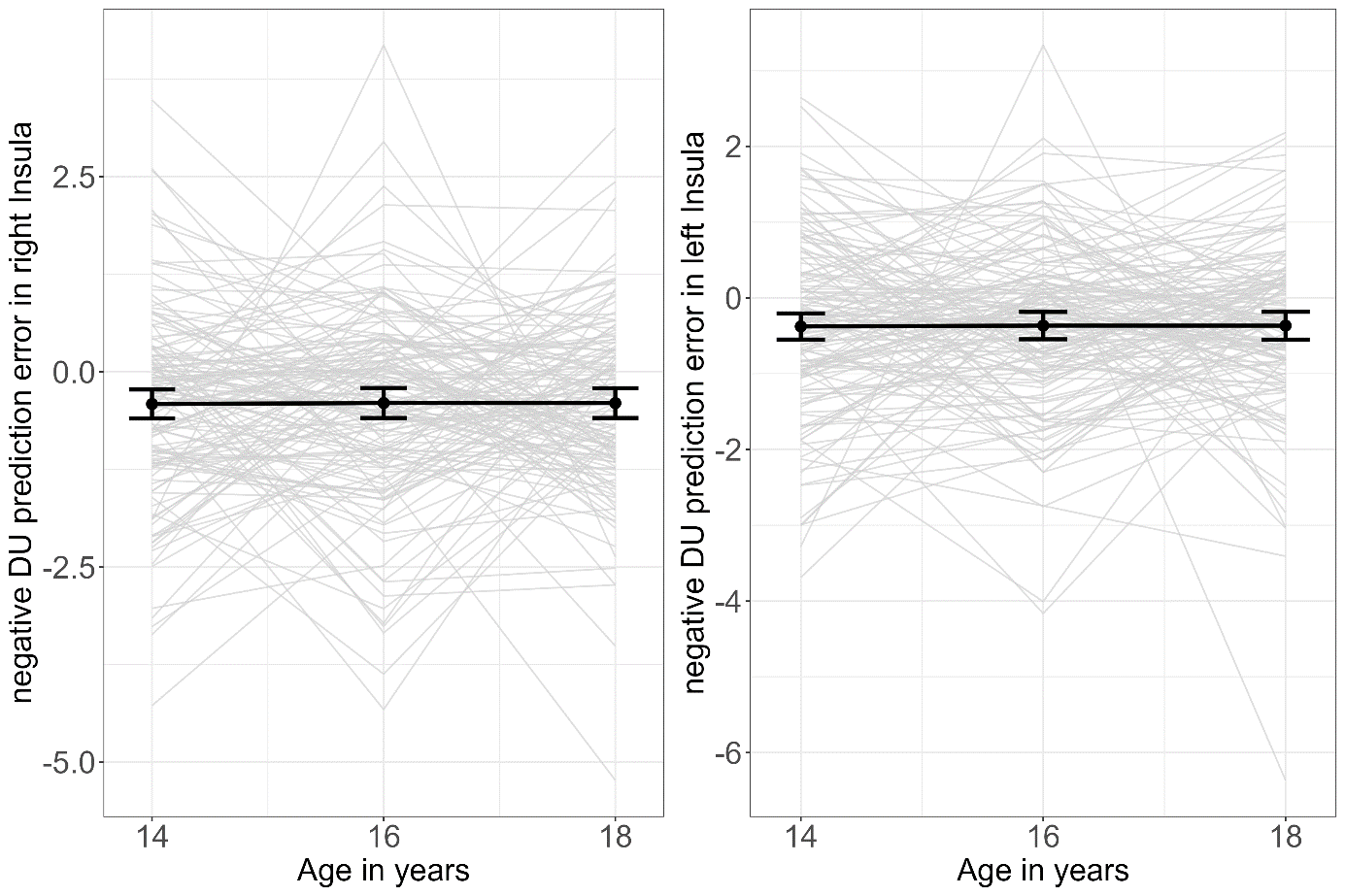

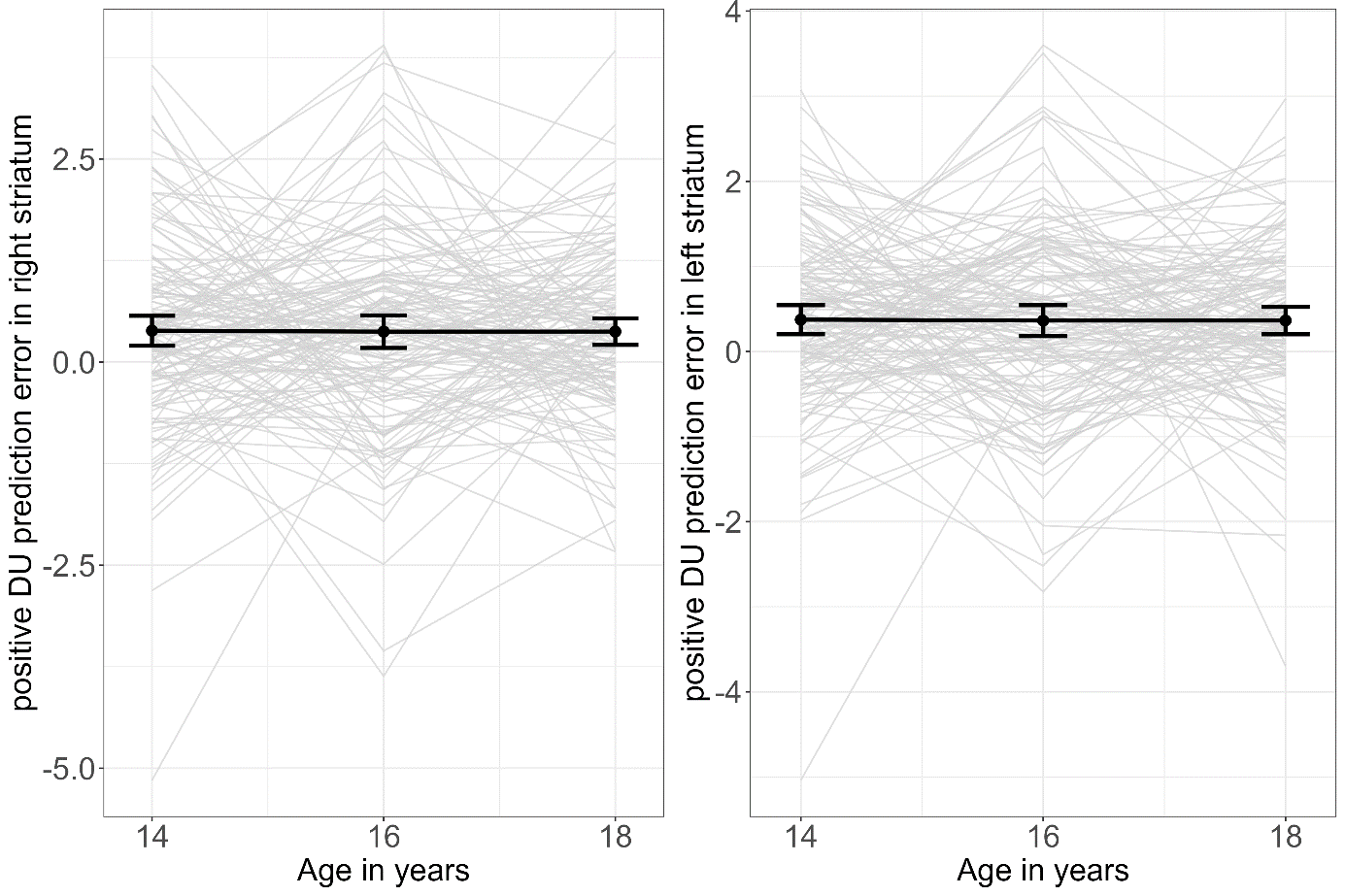


Figure S9: Development of double-update (DU) prediction error signal. Two top panels: negative association of prediction error and insula signal. Two bottom panels: positive association of prediction error and striatal signal.


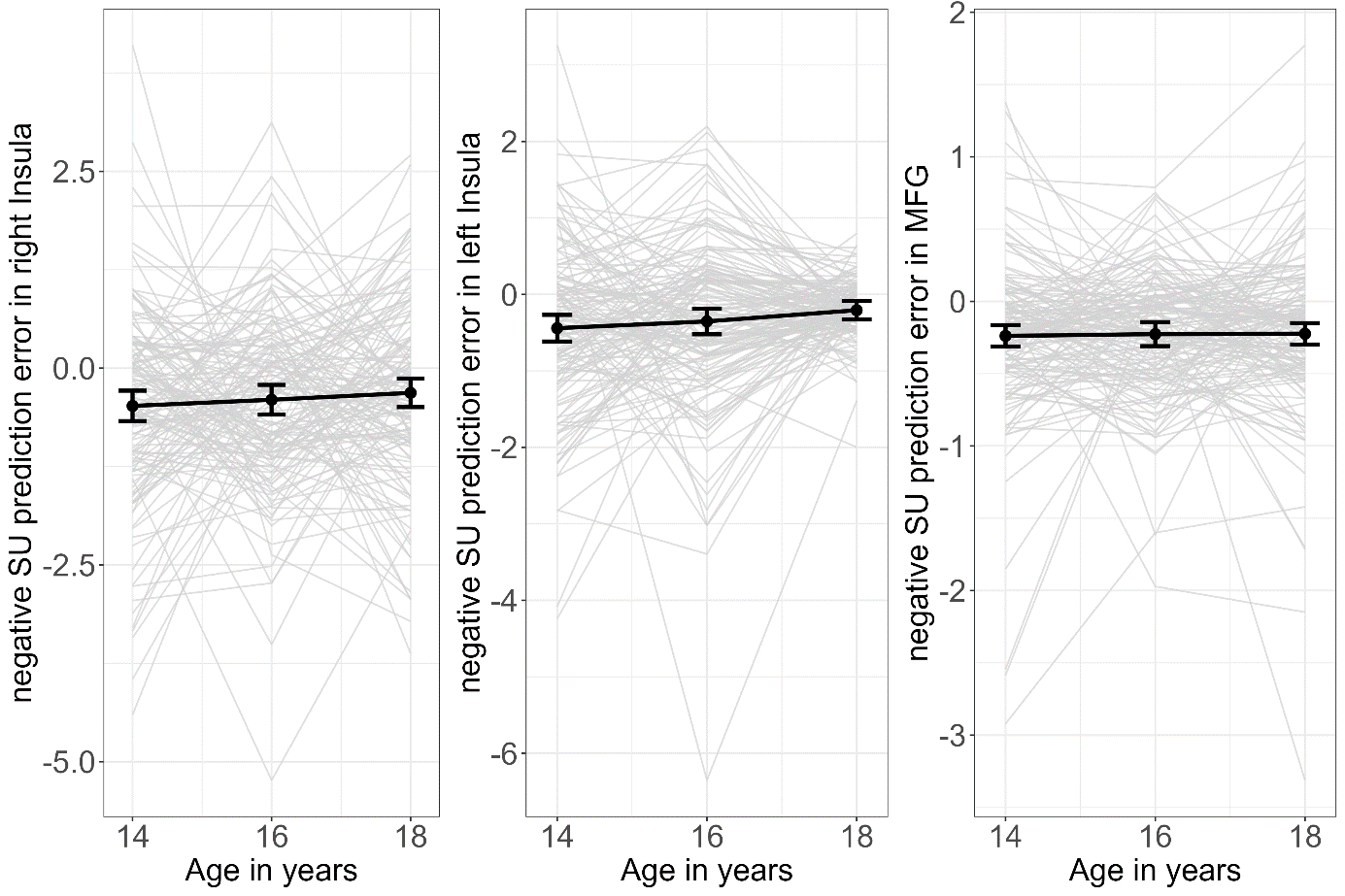

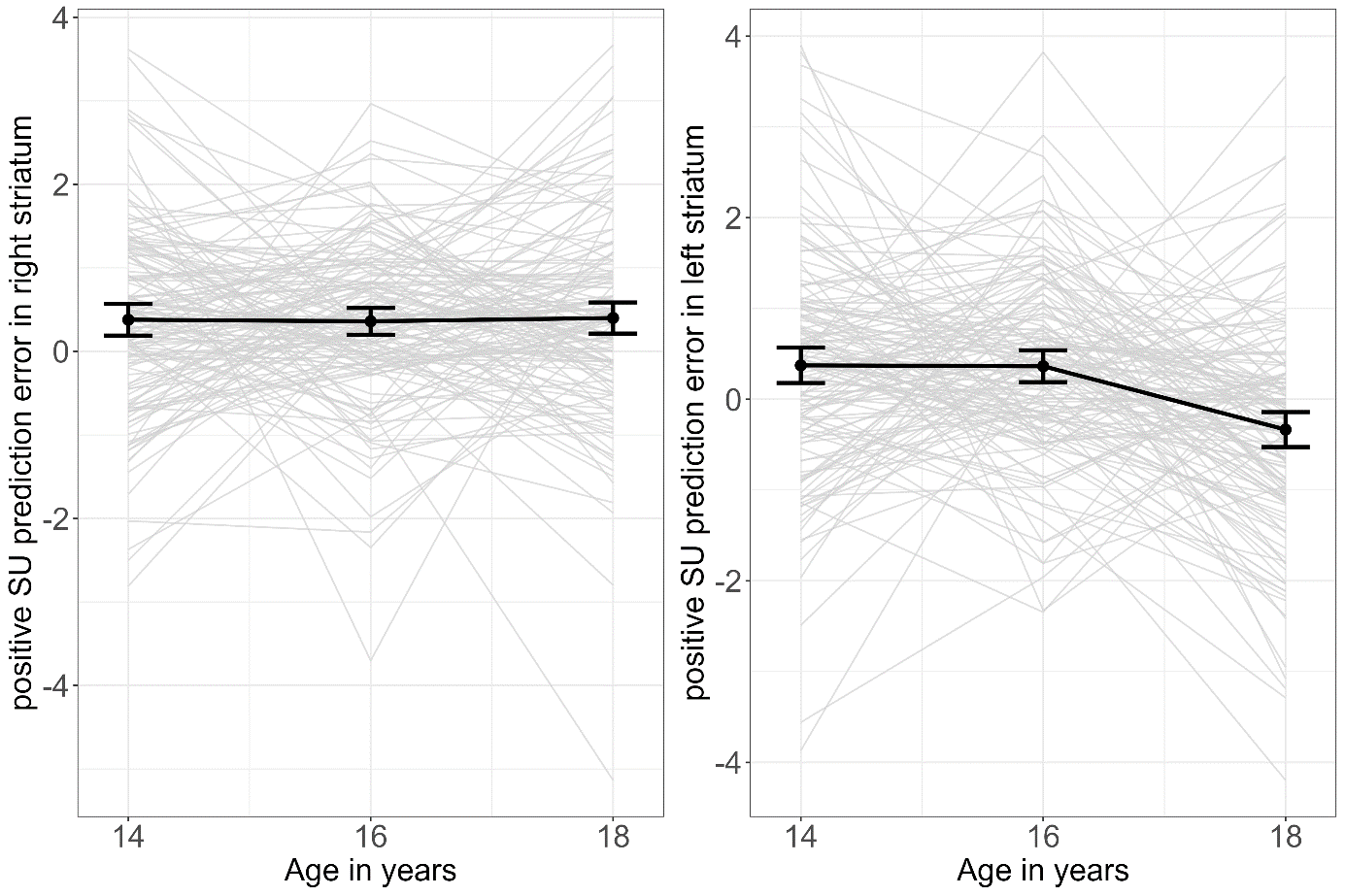


Figure S10: Development of single-update (SU) prediction error signal. Three top panels: negative association of prediction error and insula and medial frontal gyrus (MFG) signal. Two bottom panels: positive association of prediction error and striatal signal. Striatal signal (left) is significantly decreasing and insular signal (left) is significantly increasing (see Table S18).


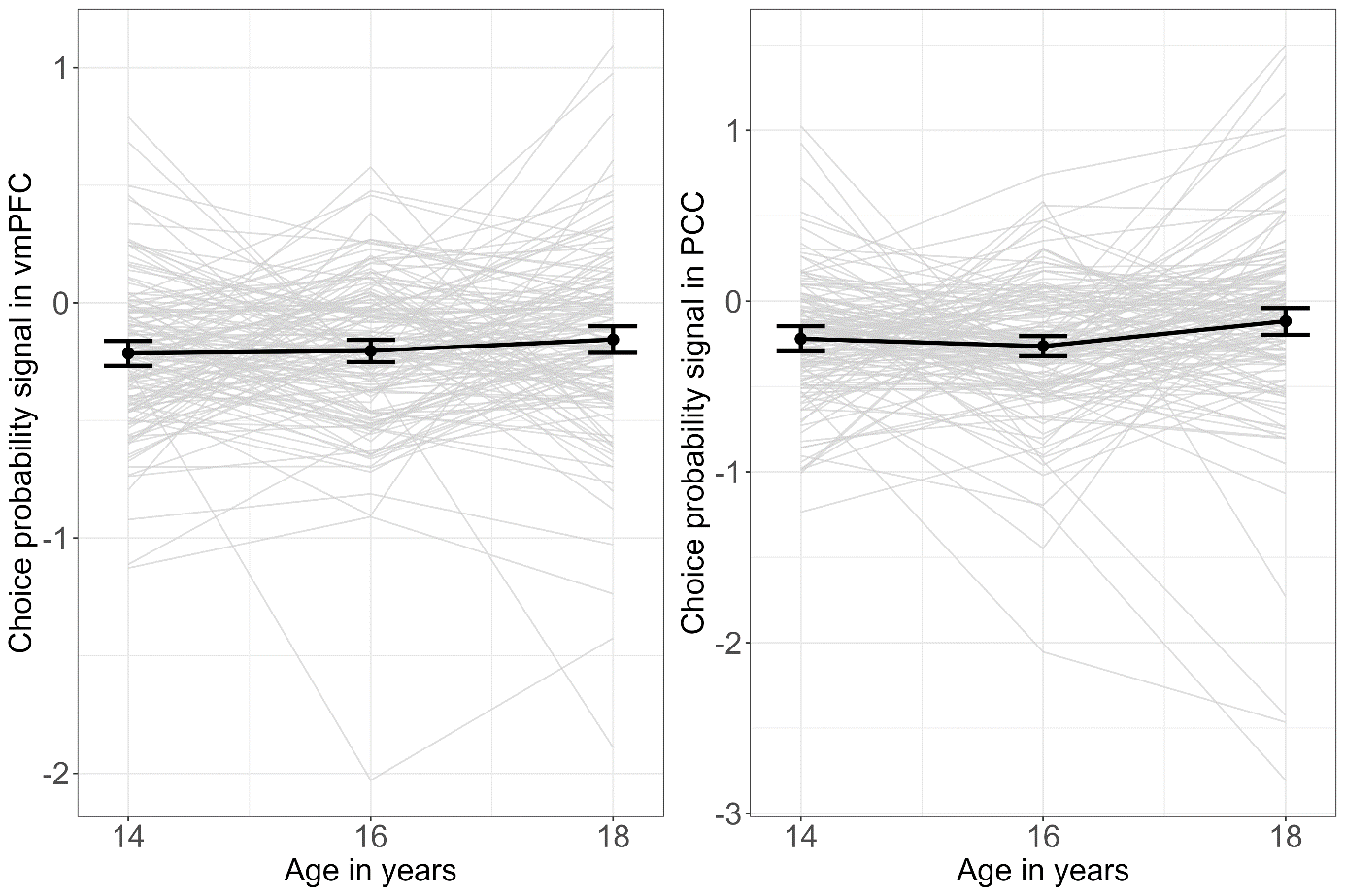


Figure S11: Development of choice-probability signal in ventromedial prefrontal cortex (vmPFC) and posterior cingulate cortex (PCC).

Table S19: Mixed effects regions for regions of interest in main effects of choice probability and prediction errors

|  |  | ***β*** | ***z-value*** | ***p*** |
| --- | --- | --- | --- | --- |
| Choice probability | vmPFC | 0.03 | 1.44 | .150 |
|  | PCC | 0.05 | 1.76 | .079 |
| SU positive | Striatum (right) | 0.01 | 0.15 | .884 |
|  | Striatum (left) | -0.35 | -4.97 | <.001** |
| SU negative | Insula (right) | 0.08 | 1.24 | .217 |
|  | Insula (left) | 0.12 | 2.05 | .041* |
|  | MFG | 0.01 | 0.27 | .790 |
| DU positive | Striatum (left) | -0.01 | -0.08 | .933 |
|  | Striatum (right) | -0.01 | -0.08 | .935 |
| DU negative | Insula (right) | 0.01 | 0.08 | .934 |
|  | Insula (left) | 0.01 | 0.08 | .936 |
| Note: vmPFC = ventromedial prefrontal cortex, PCC = posterior cingulate cortex, MFG = medial frontal gyrus, SU = single-update prediction error, DU = double-update prediction error; * *p* < .05, ** *p* < .01 | | | | |

## Second level analysis with AUDIT as time-varying covariate

Table S20: Main effect of AUDIT over all sessions (p_FWE-corrected_ < .05).

| Region | peak MNI coordinates | | | cluster size | t | peak p-value (FWE-corrected) | peak p-value (uncorrected) |
| --- | --- | --- | --- | --- | --- | --- | --- |
|  | x | y | z |  |  |  |  |
| *Single-update signal - negative* | | | | | | | |
| Medial prefrontal cortex | -12 | 44 | 10 | 65 | 5.50 | 0.002 | 3.38E-08 |
| *Note*: Brain regions were assigned to coordinates according to the brain atlas of *Neuromorphometrics, Inc.* implemented in SPM12 and double-checked by using the neurosynth data base (7) | | | | | | | |


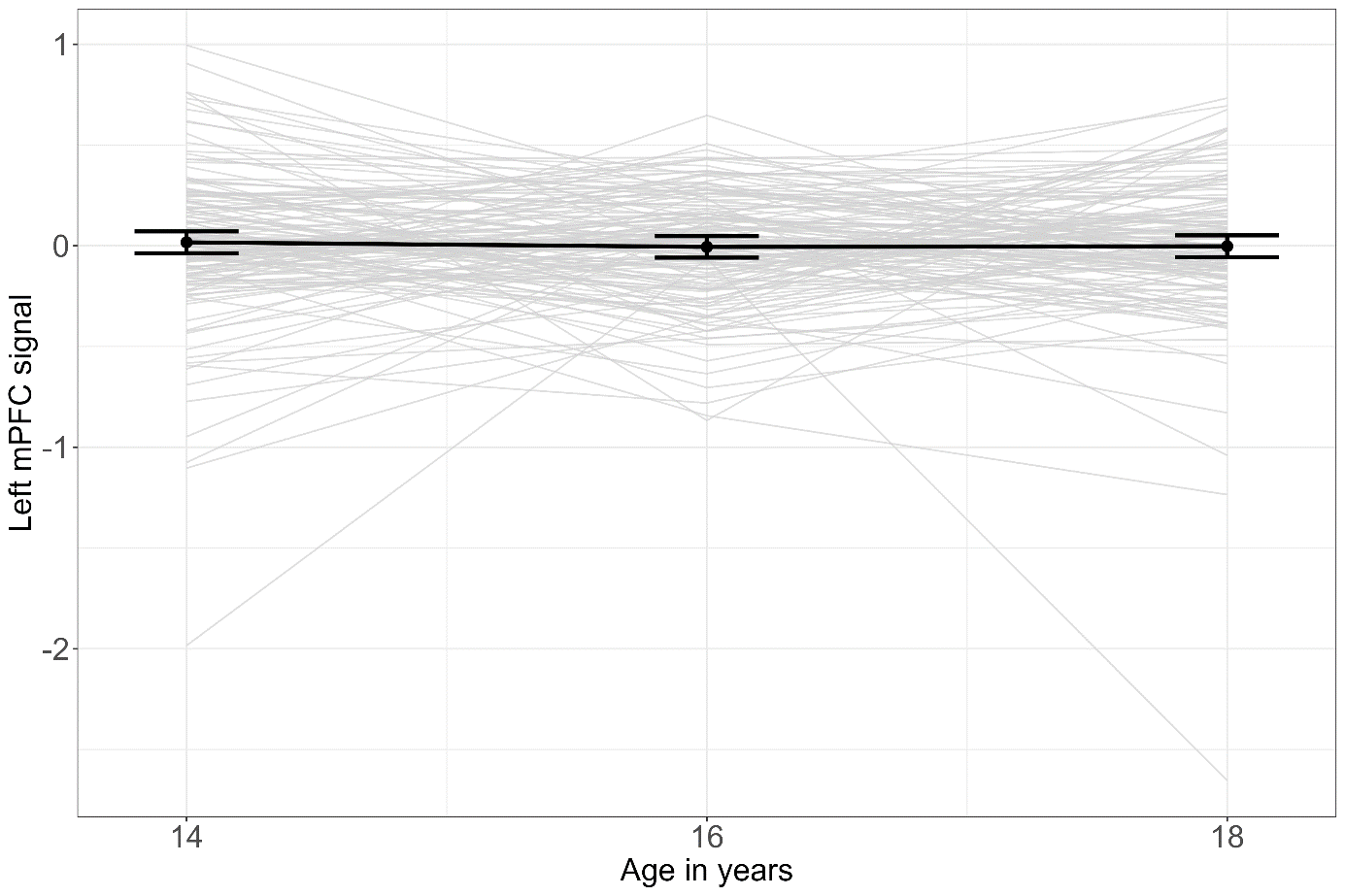


Figure S12: Development of single-update prediction error signal that was negatively associated with drinking.

## LGCMs AUDIT and neural correlates

Table S21: Latent growth curve model estimates for choice probability signal in ventromedial prefrontal cortex as time-varying covariate of drinking

| **Model** |  | **Estimate** | **SE** | **Z** | **p-value** | **95 % CI lower** | **95% CI upper** | **Standardized Estimate** |
| --- | --- | --- | --- | --- | --- | --- | --- | --- |
| Choice probability vmPFC | *Covariances* | | | | | | | |
|  | ICPT & Slope AUDIT | -0.052 | 0.047 | -1.104 | 0.269 | -0.143 | 0.040 | -0.323 |
|  | vmPFC 1 & 2 | 0.151 | 0.071 | 2.121 | 0.034 | 0.011 | 0.291 | 0.151 |
|  | vmPFC 2 & 3 | 0.278 | 0.093 | 2.998 | 0.003 | 0.096 | 0.460 | 0.278 |
|  | vmPFC 1 & 3 | 0.047 | 0.085 | 0.551 | 0.582 | -0.119 | 0.212 | 0.047 |
|  | vmPFC 1 & Slope AUDIT | -0.034 | 0.026 | -1.310 | 0.190 | -0.085 | 0.017 | -0.104 |
|  | vmPFC 2 & Slope AUDIT | -0.038 | 0.029 | -1.309 | 0.191 | -0.095 | 0.019 | -0.116 |
|  | vmPFC 3 & Slope AUDIT | -0.039 | 0.048 | -0.815 | 0.415 | -0.132 | 0.054 | -0.118 |
|  | *Intercepts* | | | | | | | |
|  | ICPT AUDIT | -0.641 | 0.043 | -14.918 | < 0.001 | -0.726 | -0.557 | -1.314 |
|  | Slope AUDIT | 0.383 | 0.030 | 12.752 | < 0.001 | 0.324 | 0.442 | 1.168 |
|  | *Regressions* | | | | | | | |
|  | vmPFC 1 → AUDIT 1 | 0.039 | 0.048 | 0.804 | 0.421 | -0.056 | 0.133 | 0.039 |
|  | vmPFC 2 → AUDIT 2 | 0.129 | 0.074 | 1.732 | 0.083 | -0.017 | 0.274 | 0.129 |
|  | vmPFC 3 → AUDIT 3 | 0.070 | 0.090 | 0.775 | 0.438 | -0.107 | 0.246 | 0.070 |
|  | *Residual variances* | | | | | | | |
|  | ICPT AUDIT | 0.238 | 0.130 | 1.837 | 0.066 | -0.016 | 0.492 | 1.000 |
|  | Slope AUDIT | 0.107 | 0.024 | 4.449 | 0.000 | 0.060 | 0.155 | 1.000 |
|  | AUDIT 1 | 0.024 | 0.126 | 0.191 | 0.849 | -0.223 | 0.272 | 0.024 |
|  | AUDIT 2 | 0.528 | 0.080 | 6.593 | < 0.001 | 0.371 | 0.685 | 0.528 |
|  | AUDIT 3 | 0.166 | 0.104 | 1.591 | 0.112 | -0.038 | 0.370 | 0.166 |
|  | vmPFC 1 | 0.790 | 0.093 | 8.456 | < 0.001 | 0.607 | 0.973 | 0.790 |
|  | vmPFC 2 | 0.893 | 0.106 | 8.456 | < 0.001 | 0.686 | 1.100 | 0.893 |
|  | vmPFC 3 | 1.294 | 0.153 | 8.456 | < 0.001 | 0.994 | 1.593 | 1.294 |

*Note:* vmPFC = ventromedial prefrontal cortex; AUDIT = Alcohol Use Disorder Identification Test (Saunders, Aasland, Babor, de la Fuente, & Grant, 1993)

Table S22: Latent growth curve model estimates for choice probability signal in posterior cingulate cortex as time-varying covariate of drinking

| **Model** |  | **Estimate** | **SE** | **Z** | **p-value** | **95 % CI lower** | **95% CI upper** | **Standardized Estimate** |
| --- | --- | --- | --- | --- | --- | --- | --- | --- |
| Choice probability PCC | *Covariances* | | | | | | | |
|  | ICPT & Slope AUDIT | -0.039 | 0.046 | -0.850 | 0.395 | -0.129 | 0.051 | -0.273 |
|  | PCC 1 & 2 | 0.016 | 0.057 | 0.289 | 0.773 | -0.095 | 0.128 | 0.016 |
|  | PCC 2 & 3 | 0.360 | 0.095 | 3.771 | < 0.001 | 0.173 | 0.546 | 0.360 |
|  | PCC 1 & 3 | -0.039 | 0.083 | -0.467 | 0.640 | -0.201 | 0.124 | -0.039 |
|  | PCC 1 & Slope AUDIT | -0.014 | 0.023 | -0.634 | 0.526 | -0.059 | 0.030 | -0.045 |
|  | PCC 2 & Slope AUDIT | -0.035 | 0.027 | -1.269 | 0.204 | -0.088 | 0.019 | -0.107 |
|  | PCC 3 & Slope AUDIT | -0.075 | 0.054 | -1.382 | 0.167 | -0.182 | 0.031 | -0.233 |
|  | *Intercepts* | | | | | | | |
|  | ICPT AUDIT | -0.642 | 0.043 | -14.939 | < 0.001 | -0.726 | -0.558 | -1.446 |
|  | Slope AUDIT | 0.382 | 0.031 | 12.491 | < 0.001 | 0.322 | 0.442 | 1.182 |
|  | *Regressions* | | | | | | | |
|  | PCC 1 → AUDIT 1 | 0.022 | 0.054 | 0.404 | 0.686 | -0.084 | 0.127 | 0.022 |
|  | PCC 2 → AUDIT 2 | 0.098 | 0.081 | 1.220 | 0.223 | -0.060 | 0.256 | 0.098 |
|  | PCC 3 → AUDIT 3 | 0.077 | 0.085 | 0.909 | 0.363 | -0.089 | 0.243 | 0.077 |
|  | *Residual variances* | | | | | | | |
|  | ICPT AUDIT | 0.197 | 0.127 | 1.549 | 0.121 | -0.052 | 0.446 | 1.000 |
|  | Slope AUDIT | 0.104 | 0.024 | 4.309 | < 0.001 | 0.057 | 0.152 | 1.000 |
|  | AUDIT 1 | 0.065 | 0.125 | 0.520 | 0.603 | -0.180 | 0.311 | 0.065 |
|  | AUDIT 2 | 0.529 | 0.080 | 6.625 | < 0.001 | 0.373 | 0.686 | 0.529 |
|  | AUDIT 3 | 0.173 | 0.104 | 1.661 | 0.097 | -0.031 | 0.376 | 0.173 |
|  | PCC 1 | 0.620 | 0.073 | 8.456 | < 0.001 | 0.476 | 0.763 | 0.620 |
|  | PCC 2 | 0.742 | 0.088 | 8.456 | < 0.001 | 0.570 | 0.913 | 0.742 |
|  | PCC 3 | 1.579 | 0.187 | 8.456 | < 0.001 | 1.213 | 1.945 | 1.579 |

*Note:* PCC = posterior cingulate cortex; AUDIT = Alcohol Use Disorder Identification Test (Saunders, Aasland, Babor, de la Fuente, & Grant, 1993)

Table S23: Latent growth curve model estimates for single-update prediction error in right striatum as time-varying covariate of drinking

| **Model** |  | **Estimate** | **SE** | **Z** | **p-value** | **95 % CI lower** | **95% CI upper** | **Standardized Estimate** |  |
| --- | --- | --- | --- | --- | --- | --- | --- | --- | --- |
| Striatum right (SU) | *Covariances* | | | | | | | | |
|  | ICPT & Slope AUDIT | -0.038 | 0.043 | -0.879 | 0.379 | -0.122 | 0.047 | -0.259 |  |
|  | STR 1 & 2 | -0.005 | 0.074 | -0.063 | 0.950 | -0.150 | 0.141 | -0.005 |  |
|  | STR 2 & 3 | 0.130 | 0.081 | 1.600 | 0.110 | -0.029 | 0.289 | 0.130 |  |
|  | STR 1 & 3 | -0.072 | 0.093 | -0.773 | 0.439 | -0.253 | 0.110 | -0.072 |  |
|  | STR 1 & Slope AUDIT | -0.013 | 0.030 | -0.437 | 0.662 | -0.072 | 0.046 | -0.041 |  |
|  | STR 2 & Slope AUDIT | -0.039 | 0.026 | -1.505 | 0.132 | -0.091 | 0.012 | -0.122 |  |
|  | STR 3 & Slope AUDIT | 0.120 | 0.045 | 2.654 | 0.008 | 0.031 | 0.209 | 0.374 |  |
|  | *Intercepts* | | | | | | | | |
|  | ICPT AUDIT | -0.644 | 0.043 | -15.134 | < 0.001 | -0.727 | -0.560 | -1.417 |  |
|  | Slope AUDIT | 0.386 | 0.030 | 12.786 | < 0.001 | 0.327 | 0.446 | 1.201 |  |
|  | *Regressions* | | | | | | | | |
|  | STR 1 → AUDIT 1 | -0.070 | 0.042 | -1.663 | 0.096 | -0.152 | 0.012 | -0.070 |  |
|  | STR 2 → AUDIT 2 | -0.073 | 0.075 | -0.965 | 0.335 | -0.220 | 0.075 | -0.073 |  |
|  | STR 3 → AUDIT 3 | -0.108 | 0.091 | -1.188 | 0.235 | -0.286 | 0.070 | -0.108 |  |
|  | *Residual variances* | | | | | | | | |
|  | ICPT AUDIT | 0.206 | 0.118 | 1.750 | 0.080 | -0.025 | 0.438 | 1.000 |  |
|  | Slope AUDIT | 0.103 | 0.024 | 4.296 | < 0.001 | 0.056 | 0.151 | 1.000 |  |
|  | AUDIT 1 | 0.052 | 0.116 | 0.453 | 0.650 | -0.174 | 0.279 | 0.052 |  |
|  | AUDIT 2 | 0.510 | 0.077 | 6.606 | 3.96E-11 | 0.359 | 0.661 | 0.510 |  |
|  | AUDIT 3 | 0.210 | 0.102 | 2.064 | 0.039 | 0.011 | 0.409 | 0.210 |  |
|  | STR 1 | 1.020 | 0.121 | 8.456 | < 0.001 | 0.783 | 1.256 | 1.020 |  |
|  | STR 2 | 0.776 | 0.092 | 8.456 | < 0.001 | 0.596 | 0.956 | 0.776 |  |
|  | STR 3 | 1.197 | 0.142 | 8.456 | < 0.001 | 0.919 | 1.474 | 1.197 |  |

*Note:* STR = Striatum, SU = single-update prediction error; AUDIT = Alcohol Use Disorder Identification Test (Saunders, Aasland, Babor, de la Fuente, & Grant, 1993)

Table S24: Latent growth curve model estimates for single-update prediction error in left striatum as time-varying covariate of drinking

| **Model** |  | **Estimate** | **SE** | **Z** | **p-value** | **95 % CI lower** | **95% CI upper** | **Standardized Estimate** |
| --- | --- | --- | --- | --- | --- | --- | --- | --- |
| Striatum left (SU) | *Covariances* | | | | | | | |
|  | ICPT & Slope AUDIT | -0.037 | 0.044 | -0.828 | 0.407 | -0.124 | 0.050 | -0.263 |
|  | STR 1 & 2 | 0.054 | 0.083 | 0.652 | 0.514 | -0.109 | 0.217 | 0.055 |
|  | STR 2 & 3 | 0.047 | 0.083 | 0.566 | 0.571 | -0.116 | 0.210 | 0.047 |
|  | STR 1 & 3 | 0.042 | 0.083 | 0.503 | 0.615 | -0.121 | 0.205 | 0.042 |
|  | STR 1 & Slope AUDIT | 0.044 | 0.029 | 1.526 | 0.127 | -0.012 | 0.100 | 0.142 |
|  | STR 2 & Slope AUDIT | -0.014 | 0.028 | -0.490 | 0.624 | -0.068 | 0.041 | -0.044 |
|  | STR 3 & Slope AUDIT | 0.024 | 0.040 | 0.597 | 0.550 | -0.054 | 0.102 | 0.077 |
|  | *Intercepts* | | | | | | | |
|  | ICPT AUDIT | -0.635 | 0.044 | -14.575 | < 0.001 | -0.720 | -0.550 | -1.411 |
|  | Slope AUDIT | 0.376 | 0.032 | 11.923 | < 0.001 | 0.314 | 0.438 | 1.211 |
|  | *Regressions* | | | | | | | |
|  | STR 1 → AUDIT 1 | -0.040 | 0.042 | -0.953 | 0.341 | -0.122 | 0.042 | -0.040 |
|  | STR 2 → AUDIT 2 | -0.053 | 0.078 | -0.681 | 0.496 | -0.206 | 0.100 | -0.053 |
|  | STR 3 → AUDIT 3 | -0.064 | 0.093 | -0.686 | 0.492 | -0.245 | 0.118 | -0.064 |
|  | *Residual variances* | | | | | | | |
|  | ICPT AUDIT | 0.203 | 0.122 | 1.666 | 0.096 | -0.036 | 0.441 | 1.000 |
|  | Slope AUDIT | 0.097 | 0.023 | 4.158 | < 0.001 | 0.051 | 0.142 | 1.000 |
|  | AUDIT 1 | 0.059 | 0.119 | 0.499 | 0.618 | -0.174 | 0.293 | 0.059 |
|  | AUDIT 2 | 0.516 | 0.079 | 6.530 | 6.56E-11 | 0.361 | 0.671 | 0.516 |
|  | AUDIT 3 | 0.209 | 0.106 | 1.962 | 0.050 | 0.000 | 0.417 | 0.209 |
|  | STR 1 | 1.060 | 0.125 | 8.456 | < 0.001 | 0.814 | 1.306 | 1.060 |
|  | STR 2 | 0.702 | 0.083 | 8.456 | < 0.001 | 0.539 | 0.865 | 0.702 |
|  | STR 3 | 1.005 | 0.119 | 8.456 | < 0.001 | 0.772 | 1.238 | 1.005 |

*Note:* STR = Striatum , SU = single-update prediction error; AUDIT = Alcohol Use Disorder Identification Test (Saunders, Aasland, Babor, de la Fuente, & Grant, 1993)

Table S25: Latent growth curve model estimates for single-update prediction error in right insula as time-varying covariate of drinking

| **Model** |  | **Estimate** | **SE** | **Z** | ***p*-value** | **95 % CI lower** | **95% CI upper** | **Standardized Estimate** |
| --- | --- | --- | --- | --- | --- | --- | --- | --- |
| Insula right (SU) | *Covariances* | | | | | | | |
|  | ICPT & Slope AUDIT | -0.030 | 0.043 | -0.699 | 0.484 | -0.114 | 0.054 | -0.219 |
|  | INS 1 & 2 | 0.026 | 0.083 | 0.311 | 0.756 | -0.137 | 0.189 | 0.026 |
|  | INS 2 & 3 | 0.091 | 0.079 | 1.147 | 0.251 | -0.064 | 0.245 | 0.091 |
|  | INS 1 & 3 | 0.143 | 0.088 | 1.625 | 0.104 | -0.029 | 0.315 | 0.143 |
|  | INS 1 & Slope AUDIT | 0.026 | 0.031 | 0.857 | 0.392 | -0.034 | 0.087 | 0.083 |
|  | INS 2 & Slope AUDIT | -0.013 | 0.028 | -0.469 | 0.639 | -0.067 | 0.041 | -0.041 |
|  | INS 3 & Slope AUDIT | 0.070 | 0.041 | 1.716 | 0.086 | -0.010 | 0.149 | 0.219 |
|  | *Intercepts* | | | | | | | |
|  | ICPT AUDIT | -0.647 | 0.043 | -15.162 | < 0.001 | -0.730 | -0.563 | -1.507 |
|  | Slope AUDIT | 0.391 | 0.030 | 12.972 | < 0.001 | 0.332 | 0.450 | 1.227 |
|  | *Regressions* | | | | | | | |
|  | INS 1 → AUDIT 1 | -0.046 | 0.040 | -1.138 | 0.255 | -0.124 | 0.033 | -0.046 |
|  | INS 2 → AUDIT 2 | -0.041 | 0.071 | -0.581 | 0.561 | -0.179 | 0.097 | -0.041 |
|  | INS 3 → AUDIT 3 | -0.186 | 0.099 | -1.881 | 0.060 | -0.381 | 0.008 | -0.186 |
|  | *Residual variances* | | | | | | | |
|  | ICPT AUDIT | 0.184 | 0.118 | 1.567 | 0.117 | -0.046 | 0.414 | 1.000 |
|  | Slope AUDIT | 0.101 | 0.024 | 4.280 | < 0.001 | 0.055 | 0.148 | 1.000 |
|  | AUDIT 1 | 0.076 | 0.116 | 0.655 | 0.513 | -0.151 | 0.303 | 0.076 |
|  | AUDIT 2 | 0.517 | 0.079 | 6.581 | 4.67E-11 | 0.363 | 0.671 | 0.517 |
|  | AUDIT 3 | 0.182 | 0.104 | 1.758 | 0.079 | -0.021 | 0.385 | 0.182 |
|  | INS 1 | 1.101 | 0.130 | 8.456 | < 0.001 | 0.846 | 1.356 | 1.101 |
|  | INS 2 | 0.900 | 0.106 | 8.456 | < 0.001 | 0.691 | 1.108 | 0.900 |
|  | INS 3 | 0.982 | 0.116 | 8.456 | < 0.001 | 0.755 | 1.210 | 0.982 |

*Note: INS = Insula, SU = single-update prediction error* ; AUDIT = Alcohol Use Disorder Identification Test (Saunders, Aasland, Babor, de la Fuente, & Grant, 1993)

Table S26: Latent growth curve model estimates for single-update prediction error in left insula as time-varying covariate of drinking

| **Model** |  | **Estimate** | **SE** | **Z** | **p-value** | **95 % CI lower** | **95% CI upper** | **Standardized Estimate** |
| --- | --- | --- | --- | --- | --- | --- | --- | --- |
| Insula left (SU) | *Covariances* | | | | | | | |
|  | ICPT & Slope AUDIT | -0.042 | 0.044 | -0.937 | 0.349 | -0.129 | 0.045 | -0.279 |
|  | INS 1 & 2 | 0.002 | 0.117 | 0.018 | 0.985 | -0.226 | 0.231 | 0.002 |
|  | INS 2 & 3 | 0.039 | 0.041 | 0.954 | 0.340 | -0.041 | 0.120 | 0.039 |
|  | INS 1 & 3 | -0.050 | 0.041 | -1.221 | 0.222 | -0.131 | 0.030 | -0.050 |
|  | INS 1 & Slope AUDIT | 0.035 | 0.034 | 1.028 | 0.304 | -0.032 | 0.101 | 0.109 |
|  | INS 2 & Slope AUDIT | -0.045 | 0.033 | -1.342 | 0.180 | -0.111 | 0.021 | -0.141 |
|  | INS 3 & Slope AUDIT | 0.006 | 0.017 | 0.343 | 0.731 | -0.027 | 0.039 | 0.018 |
|  | *Intercepts* | | | | | | | |
|  | ICPT AUDIT | -0.650 | 0.043 | -15.229 | < 0.001 | -0.734 | -0.567 | -1.392 |
|  | Slope AUDIT | 0.399 | 0.031 | 12.937 | < 0.001 | 0.339 | 0.460 | 1.253 |
|  | *Regressions* | | | | | | | |
|  | INS 1 → AUDIT 1 | -0.051 | 0.035 | -1.425 | 0.154 | -0.120 | 0.019 | -0.051 |
|  | INS 2 → AUDIT 2 | -0.023 | 0.057 | -0.401 | 0.688 | -0.133 | 0.088 | -0.023 |
|  | INS 3 → AUDIT 3 | -0.298 | 0.236 | -1.262 | 0.207 | -0.762 | 0.165 | -0.298 |
|  | *Residual variances* | | | | | | | |
|  | ICPT AUDIT | 0.218 | 0.123 | 1.777 | 0.076 | -0.022 | 0.459 | 1.000 |
|  | Slope AUDIT | 0.102 | 0.024 | 4.320 | 1.56E-05 | 0.055 | 0.148 | 1.000 |
|  | AUDIT 1 | 0.040 | 0.120 | 0.337 | 0.736 | -0.195 | 0.276 | 0.040 |
|  | AUDIT 2 | 0.537 | 0.081 | 6.659 | 2.76E-11 | 0.379 | 0.695 | 0.537 |
|  | AUDIT 3 | 0.163 | 0.103 | 1.582 | 0.114 | -0.039 | 0.364 | 0.163 |
|  | INS 1 | 1.396 | 0.165 | 8.456 | < 0.001 | 1.072 | 1.719 | 1.396 |
|  | INS 2 | 1.393 | 0.165 | 8.456 | < 0.001 | 1.070 | 1.716 | 1.393 |
|  | INS 3 | 0.173 | 0.020 | 8.456 | < 0.001 | 0.133 | 0.213 | 0.173 |

*Note: INS = Insula, SU = single-update prediction error*; AUDIT = Alcohol Use Disorder Identification Test (Saunders, Aasland, Babor, de la Fuente, & Grant, 1993)

Table S27: Latent growth curve model estimates for single-update prediction error in MFG as time-varying covariate of drinking

| **Model** |  | **Estimate** | **SE** | **Z** | **p-value** | **95 % CI lower** | **95% CI upper** | **Standardized Estimate** |
| --- | --- | --- | --- | --- | --- | --- | --- | --- |
| MFG (SU) | *Covariances* | | | | | | | |
|  | ICPT & Slope AUDIT | -0.047 | 0.044 | -1.064 | 0.287 | -0.133 | 0.039 | -0.311 |
|  | MFG 1 & 2 | 0.092 | 0.074 | 1.239 | 0.215 | -0.053 | 0.237 | 0.092 |
|  | MFG 2 & 3 | 0.112 | 0.075 | 1.489 | 0.137 | -0.035 | 0.259 | 0.112 |
|  | MFG 1 & 3 | 0.520 | 0.106 | 4.900 | < 0.001 | 0.312 | 0.728 | 0.520 |
|  | MFG 1 & Slope AUDIT | -0.021 | 0.034 | -0.605 | 0.545 | -0.087 | 0.046 | -0.063 |
|  | MFG 2 & Slope AUDIT | 0.020 | 0.024 | 0.848 | 0.396 | -0.026 | 0.066 | 0.061 |
|  | MFG 3 & Slope AUDIT | -0.009 | 0.045 | -0.203 | 0.839 | -0.097 | 0.079 | -0.028 |
|  | *Intercepts* | | | | | | | |
|  | ICPT AUDIT | -0.645 | 0.042 | -15.262 | < 0.001 | -0.728 | -0.562 | -1.402 |
|  | Slope AUDIT | 0.387 | 0.030 | 12.998 | < 0.001 | 0.329 | 0.445 | 1.182 |
|  | *Regressions* | | | | | | | |
|  | MFG 1 → AUDIT 1 | -0.070 | 0.039 | -1.789 | 0.074 | -0.147 | 0.007 | -0.070 |
|  | MFG 2 → AUDIT 2 | -0.179 | 0.080 | -2.230 | 0.026 | -0.336 | -0.022 | -0.179 |
|  | MFG 3 → AUDIT 3 | -0.117 | 0.093 | -1.259 | 0.208 | -0.300 | 0.065 | -0.117 |
|  | *Residual variances* | | | | | | | |
|  | ICPT AUDIT | 0.212 | 0.122 | 1.733 | 0.083 | -0.028 | 0.451 | 1.000 |
|  | Slope AUDIT | 0.107 | 0.023 | 4.590 | < 0.001 | 0.061 | 0.153 | 1.000 |
|  | AUDIT 1 | 0.044 | 0.120 | 0.364 | 0.716 | -0.191 | 0.278 | 0.044 |
|  | AUDIT 2 | 0.529 | 0.079 | 6.673 | < 0.001 | 0.374 | 0.685 | 0.529 |
|  | AUDIT 3 | 0.136 | 0.102 | 1.336 | 0.182 | -0.063 | 0.335 | 0.136 |
|  | MFG 1 | 1.147 | 0.136 | 8.456 | < 0.001 | 0.881 | 1.413 | 1.147 |
|  | MFG 2 | 0.679 | 0.080 | 8.456 | < 0.001 | 0.521 | 0.836 | 0.679 |
|  | MFG 3 | 1.167 | 0.138 | 8.456 | < 0.001 | 0.896 | 1.437 | 1.167 |

*Note: MFG = medial frontal gyrus, SU = single-update prediction error* ; AUDIT = Alcohol Use Disorder Identification Test (Saunders, Aasland, Babor, de la Fuente, & Grant, 1993)

Table S28: Latent growth curve model estimates for double-update prediction error in right striatum as time-varying covariate of drinking

| **Model** |  | **Estimate** | **SE** | **Z** | **p-value** | **95 % CI lower** | **95% CI upper** | **Standardized Estimate** |
| --- | --- | --- | --- | --- | --- | --- | --- | --- |
| Striatum right (DU) | *Covariances* | | | | | | | |
|  | ICPT & Slope AUDIT | -0.025 | 0.043 | -0.579 | 0.563 | -0.110 | 0.060 | -0.202 |
|  | STR 1 & 2 | 0.011 | 0.093 | 0.122 | 0.903 | -0.171 | 0.194 | 0.011 |
|  | STR 2 & 3 | 0.051 | 0.079 | 0.652 | 0.514 | -0.103 | 0.206 | 0.051 |
|  | STR 1 & 3 | 0.185 | 0.077 | 2.390 | 0.017 | 0.033 | 0.336 | 0.185 |
|  | STR 1 & Slope AUDIT | 0.069 | 0.031 | 2.247 | 0.025 | 0.009 | 0.130 | 0.230 |
|  | STR 2 & Slope AUDIT | 0.040 | 0.031 | 1.291 | 0.197 | -0.021 | 0.102 | 0.134 |
|  | STR 3 & Slope AUDIT | -0.043 | 0.034 | -1.247 | 0.212 | -0.111 | 0.025 | -0.142 |
|  | *Intercepts* | | | | | | | |
|  | ICPT AUDIT | -0.643 | 0.043 | -1.51E+01 | < 0.001 | -0.727 | -0.559 | -1.56E+00 |
|  | Slope AUDIT | 0.386 | 0.030 | 13.011 | < 0.001 | 0.328 | 0.444 | 1.279 |
|  | *Regressions* | | | | | | | |
|  | STR 1 → AUDIT 1 | -0.020 | 0.041 | -0.485 | 0.628 | -0.101 | 0.061 | -0.020 |
|  | STR 2 → AUDIT 2 | 0.148 | 0.062 | 2.385 | 0.017 | 0.026 | 0.270 | 0.148 |
|  | STR 3 → AUDIT 3 | 0.117 | 0.113 | 1.040 | 0.298 | -0.104 | 0.339 | 0.117 |
|  | *Residual variances* | | | | | | | |
|  | ICPT AUDIT | 0.170 | 0.118 | 1.446 | 0.148 | -0.061 | 0.401 | 1.000 |
|  | Slope AUDIT | 0.091 | 0.023 | 3.951 | < 0.001 | 0.046 | 0.136 | 1.000 |
|  | AUDIT 1 | 0.092 | 0.117 | 0.788 | 0.431 | -0.137 | 0.320 | 0.092 |
|  | AUDIT 2 | 0.478 | 0.074 | 6.457 | 1.07E-10 | 0.333 | 0.623 | 0.478 |
|  | AUDIT 3 | 0.243 | 0.102 | 2.394 | 0.017 | 0.044 | 0.442 | 0.243 |
|  | STR 1 | 1.071 | 0.127 | 8.456 | < 0.001 | 0.823 | 1.319 | 1.071 |
|  | STR 2 | 1.156 | 0.137 | 8.456 | < 0.001 | 0.888 | 1.424 | 1.156 |
|  | STR 3 | 0.766 | 0.091 | 8.456 | < 0.001 | 0.588 | 0.943 | 0.766 |

*Note:* STR = Striatum, DU = double-update prediction error; AUDIT = Alcohol Use Disorder Identification Test (Saunders, Aasland, Babor, de la Fuente, & Grant, 1993)

Table S29: Latent growth curve model estimates for double-update prediction error in left striatum as time-varying covariate of drinking

| **Model** |  | **Estimate** | **SE** | **Z** | **p-value** | **95 % CI lower** | **95% CI upper** | **Standardized Estimate** |
| --- | --- | --- | --- | --- | --- | --- | --- | --- |
| Striatum left (DU) | *Covariances* | | | | | | | |
|  | ICPT & Slope AUDIT | -0.032 | 0.043 | -0.734 | 0.463 | -0.116 | 0.053 | -0.230 |
|  | STR 1 & 2 | -0.036 | 0.089 | -0.399 | 0.690 | -0.211 | 0.139 | -0.036 |
|  | STR 2 & 3 | -0.005 | 0.082 | -0.063 | 0.950 | -0.166 | 0.156 | -0.005 |
|  | STR 1 & 3 | 0.072 | 0.078 | 0.917 | 0.359 | -0.081 | 0.225 | 0.072 |
|  | STR 1 & Slope AUDIT | 0.046 | 0.030 | 1.564 | 0.118 | -0.012 | 0.104 | 0.146 |
|  | STR 2 & Slope AUDIT | -0.010 | 0.031 | -0.324 | 0.746 | -0.070 | 0.050 | -0.031 |
|  | STR 3 & Slope AUDIT | -0.071 | 0.038 | -1.893 | 0.058 | -0.145 | 0.003 | -0.226 |
|  | *Intercepts* | | | | | | | |
|  | ICPT AUDIT | -0.643 | 0.043 | -15.060 | < 0.001 | -0.727 | -0.560 | -1.477 |
|  | Slope AUDIT | 0.386 | 0.030 | 12.915 | < 0.001 | 0.327 | 0.445 | 1.219 |
|  | *Regressions* | | | | | | | |
|  | STR 1 → AUDIT 1 | -0.033 | 0.042 | -0.775 | 0.438 | -0.115 | 0.050 | -0.033 |
|  | STR 2 → AUDIT 2 | 0.106 | 0.064 | 1.664 | 0.096 | -0.019 | 0.230 | 0.106 |
|  | STR 3 → AUDIT 3 | 0.089 | 0.106 | 0.838 | 0.402 | -0.119 | 0.298 | 0.089 |
|  | *Residual variances* | | | | | | | |
|  | ICPT AUDIT | 0.190 | 0.118 | 1.601 | 0.109 | -0.043 | 0.422 | 1.000 |
|  | Slope AUDIT | 0.100 | 0.024 | 4.234 | 2.30E-05 | 0.054 | 0.147 | 1.000 |
|  | AUDIT 1 | 0.072 | 0.117 | 0.615 | 0.539 | -0.157 | 0.300 | 0.072 |
|  | AUDIT 2 | 0.515 | 0.078 | 6.611 | 3.81E-11 | 0.363 | 0.668 | 0.515 |
|  | AUDIT 3 | 0.185 | 0.102 | 1.817 | 0.069 | -0.015 | 0.385 | 0.185 |
|  | STR 1 | 1.013 | 0.120 | 8.456 | < 0.001 | 0.778 | 1.248 | 1.013 |
|  | STR 2 | 1.124 | 0.133 | 8.456 | < 0.001 | 0.864 | 1.385 | 1.124 |
|  | STR 3 | 0.856 | 0.101 | 8.456 | < 0.001 | 0.657 | 1.054 | 0.856 |

*Note:* STR = Striatum , DU = double-update prediction error ; AUDIT = Alcohol Use Disorder Identification Test (Saunders, Aasland, Babor, de la Fuente, & Grant, 1993)

Table S30: Latent growth curve model estimates for double-update prediction error in right insula as time-varying covariate of drinking

| **Model** |  | **Estimate** | **SE** | **Z** | **p-value** | **95 % CI lower** | **95% CI upper** | **Standardized Estimate** |
| --- | --- | --- | --- | --- | --- | --- | --- | --- |
| Insula right (DU) | *Covariances* | | | | | | | |
|  | ICPT & Slope AUDIT | -0.033 | 0.044 | -0.765 | 0.444 | -0.119 | 0.052 | -0.241 |
|  | INS 1 & 2 | 0.155 | 0.089 | 1.741 | 0.082 | -0.019 | 0.329 | 0.155 |
|  | INS 2 & 3 | 0.026 | 0.082 | 0.312 | 0.755 | -0.135 | 0.186 | 0.026 |
|  | INS 1 & 3 | 0.065 | 0.080 | 0.815 | 0.415 | -0.091 | 0.221 | 0.065 |
|  | INS 1 & Slope AUDIT | -0.012 | 0.029 | -0.397 | 0.691 | -0.069 | 0.045 | -0.037 |
|  | INS 2 & Slope AUDIT | 0.022 | 0.030 | 0.758 | 0.449 | -0.036 | 0.081 | 0.071 |
|  | INS 3 & Slope AUDIT | -0.031 | 0.039 | -0.812 | 0.417 | -0.107 | 0.044 | -0.100 |
|  | *Intercepts* | | | | | | | |
|  | ICPT AUDIT | -0.644 | 0.043 | -15.084 | < 0.001 | -0.727 | -0.560 | -1.466 |
|  | Slope AUDIT | 0.386 | 0.030 | 13.011 | < 0.001 | 0.327 | 0.444 | 1.222 |
|  | *Regressions* | | | | | | | |
|  | INS 1 → AUDIT 1 | -0.045 | 0.042 | -1.076 | 0.282 | -0.127 | 0.037 | -0.045 |
|  | INS 2 → AUDIT 2 | 0.019 | 0.065 | 0.300 | 0.764 | -0.107 | 0.146 | 0.019 |
|  | INS 3 → AUDIT 3 | 0.043 | 0.107 | 0.398 | 0.690 | -0.168 | 0.253 | 0.043 |
|  | *Residual variances* | | | | | | | |
|  | ICPT AUDIT | 0.193 | 0.120 | 1.607 | 0.108 | -0.042 | 0.428 | 1.000 |
|  | Slope AUDIT | 0.099 | 0.023 | 4.251 | 2.13E-05 | 0.054 | 0.145 | 1.000 |
|  | AUDIT 1 | 0.068 | 0.118 | 0.575 | 0.565 | -0.163 | 0.299 | 0.068 |
|  | AUDIT 2 | 0.535 | 0.081 | 6.629 | 3.37E-11 | 0.377 | 0.693 | 0.535 |
|  | AUDIT 3 | 0.174 | 0.105 | 1.660 | 0.097 | -0.031 | 0.379 | 0.174 |
|  | INS 1 | 1.022 | 0.121 | 8.456 | < 0.001 | 0.785 | 1.259 | 1.022 |
|  | INS 2 | 1.085 | 0.128 | 8.456 | < 0.001 | 0.833 | 1.336 | 1.085 |
|  | INS 3 | 0.886 | 0.105 | 8.456 | < 0.001 | 0.681 | 1.092 | 0.886 |

*Note: INS = Insula, DU = double-update prediction error* ; AUDIT = Alcohol Use Disorder Identification Test (Saunders, Aasland, Babor, de la Fuente, & Grant, 1993)

Table S31: Latent growth curve model estimates for double-update prediction error in left insula as time-varying covariate of drinking

| **Model** |  | **Estimate** | **SE** | **Z** | **p-value** | **95 % CI lower** | **95% CI upper** | **Standardized Estimate** |
| --- | --- | --- | --- | --- | --- | --- | --- | --- |
| Insula left (DU) | *Covariances* | | | | | | | |
|  | ICPT & Slope AUDIT | -0.033 | 0.044 | -0.748 | 0.455 | -0.119 | 0.053 | -0.234 |
|  | INS 1 & 2 | 0.165 | 0.086 | 1.920 | 0.055 | -0.003 | 0.334 | 0.165 |
|  | INS 2 & 3 | 0.001 | 0.080 | 0.018 | 0.986 | -0.156 | 0.159 | 0.001 |
|  | INS 1 & 3 | 0.133 | 0.086 | 1.553 | 0.120 | -0.035 | 0.300 | 0.133 |
|  | INS 1 & Slope AUDIT | 0.010 | 0.030 | 0.332 | 0.740 | -0.049 | 0.069 | 0.032 |
|  | INS 2 & Slope AUDIT | 0.040 | 0.028 | 1.428 | 0.153 | -0.015 | 0.095 | 0.128 |
|  | INS 3 & Slope AUDIT | -0.052 | 0.040 | -1.301 | 0.193 | -0.131 | 0.026 | -0.166 |
|  | *Intercepts* | | | | | | | |
|  | ICPT AUDIT | -0.644 | 0.043 | -15.055 | < 0.001 | -0.728 | -0.560 | -1.436 |
|  | Slope AUDIT | 0.386 | 0.029 | 13.075 | < 0.001 | 0.328 | 0.443 | 1.229 |
|  | *Regressions* | | | | | | | |
|  | INS 1 → AUDIT 1 | -0.040 | 0.041 | -0.980 | 0.327 | -0.121 | 0.040 | -0.040 |
|  | INS 2 → AUDIT 2 | -0.002 | 0.070 | -0.022 | 0.982 | -0.139 | 0.135 | -0.002 |
|  | INS 3 → AUDIT 3 | 0.034 | 0.102 | 0.329 | 0.742 | -0.167 | 0.234 | 0.034 |
|  | *Residual variances* | | | | | | | |
|  | ICPT AUDIT | 0.201 | 0.121 | 1.656 | 0.098 | -0.037 | 0.439 | 1.000 |
|  | Slope AUDIT | 0.099 | 0.024 | 4.163 | < 0.001 | 0.052 | 0.145 | 1.000 |
|  | AUDIT 1 | 0.061 | 0.119 | 0.508 | 0.611 | -0.173 | 0.294 | 0.061 |
|  | AUDIT 2 | 0.531 | 0.080 | 6.596 | < 0.001 | 0.373 | 0.688 | 0.531 |
|  | AUDIT 3 | 0.183 | 0.105 | 1.739 | 0.082 | -0.023 | 0.389 | 0.183 |
|  | INS 1 | 1.071 | 0.127 | 8.456 | < 0.001 | 0.823 | 1.319 | 1.071 |
|  | INS 2 | 0.962 | 0.114 | 8.456 | < 0.001 | 0.739 | 1.185 | 0.962 |
|  | INS 3 | 0.960 | 0.114 | 8.456 | < 0.001 | 0.738 | 1.183 | 0.960 |

*Note: INS = Insula, DU = double-update prediction error* ; AUDIT = Alcohol Use Disorder Identification Test (Saunders, Aasland, Babor, de la Fuente, & Grant, 1993)

## Correlation of cumulative alcohol consumption with ROI signals

Table S32: Pearson’s correlation r between cumulative alcohol consumption and difference scores (age 18 – age 14) of ROI signals

| Variable | M | SD | 1 |
| --- | --- | --- | --- |
| 1. Cumulative alcohol consumption | 5580.2 | 6582.06 |  |
|  |  |  |  |
| 2. Difference score ventromedial prefrontal cortex (choice probability) | 0.06 | 0.49 | 0.14 |
|  |  |  | [-.02, .30] |
| 3. Difference score posterior cingulate cortex (choice probability) | 0.1 | 0.69 | 0.09 |
|  |  |  | [-.08, .25] |
| 4. Difference score right striatum (SU) | 0.02 | 1.68 | .18* |
|  |  |  | [.01, .33] |
| 5. Difference score left striatum (SU) | -0.71 | 1.7 | 0.07 |
|  |  |  | [-.10, .23] |
| 6. Difference score right insula (SU) | 0.17 | 1.6 | .19* |
|  |  |  | [.02, .34] |
| 7. Difference score left insula (SU) | 0.24 | 1.22 | 0.04 |
|  |  |  | [-.12, .21] |
| 8. Difference score right striatum (DU) | -0.01 | 1.4 | -.25** |
|  |  |  | [-.40, -.09] |
| 9. Difference score left striatum (DU) | -0.01 | 1.37 | -.19* |
|  |  |  | [-.35, -.03] |
| 10. Difference right insula (DU) | 0.01 | 1.6 | -0.16 |
|  |  |  | [-.32, .00] |
| 11. Difference score left insula (DU) | 0.01 | 1.52 | -.23** |
|  |  |  | [-.38, -.07] |
| 12. Difference score medial frontal gyrus (SU) | 0.01 | 0.61 | -0.01 |
|  |  |  | [-.18, .15] |

## fMRI reliability – Intraclass coefficients

Table S33: Intraclass coefficient (ICC) for signals extracted from regions of interest for the main effects of respective contrasts

|  | **Longitudinal** | | | | **Split-half age 14** | | | | **Split-half age 16** | | | | **Split-half age 18** | | | | **Average of split-half ICC** |
| --- | --- | --- | --- | --- | --- | --- | --- | --- | --- | --- | --- | --- | --- | --- | --- | --- | --- |
| **Variable** | **ICC** | **CI** | | **p-value** | **ICC** | **CI** | | **p-value** | **ICC** | **CI** | | **p-value** | **ICC** | **CI** | | **p-value** |  |
|  |  | **lower** | **upper** |  |  | **lower** | **upper** |  |  | **lower** | **upper** |  |  | **lower** | **upper** |  |  |
| Left Insula  (DU) | 0.100 | 0.003 | 0.208 | 0.022 | 0.065 | -0.1 | 0.226 | 0.219 | 0.132 | -0.032 | 0.289 | 0.057 | 0.192 | 0.029 | 0.345 | 0.011 | 0.130 |
| Right Insula  (DU) | 0.082 | -0.014 | 0.189 | 0.048 | -0.029 | -0.191 | 0.136 | 0.633 | 0.185 | 0.022 | 0.338 | 0.013 | 0.171 | 0.008 | 0.326 | 0.02 | 0.110 |
| Right Striatum  (DU) | 0.083 | -0.013 | 0.190 | 0.046 | 0.066 | -0.98 | 0.228 | 0.214 | 0.170 | 0.006 | 0.324 | 0.021 | 0.126 | -0.038 | 0.284 | 0.066 | 0.121 |
| Left Striatum  (DU) | 0.010 | -0.079 | 0.113 | 0.41 | 0.165 | 0.001 | 0.32 | 0.024 | 0.178 | 0.014 | 0.332 | 0.017 | 0.134 | -0.031 | 0.291 | 0.055 | 0.159 |
| Left Insula  (SU) | -0.003 | -0.091 | 0.099 | 0.519 | 0.107 | -0.058 | 0.266 | 0.101 | -0.152 | -0.308 | 0.012 | 0.965 | 0.172 | 0.009 | 0.327 | 0.02 | 0.043 |
| Right Insula  (SU) | 0.087 | -0.009 | 0.194 | 0.039 | 0.017 | -0.147 | 0.18 | 0.419 | -0.091 | -0.251 | 0.074 | 0.86 | 0.202 | 0.039 | 0.354 | 0.008 | 0.043 |
| Right Striatum  (SU) | 0.018 | -0.072 | 0.121 | 0.35 | -0.012 | -0.175 | 0.152 | 0.555 | -0.054 | -0.216 | 0.11 | 0.741 | 0.033 | -0.131 | 0.196 | 0.346 | -0.011 |
| Left Striatum  (SU) | 0.047 | -0.046 | 0.152 | 0.165 | -0.122 | -0.28 | 0.043 | 0.927 | -0.163 | -0.318 | 0.001 | 0.974 | 0.000 | -0.159 | 0.168 | 0.478 | -0.095 |
| Medial Frontal Gyrus  (SU) | 0.242 | 0.138 | 0.351 | 9.60E-07 | 0.2 | 0.038 | 0.352 | 0.008 | 0.099 | -0.066 | 0.258 | 1.19E-01 | 0.334 | 0.18 | 0.471 | 2.20E-05 | 0.213 |
| vmPFC  (CP) | 0.160 | 0.059 | 0.269 | 0.001 | 0.039 | -0.126 | 0.201 | 0.322 | -0.200 | -0.652 | -0.038 | 0.992 | 0.167 | 0.003 | 0.322 | 0.023 | 0.002 |
| PCC  (CP) | 0.115 | 0.017 | 0.223 | 0.010 | 0.077 | -0.088 | 0.238 | 0.179 | -0.257 | -0.403 | -0.097 | 0.999 | 0.079 | -0.086 | 0.239 | 0.175 | -0.036 |

Note: CI = confidence interval, SU = Single-update prediction error, DU = Double-update prediction error, CP = Choice probability, vmPFC = ventromedial prefrontal cortex, PCC = posterior cingulate cortex

# References

1. Waltmann M, Schlagenhauf F, Deserno L. Sufficient reliability of the behavioral and computational readouts of a probabilistic reversal learning task. Behav Res Methods. 2022;54(6):2993-3014.

2. Huys QJM, Schad DJ. Emfit Matlab Script. 2015.

3. Huys QJ, Cools R, Golzer M, Friedel E, Heinz A, Dolan RJ, et al. Disentangling the roles of approach, activation and valence in instrumental and pavlovian responding. PLoS Comput Biol. 2011;7(4):e1002028.

4. Huys QJ, Eshel N, O'Nions E, Sheridan L, Dayan P, Roiser JP. Bonsai trees in your head: how the pavlovian system sculpts goal-directed choices by pruning decision trees. PLoS Comput Biol. 2012;8(3):e1002410.

5. Saunders JB, Aasland OG, Babor TF, de la Fuente JR, Grant M. Development of the Alcohol Use Disorders Identification Test (AUDIT): WHO Collaborative Project on Early Detection of Persons with Harmful Alcohol Consumption--II. Addiction. 1993;88(6):791-804.

6. Ashburner J, Friston KJ. Unified segmentation. Neuroimage. 2005;26(3):839-51.

7. Yarkoni T, Poldrack RA, Nichols TE, Van Essen DC, Wager TD. Large-scale automated synthesis of human functional neuroimaging data. Nat Methods. 2011;8(8):665-70.
